# Stereoselective Formation of Substituted 1,3-Dioxolanes through a Three-Component Assembly during the Oxidation of Alkenes with Hypervalent Iodine(III)

**DOI:** 10.3390/molecules200917041

**Published:** 2015-09-17

**Authors:** Mio Shimogaki, Morifumi Fujita, Takashi Sugimura

**Affiliations:** Graduate School of Material Science, University of Hyogo, Kohto, Kamigori, Hyogo 678-1297, Japan; E-Mails: rk14v001@stkt.u-hyogo.ac.jp (M.S.); sugimura@sci.u-hyogo.ac.jp (T.S.)

**Keywords:** hypervalent iodine, oxidation, dioxolane, dioxolanyl cation, stereoselective synthesis

## Abstract

Stereoselective formation of substituted 1,3-dioxolanes was achieved through an assembly of three components: alkene, carboxylic acid and silyl enol ether. The reaction proceeded via stereospecific generation of a 1,3-dioxolan-2-yl cation intermediate during oxidation of alkene substrates with hypervalent iodine. The stereoselective trapping of the cation intermediate with silyl enol ether completed the formation of the dioxolane product.

## 1. Introduction

**Figure 1:**
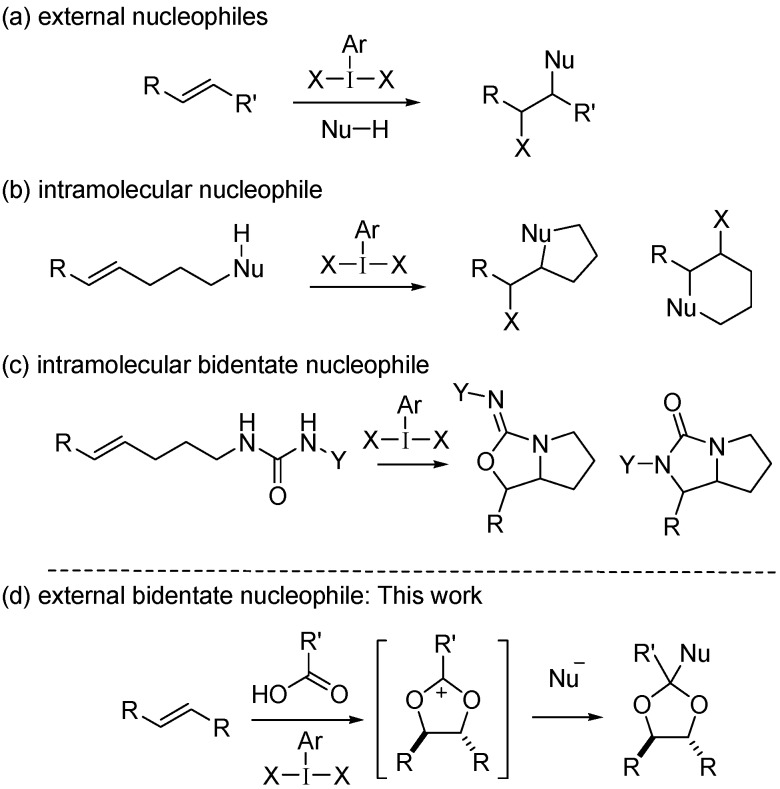
Difunctionalization of alkenes with hypervalent iodine(III) using (**a**) external nucleophiles; (**b**) intramolecular nucleophile; (**c**) intramolecular bidentate nucleophile; and (**d**) external bidentate nucleophile.

Oxidative difunctionalization of alkenes with hypervalent iodine reagents is one of the many attractive and powerful transformations, as well as halogenation, oxygenation, amidation and arylation (alkylation), for forming useful synthetic intermediates [1,2,3,4,5,6,7,8,9,10,11,12,13,14,15,16,17,18,19,20,21,22,23,24,25,26,27,28,29,30,31,32,33,34,35,36,37,38,39,40,41,42,43,44]. During oxidation, two nucleophiles are attached to the carbon-carbon double bond (Figure 1a) [20,21,22,23,24,25,26]. This approach has also been applied to the regio- and stereoselective preparation of heterocyclic compounds. An alkene substrate containing a simple intramolecular nucleophile provides a heterocyclic product (Figure 1b) [27,28,29,30,31,32,33,34,35,36,37,38,39,40]. When a bidentate nucleophile such as urea is introduced to the alkene substrate, bicyclic products are obtained (Figure 1c) [41,42,43,44]. To expand upon the diverse structures of the resulting heterocyclic products, we postulated that intermolecular heterocyclic formation would be achieved using an external bidentate nucleophile (Figure 1d) [45,46,47]. To demonstrate this concept, we focused on the 1,3-dioxolan-2-yl cation intermediate [48,49] generated during hypervalent-iodine-mediated Prévost and Woodward reactions [50,51,52,53,54,55,56,57,58]. In these reactions, a carboxylic acid acts as a bidentate nucleophile; however, the heterocyclic intermediate changes to a ring-opening product. To maintain the heterocyclic structure of dioxolane, a subsequent nucleophilic attack should occur at the 2-position to give a dioxolane product in stable form [59,60]. Herein, we describe the stereoselective preparation of substituted dioxolanes through condensation of three components: alkene, carboxylic acid and carbon nucleophile during the hypervalent-iodine-mediated Prévost and Woodward reaction. The stereoselectivity must be controlled by two steps: the oxidative formation of a 1,3-dioxolan-2-yl cation intermediate and its nucleophilic trapping (Figure 1d).

## 2. Results and Discussion

We initially selected *cis*-4-octene (**1a**) as a model substrate to test the feasibility of this transformation (Table 1). The reaction of **1a** with (diacetoxyiodo)benzene was initiated by injection of boron trifluoride diethyl etherate at −80 °C into a dichloromethane solution containing AcOH. After warming to −40 °C, dimethyl ketene silyl acetal **2a** was added to the reaction mixture to produce a dioxolane product **3a** as a single diastereomer (Table 1, entries 1–3). The yield of **3a** was affected by the amount of nucleophiles (acetic acid and **2a**), as well as the temperature of reaction termination; the maximum yield was 62% (entry 3). In the ^13^C-NMR of **3a**, ten signals were observed. This indicates that the adduct **3a** has *C*_s_ symmetry (*meso* configuration). Moreover, the stereochemistry of **3a** was determined to be the (2*r*)-configuration by nOe between *H*-4,5 and the dimethyl moiety (the illustration in Table 1), which was observed in an NMR NOESY experiment (Supplementary Materials (SM)). The corresponding chiral racemic isomer **3b** was selectively obtained from the reaction with *trans*-4-octene **1b** (entry 4). In the ^13^C-NMR spectrum of **3b**, fifteen signals were detected.

**Table 1:** Reaction of *cis*-4-octene **1a** and *trans*-4-octene **1b**^a^. 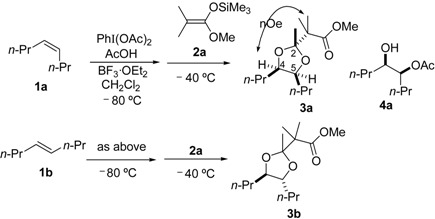

Entry	1	AcOH (mmol)	2a (mmol)	Quench Temp.	Yield (%)
1	**1a**	1.7	0.5	rt	**3a**, 2; **4a**, 58
2	**1a**	0.5	1.5	rt	**3a**, 46
3	**1a**	0.5	1.5	−30 °C	**3a**, 62
4	**1b**	0.5	1.5	−30 °C	**3b**, 48

^a^ Reaction was initiated at −80 °C in the presence of **1** (0.32 mmol), PhI(OAc)_2_ (0.40 mmol) and BF_3_·OEt_2_ (0.8 mmol) in CH_2_Cl_2_ (4 mL) and AcOH. Then, **2a** was added at −40 °C.

**Scheme 1:**
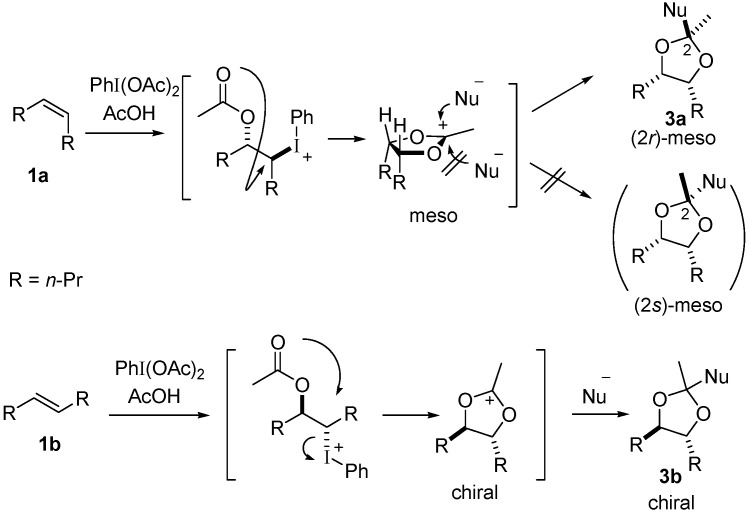
Plausible pathway for stereoselective formation of dioxolane **3**.

The stereospecific and stereoselective formation of the substituted dioxolane products can be explained by reaction pathways involving the 1,3-dioxolan-2-yl cation intermediate (Scheme 1). This intermediate was generated by participation of the neighboring acetoxy group in the oxidation of the alkene substrate with hypervalent iodine in the presence of AcOH. The neighboring group participation may cause stereospecific formation of the dioxolanyl cation intermediate, *i.e.*, the *cis* alkene **1a** forms the meso cation and the *trans*-alkene **1b** forms the chiral cation.

Stereoselectivity in nucleophilic trapping with **2a** affects the stereochemical outcome of the dioxolane products. The meso cation generated from the *cis* alkene has diastereofaces for the nucleophilic addition. Thus, the diastereoselective formation of the trapping product requires stereoface control in the nucleophilic attack, as illustrated in Scheme 1. The actual diastereoselective formation of the (2*r*)-meso product starting from *cis* alkene may be attributed to the steric fence provided by the 4,5-substituents of the meso cation. Owing to the *C*_2_ symmetry of the chiral cation generated from the *trans*-octene, the nucleophilic trapping of the chiral cation led to the chiral dioxolane product **3b** as a single diastereomer without any stereocontrol during the nucleophilic trapping.

**Scheme 2:**
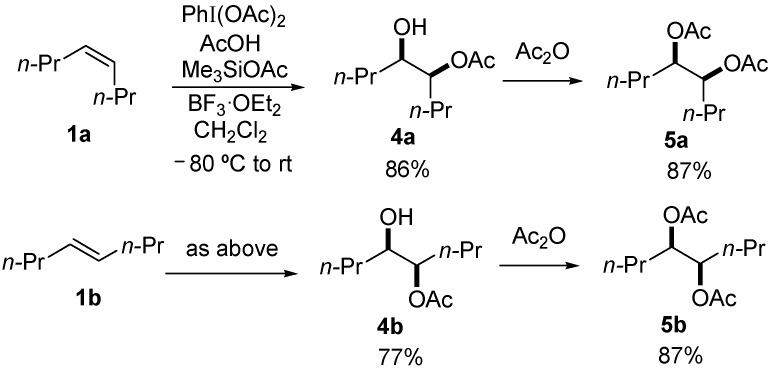
Simple dioxygenation of 4-octenes **1a** and **1b**.

The proposed reaction pathway (Scheme 1) was also supported by control experiments without the addition of the trapping reagent **2a** (Scheme 2). The acetoxyhydroxy products **4a** and **4b** were stereospecifically obtained from *cis*-octene **1a** and *trans*-octene **1b**, respectively; their stereochemical configurations were confirmed after transformation to diacetoxy products: meso form **5a** and racemic form **5b**. The stereochemical outcome obeyed that of a typical Woodward reaction: trapping of 1,3-dioxolan-2-yl cation with water at the 2-position and subsequent ring-opening reaction result in retention of configuration of the cation intermediate.

Under optimal conditions (Table 1, entries 3 and 4), the generality of the method was investigated (Table 2). Styrenes and cycloalkenes also successfully gave the dioxolane product **3** from reaction with dimethyl ketene silyl acetal **2a** (entries 1–6). Isopropenyloxytrimethylsilane (**2b**), 1-ethoxy-1-trimethylsilyloxyethylene (**2c**) and 1-phenyl-1-trimethylsilyloxyethylene (**2d**) were also incorporated into the dioxolane product (entries 7–14) [61]. In the reaction of styrenes, quenching at rt led to a diastereomeric mixture of **3** and **3′** (entries 1 and 3). The diastereoselectivity was highly improved by quenching at a lower temperature (entries 2 and 4). The *trans*-configuration of the major product **3c** or **3d** was confirmed by the ^1^H-NMR NOESY experiment (SM). The stereochemical outcome may be explained by the steric fence owing to the aryl group during addition of the carbon nucleophile **2a** to the 1,3-dioxolan-2-yl cation, similar to the case of the meso cation generated from *cis*-octene (Scheme 1). Thus, the aryl group at the 4-position of the dioxolanyl cation was able to control the diastereoface selectivity in the nucleophilic trapping. However, the diastereomeric ratio decreased at a higher quenching temperature (entries 1 and 3). To gain an insight into the effect of quenching temperature on diastereoselectivity, the following control experiment was performed. A sample of a single diastereomer of **3d** with *trans*-configuration was treated with BF_3_·OEt_2_ in a dichloromethane solution at −40 °C and then warmed to rt. In the crude reaction mixture, isomerized *cis* dioxolane **3d′** was detected by ^1^H-NMR (**3d**(*trans*)/**3d′**(*cis*) = *ca*. 1/1). Thus, the lowering of the diastereomeric ratio may be due to a secondary reaction of the acetal product, *i.e.*, isomerization mediated by a Lewis acid.

**Table 2:** Stereoselective formation of dioxolane **3**^a^. 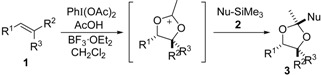

Entry	1	Nu-SiMe_3_	Product	Yield (%)
1 ^b^	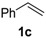	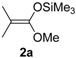	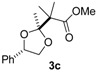	15 (1.2:1) ^c^
2				56 ^d^
3 ^b^	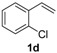	**2a**	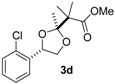	25 (1:1.2) ^c^
4				40 ^d^
5	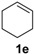	**2a**	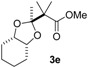	52 ^d^
6	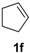	**2a**	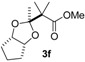	46 ^d^
7	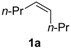	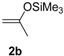	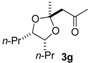	39 ^d^
8	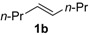	**2b**	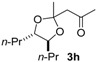	37
9	**1c**	**2b**	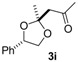	29 ^d^
10	**1d**	**2b**	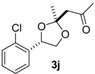	25 (8:1) ^c^
11	**1a**	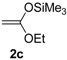	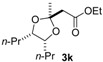	34 ^d^
12	**1b**	**2c**	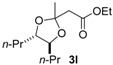	21
13	**1d**	**2c**	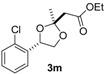	29 (7:1) ^c^
14	**1b**	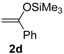	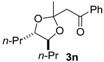	27

^a^ Reaction was initiated at −80 °C in the presence of **1** (0.32 mmol), PhI(OAc)_2_ (0.40 mmol), acetic acid (0.5 mmol) and BF_3_·OEt_2_ (0.8 mmol) in CH_2_Cl_2_ (4 mL). Then, **2** (1.5 mmol) was added at −40 °C and the reaction mixture was quenched at −30 °C by the addition of water; ^b^ The reaction was quenched after warming to rt; ^c^ The value in parentheses is the diastereomeric ratio of **3** and **3′** (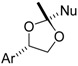); ^d^ Diastereomer was not detected by ^1^H-NMR.

Diastereoselectivity was also affected by the steric effect of the carbon nucleophile; β-unsubstituted silyl enol ethers **2b** and **2c** led to low diastereoselectivity in the reaction with **1d** (entries 10 and 13, respectively). In contrast, a single diastereomer was formed by the reaction of *cis* alkene (entries 7 and 11), even with compact β-unsubstituted nucleophiles. The diastereoselectivity in the reaction of styrenes was affected by the steric effect of the carbon nucleophile **2**. Thus, the stereocontrol by the aryl group in the 4-aryl-1,3-dioxolan-2-yl cation may be slightly reduced compared with the 4,5-dialkyl substituted dioxolanyl cation generated from *cis* alkene.

**Scheme 3:**
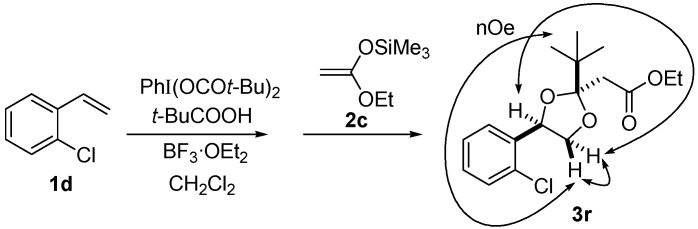
Reaction with pivalic acid leading to **3r**.

Next, a range of carboxylic acid nucleophiles was tested (Table 3). As a primitive examination, (diacetoxyiodo)benzene was used in the reaction of **1d** with propanoic acid; however, the protocol resulted in a mixture of **3d** and **3o**. Therefore, a set of hypervalent iodine and carboxylic acid compounds was employed as reagents for the reaction. The dioxolane product was obtained from benzoic acid (entry 4), as well as primary and secondary aliphatic carboxylic acids (entries 1 and 2, respectively). NOESY analysis indicated that they have *trans*-configuration, similar to the acetate-incorporated dioxolane **3d**. However, the corresponding dioxolane product was not obtained from the tertiary carboxylic acid (entry 3). In this case, 2-hydroxy-1-arylethyl pivalate **6** was produced as an ordinary oxidation product. The sterically bulky pivalic acid was incorporated into the dioxolane product **3r** when the compact nucleophile **2c** was used (Scheme 3). This reaction led to a 1:1 mixture of **3r** and **6**, and the dioxolane trapping product **3r** was isolated in 10% yield as a single diastereomer with *cis* configuration. The relative stereochemistry was confirmed by the nOe observed in the NOESY spectrum (SM), in accordance with stereoface differentiation during nucleophilic addition to the dioxolanyl cation intermediate, as discussed above.

**Table 3:** Scope of carboxylic acids ^a^. 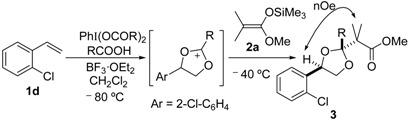

Entry	R	Yield (%)
1	Et	**3o**, 41
2	*i*-Pr	**3p**, 35
3	*t*-Bu	^b^
4	Ph	**3q**, 26

^a^ Reaction was initiated in the presence of **1d** (0.32 mmol), PhI(OCOR)_2_ (0.40 mmol), RCOOH (0.5 mmol) and BF_3_·OEt_2_ (0.8 mmol) in CH_2_Cl_2_ (4 mL). Then, **2a** (1.5 mmol) was added at −40 °C; ^b^ No dioxolane **3** was observed and 2-hydroxy-1-arylethyl pivalate **6** was obtained in 19% yield.

**Scheme 4:**
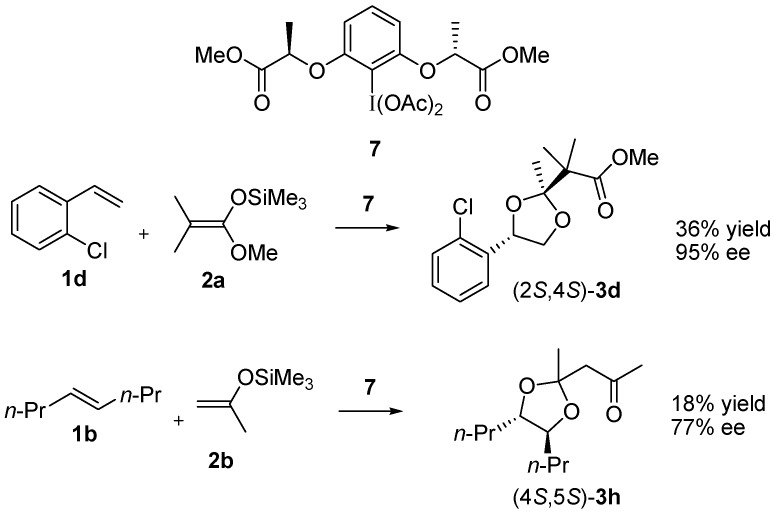
Enantioselective dioxolane formation.

On the basis of the reaction pathway discussed above, we examined enantioselective preparation of the dioxolane product using a chiral hypervalent iodine reagent, as shown in Scheme 4. The lactate-based chiral hypervalent iodine reagent **7** [62,63,64,65,66,67,68,69,70,71] has been previously used for enantioselective oxidation of styrenes and has led to high enantioselectivity; a Woodward reaction of **1d** with **7** gave the oxyacetylation product with 92% ee [57]. Comparable enantioselectivity was observed for the three-component assembly but the yield of **3d** was low (20%). The yield was slightly improved to 36% when trimethylsilyl acetate was added instead of acetic acid (Scheme 4). An aliphatic alkene **1b** was also examined for the enantioselective reaction, and enantioselectivity was slightly decreased in comparison with the styrene substrate. The ee value of the dioxolane **3h** (77% ee) was comparable with that of the acetoxy product **4b** (76% ee) obtained in a separate reaction without addition of the carbon nucleophile **2b**.

In conclusion, we achieved the stereoselective formation of 1,3-dioxolane compounds via a 1,3-dioxolan-2-yl cation intermediate, which is stereospecifically generated during the oxidation of alkenes with hypervalent iodine. Three components, alkene, carboxylic acid and carbon nucleophile, were stereoselectively assembled into the dioxolane product over the course of the reaction.

## 3. Experimental Section

### 3.1. General Information

Dichloromethane was purified by distillation over calcium hydride prior to use. Boron trifluoride diethyl etherate was distilled over calcium hydroxide, and kept under nitrogen. All commercial available reagents were used without further purification. (Diacyloxyiodo)benzenes were prepared according to the literature [24]. Reaction temperature was controlled using low temperature baths with magnetic stirrer PSL-1800 (EYELA, Tokyo, Japan) and PSL-1810 (EYELA). Proton and ^13^C-NMR spectra were measured on a JEOL ECA-600 spectrometer (JEOL, Tokyo, Japan) as solutions in CDCl_3_. Proton NMR spectra were recorded using the residual CHCl_3_ as an internal reference (7.24 ppm) and ^13^C-NMR using CDCl_3_ as an internal reference (77.00 ppm). For mass spectra measurements was used JEOL JMS-T100LC (JEOL). Optical rotations were measured on a Perkin-Elmer 241 polarimeter (Perkin-Elmer, Waltham, MA, USA). Gas chromatography was performed on Shimadzu GC-17A (Shimadzu, Kyoto, Japan) equipped with a chiral column (Chirasil-DEX-CB, 25 m × 0.25 mm × 0.25 µm film thickness, Agilent Technology, Santa Clara, CA, USA).

### 3.2. Typical Procedure for the Three-Component Assembly Reaction

(Diacetoxyiodo)benzene (130 mg, 0.40 mmol) and *cis*-4-octene **1a** (50 µL, 0.32 mmol) were dissolved in dichloromethane (4 mL) in the presence of acetic acid (30 µL, 0.5 mmol). The solution was cooled at −80 °C using a low temperature bath with magnetic stirrer (EYELA, PSL-1800). Boron trifluoride diethyl etherate (0.1 mL, 0.8 mmol) was added to the solution at −80 °C. The solution was warmed up to −40 °C over 1 h. Dimethylketene methyl trimethylsilyl acetal **2a** (0.3 mL, 1.5 mmol) was added to the solution at −40 °C and then the mixture was allowed to warm up to −30 °C over 1 h. The reaction mixture was quenched by the addition of water and extracted with dichloromethane. The organic phase was dried with MgSO_4_ and concentrated *in vacuo*. The crude mixture was then purified by column chromatography (SiO_2_, eluent: 10% ethyl acetate in hexane) to yield **3a** (54.2 mg, 0.20 mmol, 62% yield).

*Methyl 2-methyl-2-((2r,4R,5S)-2-methyl-4,5-dipropyl-1,3-dioxolan-2-yl)propanoate* (**3a**). ^1^H-NMR (600 MHz, CDCl_3_) δ 4.05 (m, 2H), 3.65 (s, 3H), 1.54–1.46 (m, 4H), 1.38 (s, 3H), 1.35–1.27 (m, 4H), 1.22 (s, 6H), 0.92 (t, *J* = 6.9 Hz, 6H); ^13^C-NMR (150 MHz, CDCl_3_) δ 176.2, 110.8, 79.5, 52.0, 51.9, 32.4, 25.0, 21.8, 19.6, 14.1; HRMS (ESI-TOF) *m*/*z*: [M + Na]^+^ Calcd for C_1__5_H_28_NaO_4_ 295.1885; Found 295.1882.

Methyl 2-methyl-2-((4R*,5R*)-2-methyl-4,5-dipropyl-1,3-dioxolan-2-yl)propanoate (**3b**). ^1^H-NMR (600 MHz, CDCl_3_) δ 3.64 (s, 3H), 3.62 (m, 1H), 3.45 (m, 1H), 1.53–1.30 (m, 8H), 1.33 (s, 3H), 1.21 (s, 3H), 1.20 (s, 3H), 0.92 (t, *J* = 6.9 Hz, 3H), 0.91 (t, *J* = 6.9 Hz, 3H); ^13^C-NMR (150 MHz, CDCl_3_) δ 176.0, 110.5, 82.1, 80.2, 51.8, 51.1, 35.6, 34.0, 22.3, 21.4, 21.1, 19.3, 19.2, 14.23, 14.16; HRMS (ESI-TOF) *m*/*z*: [M + Na]^+^ Calcd for C_1__5_H_28_NaO_4_ 295.1885; Found 295.1874.

Methyl 2-methyl-2-((2R*,4R*)-2-methyl-4-phenyl-1,3-dioxolan-2-yl)propanoate (**3c**). ^1^H-NMR (600 MHz, CDCl_3_) δ 7.36–7.26 (m, 5H), 4.96 (dd, *J* = 9.6, 6.2 Hz, 1H), 4.36 (dd, *J* = 8.9, 6.2 Hz, 1H), 3.70 (s, 3H), 3.67 (dd, *J* = 9.6, 8.9 Hz, 1H), 1.54 (s, 3H), 1.33 (s, 3H), 1.32 (s, 3H); ^13^C-NMR (150 MHz, CDCl_3_) δ 175.7, 138.4, 128.6, 128.1, 126.2, 112.8, 79.6, 72.1, 52.0, 51.3, 21.7, 21.6, 20.7; HRMS (ESI-TOF) *m*/*z*: [M + Na]^+^ Calcd for C_15_H_20_NaO_4_ 287.1259; Found 287.1256. Selected ^1^H-NMR data for the diastereomeric isomer **3c′**: ^1^H-NMR (600 MHz, CDCl_3_) δ 5.11 (dd, *J* = 8.9, 6.2 Hz, 1H), 4.28 (dd, *J* = 7.6, 6.2 Hz, 1H), 3.69 (s, 3H), 3.53 (dd, *J* = 8.9, 7.6 Hz, 1H).

Methyl 2-((2R*,4R*)-4-(2-chlorophenyl)-2-methyl-1,3-dioxolan-2-yl)-2-methylpropanoate (**3d**). ^1^H-NMR (600 MHz, CDCl_3_) δ 7.61 (d, *J* = 7.6 Hz, 1H), 7.31–7.26 (m, 2H), 7.21 (t, *J* = 7.6 Hz, 1H), 5.29 (dd, *J* = 9.6, 6.2 Hz, 1H), 4.64 (dd, *J* = 8.9, 6.2 Hz, 1H), 3.71 (s, 3H), 3.55 (dd, *J* = 9.6, 8.9 Hz, 1H), 1.51 (s, 3H), 1.34 (s, 3H), 1.33 (s, 3H); ^13^C-NMR (150 MHz, CDCl_3_) δ 175.7, 137.2, 131.6, 129.2, 128.8, 127.1, 126.7, 112.7, 76.5, 70.4, 52.1, 51.2, 21.5, 21.4, 20.9; HRMS (ESI-TOF) *m*/*z*: [M + Na]^+^ Calcd for C_15_H_19_^35^ClNaO_4_ 321.0870; Found 321.0873. Selected ^1^H-NMR data for the diastereomeric isomer **3d′**: ^1^H-NMR (600 MHz, CDCl_3_) δ 7.71 (d, *J* = 7.6 Hz, 1H), 5.41 (dd, *J* = 8.9, 6.2 Hz, 1H), 4.55 (dd, *J* = 7.6, 6.2 Hz, 1H), 3.69 (s, 3H), 3.39 (dd, *J* = 8.9, 7.6 Hz, 1H), 1.48 (s, 3H), 1.35 (s, 3H), 1.35 (s, 3H).

*Methyl 2-((2r,3aS,7aR)-hexahydro-2-methylbenzo[d][1,3]dioxol-2-yl)-2-methylpropanoate* (**3e**). ^1^H-NMR (600 MHz, CDCl_3_) δ 4.16 (t, *J* = 4.1 Hz, 2H), 3.66 (s, 3H), 1.80–1.69 (m, 4H), 1.53–1.47 (m, 2H), 1.50 (s, 3H), 1.25–1.20 (m, 2H), 1.23 (s, 6H); ^13^C-NMR (150 MHz, CDCl_3_) δ 176.2, 111.4, 75.3, 52.4, 52.0, 28.5, 24.7, 21.9, 20.5; HRMS (ESI-TOF) *m*/*z*: [M + Na]^+^ Calcd for C_1__3_H_22_NaO_4_ 265.1416; Found 265.1419.

*Methyl 2-((2r,3aS,6aR)-tetrahydro-2-methyl-3aH-cyclopenta[d][1,3]dioxol-2-yl)-2-methylpropanoate* (**3f**). ^1^H-NMR (600 MHz, CDCl_3_) δ 4.67 (t, *J* = 4.8 Hz, 2H), 3.66 (s, 3H), 1.86 (dd, *J* = 14.4, 6.9 Hz, 2H), 1.70 (m, 1H), 1.55 (m, 1H), 1.41 (s, 3H), 1.39 (m, 2H), 1.21 (s, 6H); ^13^C-NMR (150 MHz, CDCl_3_) δ 176.1, 114.5, 84.1, 52.4, 52.0, 34.2, 23.4, 22.6, 21.7; HRMS (ESI-TOF) *m*/*z*: [M + Na]^+^ Calcd for C_1__2_H_20_NaO_4_ 251.1259; Found 251.1256.

*1-((2r,4S,5R)-2-Methyl-4,5-dipropyl-1,3-dioxolan-2-yl)propan-2-one* (**3g**). ^1^H-NMR (600 MHz, CDCl_3_) δ 4.06 (m, 2H), 2.70 (s, 2H), 2.20 (s, 3H), 1.57–1.44 (m, 4H), 1.43 (s, 3H), 1.38–1.26 (m, 4H), 0.93 (t, *J* = 6.9 Hz, 6H); ^13^C-NMR (150 MHz, CDCl_3_) δ 206.6, 106.4, 78.2, 53.1, 31.8, 31.5, 27.0, 19.4, 14.1; HRMS (ESI-TOF) *m*/*z*: [M + Na]^+^ Calcd for C_1__3_H_24_NaO_3_ 251.1623; Found 251.1619.

1-((4R*,5R*)-2-Methyl-4,5-dipropyl-1,3-dioxolan-2-yl)propan-2-one (**3h**). ^1^H-NMR (600 MHz, CDCl_3_) δ 3.64–3.53 (m, 2H), 2.72 (s, 2H), 2.20 (s, 3H), 1.52–1.43 (m, 6H), 1.42–1.30 (m, 2H), 1.37 (s, 3H), 0.92 (t, *J* = 6.9 Hz, 6H); ^13^C-NMR (150 MHz, CDCl_3_) δ 206.5, 107.0, 81.2, 80.8, 54.1, 34.9, 34.7, 31.8, 26.1, 19.40, 19.37, 14.2, 14.1; HRMS (ESI-TOF) *m*/*z*: [M + Na]^+^ Calcd for C_1__3_H_24_NaO_3_ 251.1623; Found 251.1626.

1-((2R*,4R*)-2-Methyl-4-phenyl-1,3-dioxolan-2-yl)propan-2-one (**3i**). ^1^H-NMR (600 MHz, CDCl_3_) δ 7.38–7.27 (m, 5H), 5.07 (dd, *J* = 8.9, 6.2 Hz, 1H), 4.33 (dd, *J* = 8.9, 6.2 Hz, 1H), 3.72 (t, *J* = 8.9 Hz, 1H), 2.87 (s, 2H), 2.25 (s, 3H), 1.57 (s, 3H); ^13^C-NMR (150 MHz, CDCl_3_) δ 205.9, 138.3, 128.6, 128.2, 126.2, 108.9, 78.4, 71.7, 52.8, 31.8, 25.3; HRMS (ESI-TOF) *m*/*z*: [M + Na]^+^ Calcd for C_1__3_H_16_NaO_3_ 243.0997; Found 243.0999.

1-((2R*,4R*)-4-(2-Chlorophenyl)-2-methyl-1,3-dioxolan-2-yl)propan-2-one (**3j**). ^1^H-NMR (600 MHz, CDCl_3_) δ 7.60 (d, *J* = 7.6 Hz, 1H), 7.32 (d, *J* = 7.6 Hz, 1H), 7.29 (t, *J* = 7.6 Hz, 1H), 7.22 (t, *J* = 7.6 Hz, 1H), 5.41 (dd, *J* = 7.6, 6.9 Hz, 1H), 4.58 (dd, *J* = 8.2, 6.9 Hz, 1H), 3.65 (dd, *J* = 8.2, 7.6 Hz, 1H), 2.89 (s, 2H), 2.25 (s, 3H), 1.58 (s, 3H); ^13^C-NMR (150 MHz, CDCl_3_) δ 205.8, 137.2, 131.6, 129.2, 128.9, 127.1, 126.6, 108.9, 75.3, 70.3, 52.5, 31.7, 24.9; HRMS (ESI-TOF) *m*/*z*: [M + Na]^+^ Calcd for C_13_H_15_^35^ClNaO_3_ 277.0607; Found 277.0613. Selected ^1^H-NMR data for the diastereomeric isomer **3j′**: ^1^H-NMR (600 MHz, CDCl_3_) δ 7.58 (d, *J* = 7.6 Hz, 1H), 4.56 (dd, *J* = 8.2, 6.9 Hz, 1H), 3.61 (dd, *J* = 8.2, 7.6 Hz, 1H), 2.93 (s, 2H), 2.25 (s, 3H), 1.53 (s, 3H).

*Ethyl 2-((2r,4S,5R)-2-methyl-4,5-dipropyl-1,3-dioxolan-2-yl)acetate* (**3k**). ^1^H-NMR (600 MHz, CDCl_3_) δ 4.11 (q, *J* = 6.9 Hz, 2H), 4.06 (m, 2H), 2.62 (s, 2H), 1.53 (s, 3H), 1.52–1.40 (m, 4H), 1.37–1.25 (m, 4H), 1.23 (t, *J* = 6.9 Hz, 3H), 0.92 (t, *J* = 6.9 Hz, 6H); ^13^C-NMR (150 MHz, CDCl_3_) δ 169.7, 106.3, 78.1, 60.4, 44.6, 31.8, 27.0, 19.4, 14.2, 14.1; HRMS (ESI-TOF) *m*/*z*: [M + Na]^+^ Calcd for C_1__4_H_26_NaO_4_ 281.1729; Found 281.1716.

Ethyl 2-((4R*,5R*)-2-methyl-4,5-dipropyl-1,3-dioxolan-2-yl)acetate (**3l**). ^1^H-NMR (600 MHz, CDCl_3_) δ 4.12 (q, *J* = 6.9 Hz, 2H), 3.64 (td, *J* = 8.2, 4.1 Hz, 1H), 3.60 (td, *J* = 8.2, 4.1 Hz, 1H), 2.64 (d, *J* = 13.1 Hz, 1H), 2.62 (d, *J* = 13.1 Hz, 1H), 1.53–1.42 (m, 6H), 1.48 (s, 3H), 1.41–1.32 (m, 2H), 1.24 (t, *J* = 6.9 Hz, 3H), 0.92 (t, *J* = 6.9 Hz, 6H); ^13^C-NMR (150 MHz, CDCl_3_) δ 169.6, 106.8, 81.1, 81.0, 60.4, 45.7, 34.8, 34.7, 26.2, 19.30, 19.26, 14.19, 14.17, 14.1; HRMS (ESI-TOF) *m*/*z*: [M + Na]^+^ Calcd for C_1__4_H_26_NaO_4_ 281.1729; Found 281.1721.

Ethyl 2-((2R*,4R*)-4-(2-chlorophenyl)-2-methyl-1,3-dioxolan-2-yl)acetate (**3m**). ^1^H-NMR (600 MHz, CDCl_3_) δ 7.60 (d, *J* = 7.6 Hz, 1H), 7.31 (d, *J* = 7.6 Hz, 1H), 7.28 (t, *J* = 7.6 Hz, 1H), 7.21 (t, *J* = 7.6 Hz, 1H), 5.45 (dd, *J* = 7.6, 6.9 Hz, 1H), 4.57 (dd, *J* = 8.2, 6.9 Hz, 1H), 4.17 (q, *J* = 6.9 Hz, 2H), 3.66 (dd, *J* = 8.2, 7.6 Hz, 1H), 2.79 (s, 2H), 1.68 (s, 3H), 1.27 (t, *J* = 6.9 Hz, 3H); ^13^C-NMR (150 MHz, CDCl_3_) δ 169.3, 137.4, 131.6, 129.2, 128.8, 127.0, 126.7, 108.7, 75.3, 70.6, 60.7, 44.2, 25.0, 14.2; HRMS (ESI-TOF) *m*/*z*: [M + Na]^+^ Calcd for C_14_H_17_^35^ClNaO_4_ 307.0713; Found 307.0711. Selected ^1^H-NMR data for the diastereomeric isomer **3m′**: ^1^H-NMR (600 MHz, CDCl_3_) δ 7.69 (d, *J* = 7.6 Hz, 1H), 5.42 (dd, *J* = 8.2, 6.9Hz, 1H), 4.17 (q, *J* = 6.9 Hz, 2H), 3.59 (t, *J* = 8.2 Hz, 1H), 2.82 (s, 2H), 1.62 (s, 3H).

2-((4R*,5R*)-2-Methyl-4,5-dipropyl-1,3-dioxolan-2-yl)-1-phenylethanone (**3n**). ^1^H-NMR (600 MHz, CDCl_3_) δ 7.97 (d, *J* = 7.6 Hz, 2H), 7.52 (t, *J* = 7.6 Hz, 1H), 7.42 (t, *J* = 7.6 Hz, 2H), 3.56 (td, *J* = 7.6, 3.4 Hz, 1H), 3.42 (td, *J* = 7.6, 3.4 Hz, 1H), 3.30 (d, *J* = 13.7 Hz, 1H), 3.27 (d, *J* = 13.7 Hz, 1H), 1.48 (s, 3H), 1.45–1.18 (m, 8H), 0.85 (t, *J* = 6.9 Hz, 3H), 0.84 (t, *J* = 6.9 Hz, 3H); ^13^C-NMR (150 MHz, CDCl_3_) δ 197.6, 138.0, 132.8, 129.0, 128.2, 107.6, 81.2, 80.6, 48.9, 34.6, 34.3, 26.8, 19.3, 19.2, 14.11, 14.08; HRMS (ESI-TOF) *m*/*z*: [M + Na]^+^ Calcd for C_1__8_H_26_NaO_3_ 313.1780; Found 313.1777.

Methyl 2-((2R*,4R*)-4-(2-chlorophenyl)-2-ethyl-1,3-dioxolan-2-yl)-2-methylpropanoate (**3o**). ^1^H-NMR (600 MHz, CDCl_3_) δ 7.73 (d, *J* = 7.6 Hz, 1H), 7.31 (d, *J* = 7.6 Hz, 1H), 7.29 (t, *J* = 7.6 Hz, 1H), 7.21 (t, *J* = 7.6 Hz, 1H), 5.37 (dd, *J* = 9.6, 6.9 Hz, 1H), 4.70 (dd, *J* = 8.2, 6.9 Hz, 1H), 3.70 (s, 3H), 3.58 (dd, *J* = 9.6, 8.2 Hz, 1H), 1.96 (dq, *J* = 15.1, 7.6 Hz, 1H), 1.94 (dq, *J* = 15.1, 7.6 Hz, 1H), 1.33 (s, 3H), 1.32 (s, 3H), 0.92 (t, *J* = 7.6 Hz, 3H); ^13^C-NMR (150 MHz, CDCl_3_) δ 176.0, 136.8, 131.5, 129.2, 128.7, 127.0, 126.4, 114.5, 76.5, 72.1, 52.2, 52.1, 28.4, 21.9, 21.6, 8.1; HRMS (ESI-TOF) *m*/*z*: [M + Na]^+^ Calcd for C_1__6_H_21_^35^ClNaO_4_ 335.1026; Found 335.1014.

Methyl 2-((2R*,4R*)-4-(2-chlorophenyl)-2-isopropyl-1,3-dioxolan-2-yl)-2-methylpropanoate (**3p**). ^1^H-NMR (600 MHz, CDCl_3_) δ 7.75 (d, *J* = 7.6 Hz, 1H), 7.30 (t, *J* = 7.6 Hz, 1H), 7.28 (d, *J* = 7.6 Hz, 1H), 7.21 (t, *J* = 7.6 Hz, 1H), 5.44 (dd, *J* = 9.6, 6.9 Hz, 1H), 4.70 (t, *J* = 6.9 Hz, 1H), 3.69 (s, 3H), 3.53 (dd, *J* = 9.6, 6.9 Hz, 1H), 2.38 (sept, *J* = 6.9 Hz, 1H), 1.36 (s, 3H), 1.34 (s, 3H), 1.04 (d, *J* = 6.9 Hz, 3H), 0.94 (d, *J* = 6.9 Hz, 3H); ^13^C-NMR (150 MHz, CDCl_3_) δ 176.3, 137.0, 131.4, 129.3, 128.7, 127.0, 126.5, 115.2, 76.7, 71.6, 52.4, 52.0, 34.9, 22.6, 22.5, 19.1, 18.3; HRMS (ESI-TOF) *m*/*z*: [M + Na]^+^ Calcd for C_1__7_H_23_^35^ClNaO_4_ 349.1183; Found 349.1186.

Methyl 2-((2R*,4R*)-4-(2-chlorophenyl)-2-phenyl-1,3-dioxolan-2-yl)-2-methylpropanoate (**3q**). ^1^H-NMR (600 MHz, CDCl_3_) δ 7.54 (d, *J* = 7.6 Hz, 2H), 7.41 (t, *J* = 7.6 Hz, 1H), 7.35 (t, *J* = 7.6 Hz, 2H), 7.30 (t, *J* = 7.6 Hz, 1H), 7.25 (t, *J* = 7.6 Hz, 1H), 7.14 (d, *J* = 7.6 Hz, 1H), 7.13 (d, *J* = 7.6 Hz, 1H), 5.46 (dd, *J* = 9.6, 5.5 Hz, 1H), 4.61 (dd, *J* = 8.2, 5.5 Hz, 1H), 3.65 (s, 3H), 3.24 (dd, *J* = 9.6, 8.2 Hz, 1H), 1.33 (s, 3H), 1.27 (s, 3H); ^13^C-NMR (150 MHz, CDCl_3_) δ 175.0, 140.1, 135.9, 131.9, 129.1, 128.8, 128.2, 127.7, 127.5, 127.3, 127.0, 112.4, 77.6, 70.6, 51.9, 51.5, 21.5, 21.2; HRMS (ESI-TOF) *m*/*z*: [M + Na]^+^ Calcd for C_20_H_21_^35^ClNaO_4_ 383.1026; Found 383.1013.

Ethyl 2-((2R*,4S*)-2-tert-butyl-4-(2-chlorophenyl)-1,3-dioxolan-2-yl)acetate (**3r**). ^1^H-NMR (600 MHz, CDCl_3_) δ 7.70 (d, *J* = 7.6 Hz, 1H), 7.31 (d, *J* = 7.6 Hz, 1H), 7.28 (t, *J* = 7.6 Hz, 1H), 7.21 (t, *J* = 7.6 Hz, 1H), 5.52 (dd, *J* = 9.6, 6.9 Hz, 1H), 4.66 (t, *J* = 6.9 Hz, 1H), 4.19 (dq, *J* = 9.6, 6.9 Hz, 1H), 4.16 (dq, *J* = 9.6, 6.9 Hz, 1H), 3.47 (dd, *J* = 9.6, 6.9 Hz, 1H), 2.88 (d, *J* = 13.7 Hz, 1H), 2.82 (d, *J* = 13.7 Hz, 1H), 1.29 (t, *J* = 6.9 Hz, 3H), 1.06 (s, 9H); ^13^C-NMR (150 MHz, CDCl_3_) δ 170.7, 136.6, 131.5, 129.3, 128.7, 127.0, 126.4, 114.2, 75.8, 71.6, 60.7, 40.8, 40.6, 25.4, 14.2; HRMS (ESI-TOF) *m*/*z*: [M + Na]^+^ Calcd for C_17_H_23_^35^ClNaO_4_ 349.1183; Found 349.1182.

*1-(2-Chlorophenyl)-2-hydroxyethyl pivalate* (**6**). ^1^H-NMR (600 MHz, CDCl_3_) δ 7.59 (d, *J* = 7.6 Hz, 1H), 7.33 (d, *J* = 7.6 Hz, 1H), 7.29 (t, *J* = 7.6 Hz, 1H), 7.23 (t, *J* = 7.6 Hz, 1H), 5.36 (dd, *J* = 7.6, 2.7 Hz, 1H), 4.35 (dd, *J* = 11.7, 2.7 Hz, 1H), 4.20 (dd, *J* = 11.7, 7.6 Hz, 1H), 1.18 (s, 9H); ^13^C-NMR (150 MHz, CDCl_3_) δ 179.1, 137.3, 132.0, 129.4, 129.1, 127.8, 127.0, 69.6, 67.8, 38.9, 27.2; HRMS (ESI-TOF) *m*/*z*: [M + Na]^+^ Calcd for C_13_H_17_^35^ClNaO_3_ 279.0764; Found 279.0761.

### 3.3. Isomerization of ***3d*** Mediated by a Lewis Acid

Boron trifluoride diethyl etherate (0.1 mL, 0.8 mmol) was added to a dichloromethane solution (4 mL) containing **3d** (36.2 mg, 0.12 mmol) at −40 °C. The solution was gradually warmed up to 0 °C over 1 h. The reaction mixture was quenched by the addition of water and extracted with dichloromethane. The organic phase was dried with Na_2_SO_4_ and concentrated *in vacuo*. The crude mixture contained **3d** and the diastereomer **3d′** in a 45 to 55 ratio, in addition to decomposed products. The crude mixture was purified by column chromatography (SiO_2_, eluent: 10% ethyl acetate in hexane) to give a diastereomeric mixture (**3d**/**3d′** = 48/52, 8.4 mg, 23% yield).

### 3.4. Enantioselective Reaction of ***1d*** with ***7*** and ***2a***

The lactate-based hypervalent iodine(III) reagent **7** (260 mg, 0.50 mmol) and 2-chlorostyrene **1d** (55 μL, 0.43 mmol) were dissolved in dichloromethane (4 mL) in the presence of trimethylsilyl acetate (30 μL, 0.20 mmol). The solution was cooled at −80 °C using a low temperature bath with magnetic stirrer (EYELA, PSL-1800). Boron trifluoride diethyl etherate (0.1 mL, 0.8 mmol) was added to the solution at −80 °C and the mixture was stirred for 1 h at −80 °C. Dimethylketene methyl trimethylsilyl acetal **2a** (0.3 mL, 1.5 mmol) was added to the solution at −80 °C and the mixture was stirred for 30 min at −80 °C. The reaction mixture was quenched by the addition of water and extracted with dichloromethane. The organic phase was dried with Na_2_SO_4_ and concentrated *in vacuo*. The crude mixture was purified by column chromatography (SiO_2_, eluent: 10% ethyl acetate in hexane) to give **3d** (46.6 mg, 0.16 mmol, 36% yield). Selected data for (2*S*,4*S*)-**3d**: ${\text{[α]}}_{\text{D}}^{20}$ +33.5 (*c* 0.83 in CH_2_Cl_2_). Ee of **3d** was determined to be 95% by GC on a chiral stationary phase (DEX-CB, i.d. 0.25 mm × 25 m). Retention times of (2*R*,4*R*)-**3d** and (2*S*,4*S*)-**3d** were 30.1 and 31.1 min, respectively, when the column temperature was maintained at 160 °C. The absolute stereochemistry was based on the analogy of the enantioselective Woodward reaction of **1d** [57].

### 3.5. Enantioselective Reaction of ***1b*** with ***7*** and ***2b***

Boron trifluoride diethyl etherate (0.1 mL, 0.8 mmol) was added to a dichloromethane solution (4 mL) containing **7** (260 mg, 0.50 mmol), **1b** (68 μL, 0.43 mmol) and trimethylsilyl acetate (30 μL, 0.20 mmol) at −80 °C. The solution was warmed to −40 °C over 1 h and **2b** (0.3 mL, 1.8 mmol) was subsequently added to the solution at −40 °C. The mixture was warmed to −30 °C over 1 h and quenched by the addition of water. Extracts with dichloromethane were dried with Na_2_SO_4_ and concentrated *in vacuo*. The crude mixture was purified by column chromatography (SiO_2_, eluent: 10% ethyl acetate in hexane) to give **3h** (17.9 mg, 0.078 mmol, 18% yield). Selected data for (4*S*,5*S*)-**3h**: ${\text{[α]}}_{\text{D}}^{20}$ −18.9 (*c* 0.36 in CH_2_Cl_2_). Ee of **3h** was determined to be 77% by GC on a chiral stationary phase (DEX-CB, i.d. 0.25 mm × 25 m). Retention times of (4*R*,5*R*)-**3h** and (4*S*,5*S*)-**3h** were 39.6 and 40.2 min, respectively, when the column temperature was maintained at 100 °C. The absolute stereochemistry was based on the analogy of the enantioselective Woodward reaction of **1b**, which was described below.

(4R*,5S*)-5-Hydroxyoctan-4-yl acetate **4a**: Boron trifluoride diethyl etherate (0.1 mL, 0.8 mmol) was added to a dichloromethane solution (4 mL) containing PhI(OAc)_2_ (130 mg, 0.40 mmol), **1a** (50 μL, 0.32 mmol), acetic acid (0.2 mL, 3.5 mmol) and trimethylsilyl acetate (0.2 mL, 1.3 mmol) at −80 °C. The solution was gradually warmed to rt and stirred overnight. The reaction mixture was quenched by the addition of water and extracted with dichloromethane. The organic phase was dried with MgSO_4_ and concentrated *in vacuo*. The crude mixture was purified by column chromatography (SiO_2_, eluent: 20% ethyl acetate in hexane) to give **4a** (51.8 mg, 0.275 mmol, 86% yield). ^1^H-NMR (600 MHz, CDCl_3_) δ 4.86 (dt, *J* = 10.3, 3.4 Hz, 1H), 3.68 (m, 1H), 2.07 (s, 3H), 1.75–1.23 (m, 9H), 0.92 (t, *J* = 6.9 Hz, 3H), 0.90 (t, *J* = 6.9 Hz, 3H); ^13^C-NMR (150 MHz, CDCl_3_) δ 171.3, 77.5, 73.0, 34.3, 30.8, 21.2, 19.1, 18.9, 14.0, 13.9; HRMS (ESI-TOF) *m*/*z*: [M + Na]^+^ Calcd for C_10_H_20_NaO_3_ 211.1310; Found 211.1309.

Acetylation of **4a** with acetic anhydride gave *meso*-4,5-diacetoxyoctane (**5a**) in 87% yield. Selected data for **5a**: ^1^H-NMR (600 MHz, CDCl_3_) δ 4.98 (d, *J* = 11.0 Hz, 2H), 2.02 (s, 6H), 1.54 (m, 2H), 1.46 (m, 2H), 1.36 (m, 2H), 1.25 (m, 2H), 0.89 (t, *J* = 7.6 Hz, 6H); ^13^C-NMR (150 MHz, CDCl_3_) δ 170.7, 74.0, 31.3, 21.0, 18.8, 13.8. The NMR data agreed well with the reported values [72].

(4*S*,5*S*)-5-Hydroxyoctan-4-yl acetate (4*S*,5*S*)-**4b**: A Woodward reaction of *trans*-4-octene (**1b**) was performed according to similar procedures described above and gave a racemic sample of **4b** in 77% yield. ^1^H-NMR (600 MHz, CDCl_3_) δ 4.82 (dt, *J* = 7.6, 4.2 Hz, 1H), 3.57 (m, 1H), 2.07 (s, 3H), 1.63–1.27 (m, 8H), 0.90 (t, *J* = 6.9 Hz, 3H); ^13^C-NMR (150 MHz, CDCl_3_) δ 171.0, 76.4, 72.3, 35.9, 32.8, 21.1, 18.8, 18.7, 13.99, 13.95. The NMR data agreed well with the reported values [72]. HRMS (ESI-TOF) *m*/*z*: [M + Na]^+^ Calcd for C_10_H_20_NaO_3_ 211.1310; Found 211.1303.

Enantioselective reaction of **1b** (0.32 mmol) was performed using **7** and gave optically active **4b** in 53% yield (0.17 mmol). ${\text{[α]}}_{\text{D}}^{20}$ −22.2 (*c* 0.90 in CHCl_3_). Ee of the product was determined to be 76% ee of (4*S*,5*S*)-**4b** by GC on a chiral stationary phase (DEX-CB, i.d. 0.25 mm × 25 m). Retention times of (4*S*,5*S*)-**4b** and (4*R*,5*R*)-**4b** were 7.8 and 8.2 min, respectively, when the column temperature was maintained at 130 °C.

Acetylation of (4*S*,5*S*)-**4b** with acetic anhydride gave the corresponding diacetoxy compound (4*S*,5*S*)-**5b** in 87% yield. Selected data for (4*S*,5*S*)-**5b**: ^1^H-NMR (600 MHz, CDCl_3_) δ 4.99 (m, 2H), 2.06 (s, 6H), 1.53–1.24 (m, 8H), 0.88 (t, *J* = 6.9 Hz, 6H); ^13^C-NMR (150 MHz, CDCl_3_) δ 170.7, 73.7, 32.9, 20.9, 18.5, 13.9. The NMR data agreed well with the reported values [73]. ${\text{[α]}}_{\text{D}}^{20}$ −42.1 (*c* 0.96 in CHCl_3_). Ee of the product was determined to be 76% ee of (4*S*,5*S*)-**5b** by GC on a chiral stationary phase (DEX-CB, i.d. 0.25 mm × 25 m). Retention times of (4*S*,5*S*)-**5b** and (4*R*,5*R*)-**5b** were 7.7 and 7.9 min, respectively, when the column temperature was maintained at 130 °C.

The sample of (4*S*,5*S*)-**5b** was hydrolyzed under basic conditions and octane-4,5-diol was obtained. Optical rotation of the diol obtained (${\text{[α]}}_{\text{D}}^{20}$ −33 (*c* 0.54 in EtOH)) indicates that the sample has (4*S*,5*S*)-configuration; reported value for (4*R*,5*R*)-octane-4,5-diol: ${\text{[α]}}_{\text{D}}^{27}$ +44.4 (*c* 0.12 in EtOH) [74].

## Supplementary Materials

Supplementary materials can be accessed at: http://www.mdpi.com/1420-3049/20/09/17041/s1.
