# Supplementary material for: Stereoselective Formation of Substituted 1,3-Dioxolanes through a Three-Component Assembly during the Oxidation of Alkenes with Hypervalent Iodine(III)
Source: Molecules. 2015 Sep 17;20(9):17041–57. doi: 10.3390/molecules200917041 (PMC6332268; doi:10.3390/molecules200917041)
Supplement: Supplementary file 1 [file molecules-20-17041-s001.pdf]

Supplementary Materials

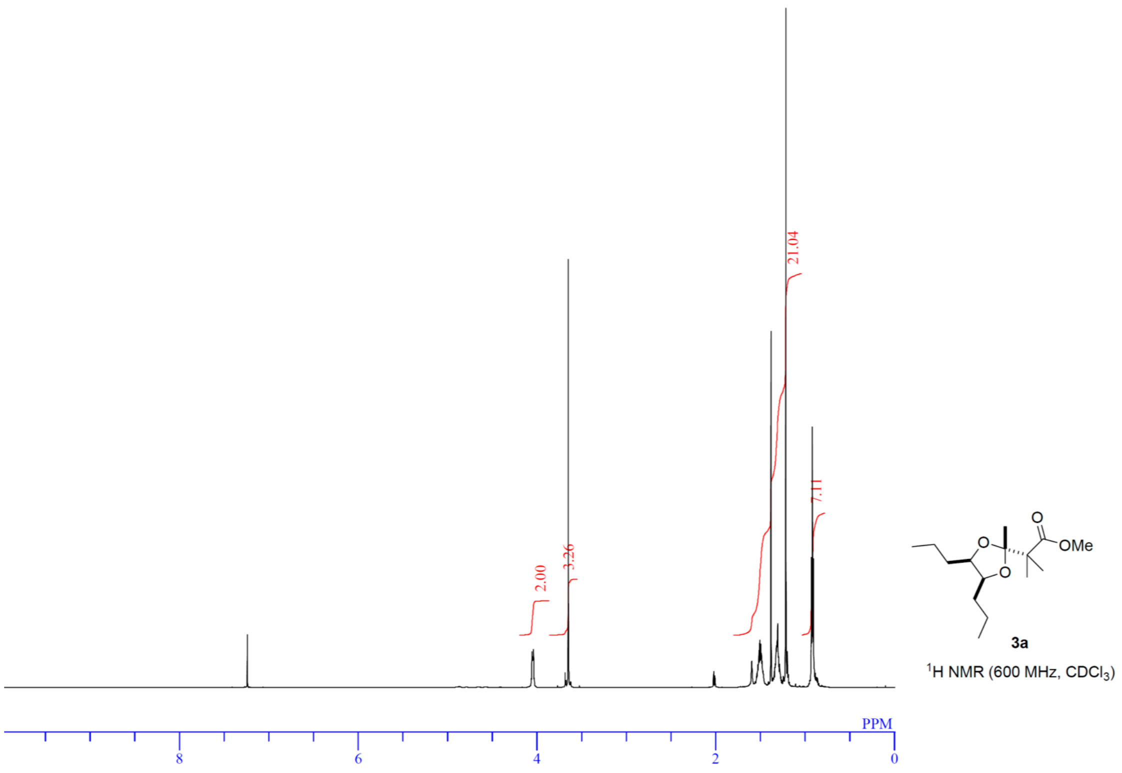

Figure S1. Proton NMR of **3a**.

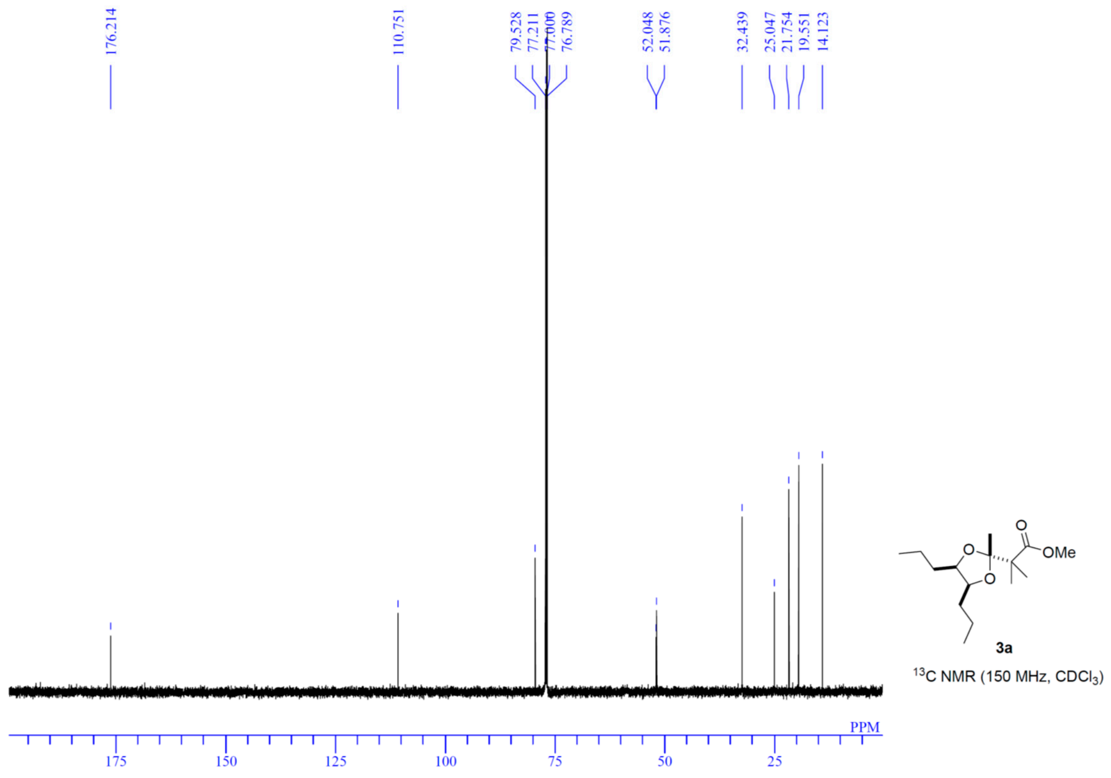

Figure S2. Carbon-13 NMR of **3a**.

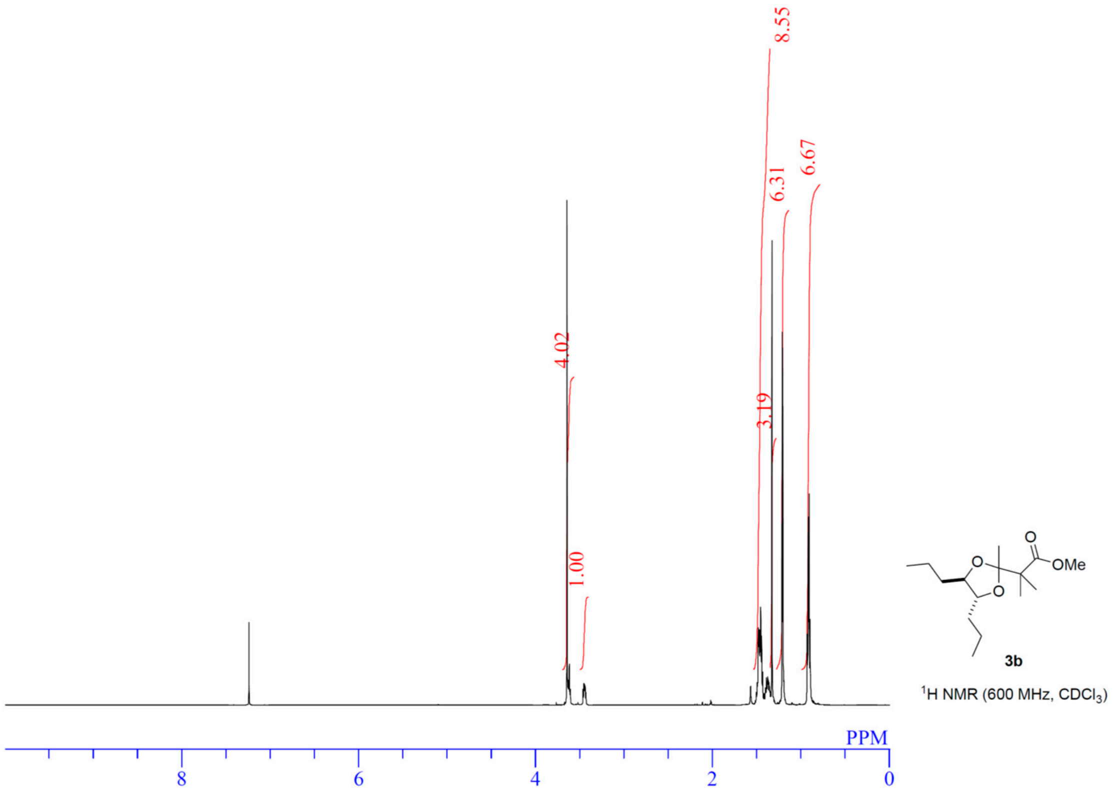

Figure S3. Proton NMR of **3b**.

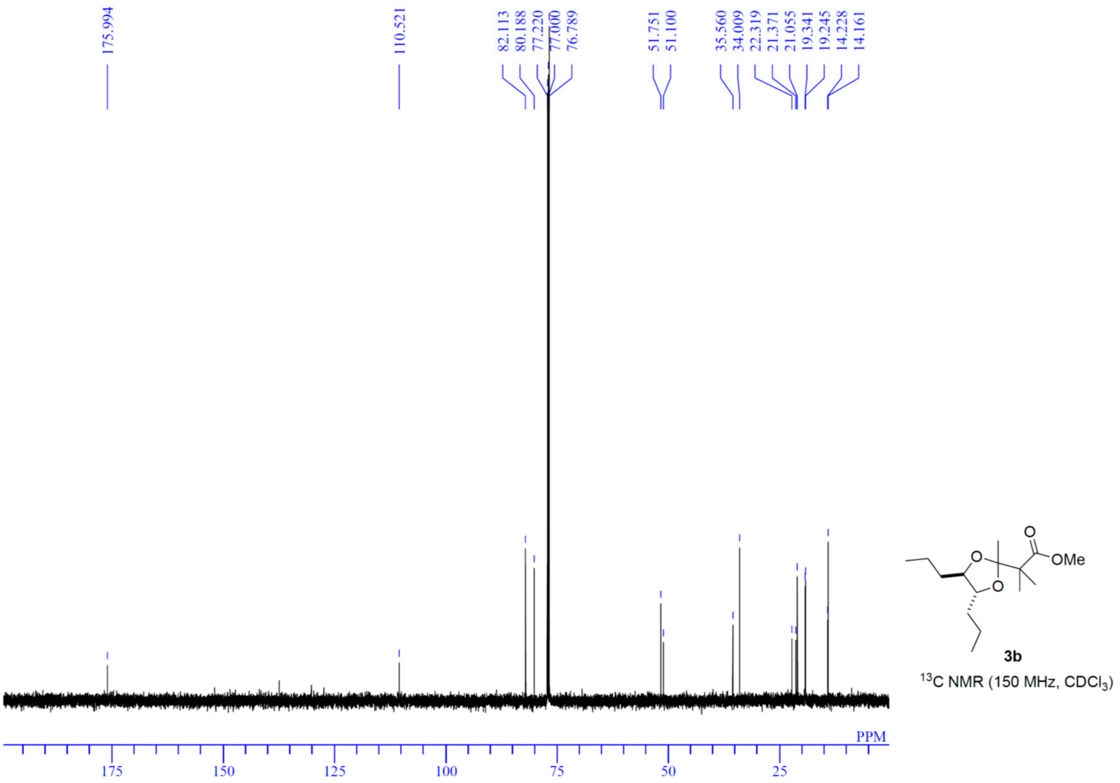

Figure S4. Carbon-13 NMR of **3b**.

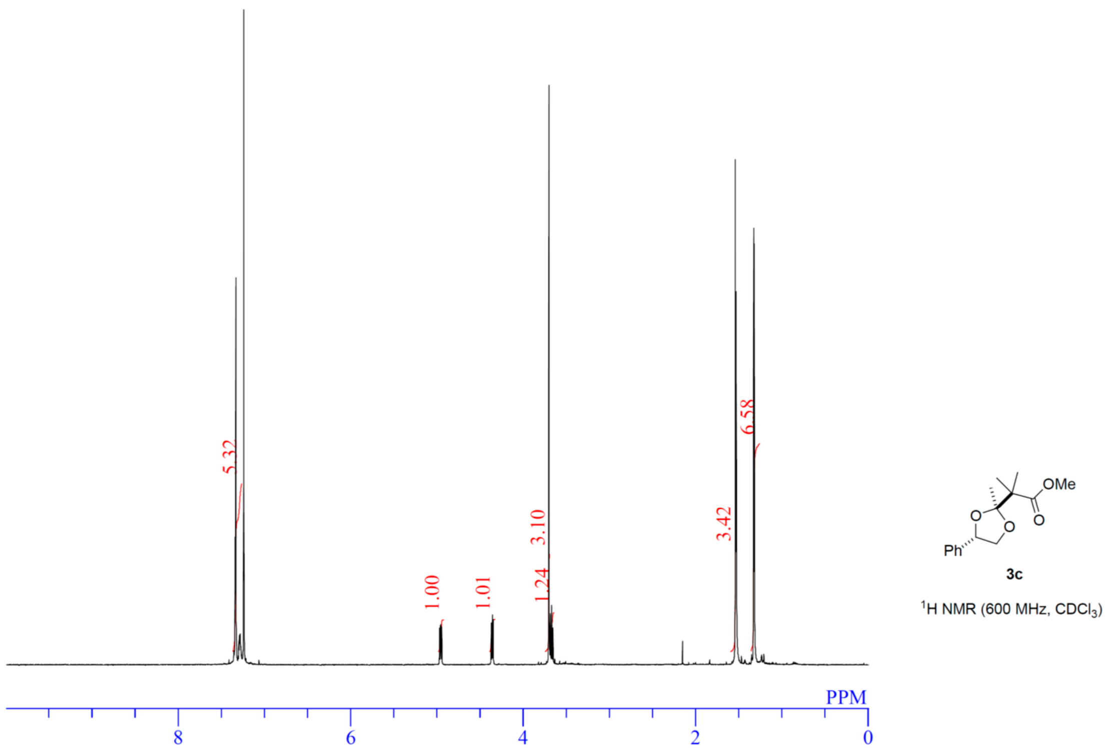

Figure S5. Proton NMR of **3c**.

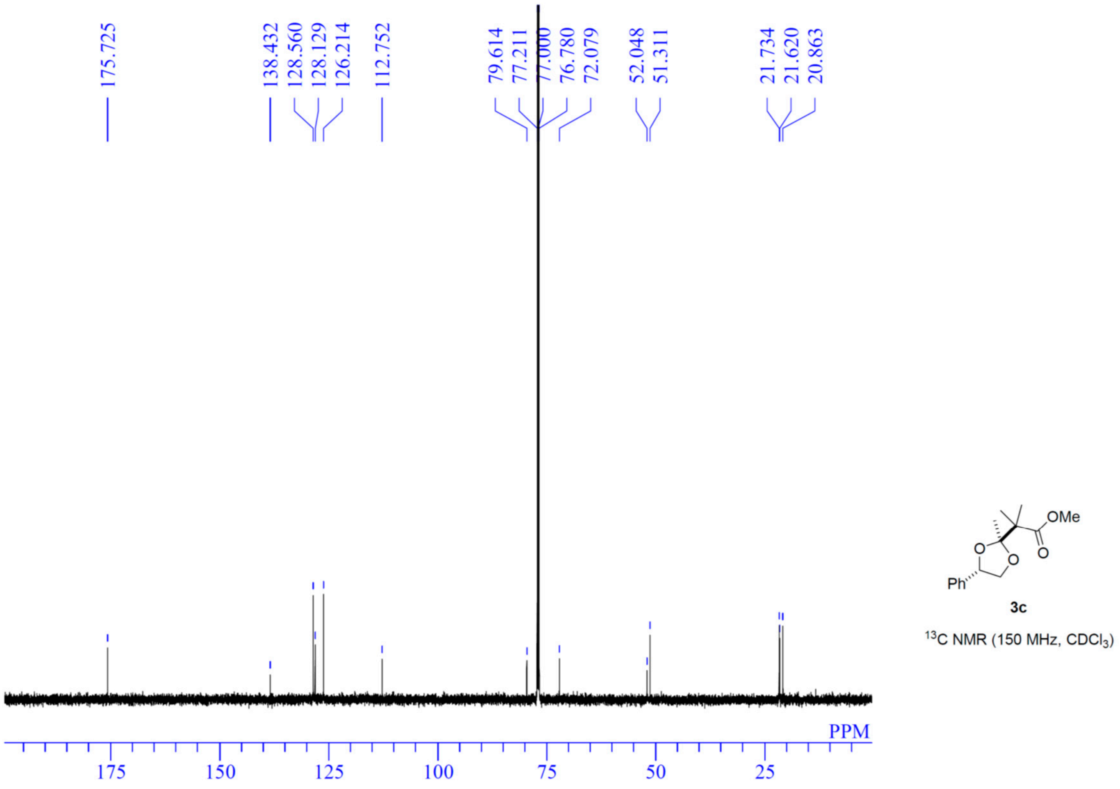

Figure S6. Carbon-13 NMR of **3c**.

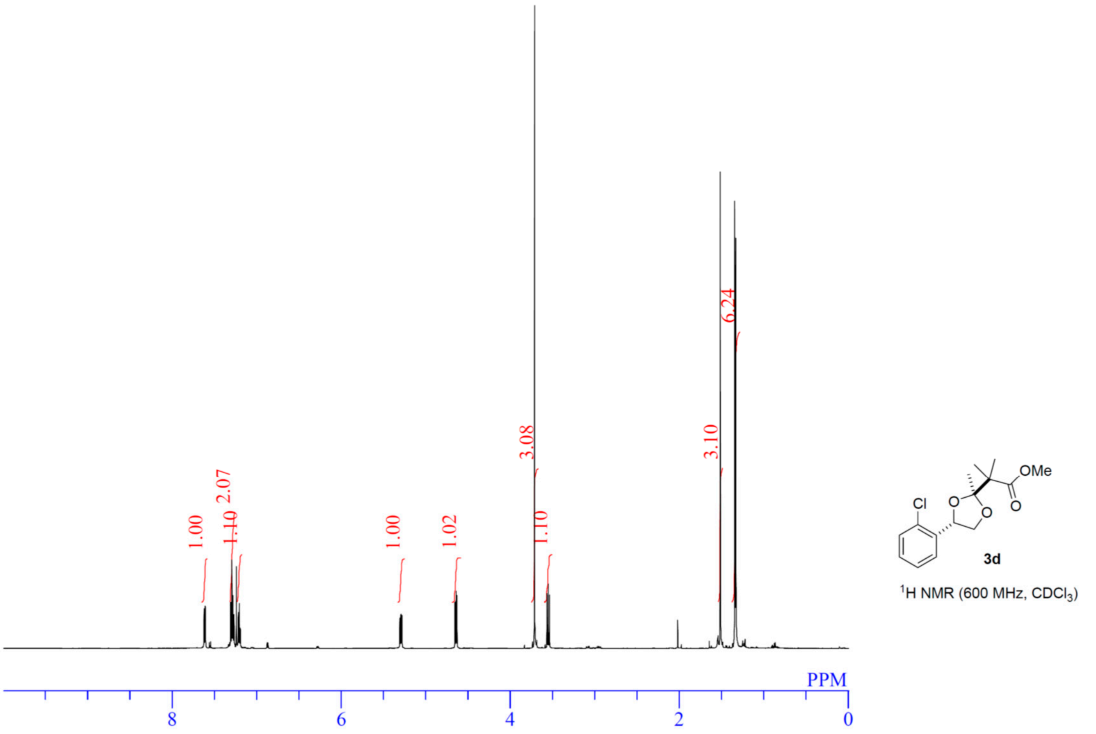

Figure S7. Proton NMR of **3d**.

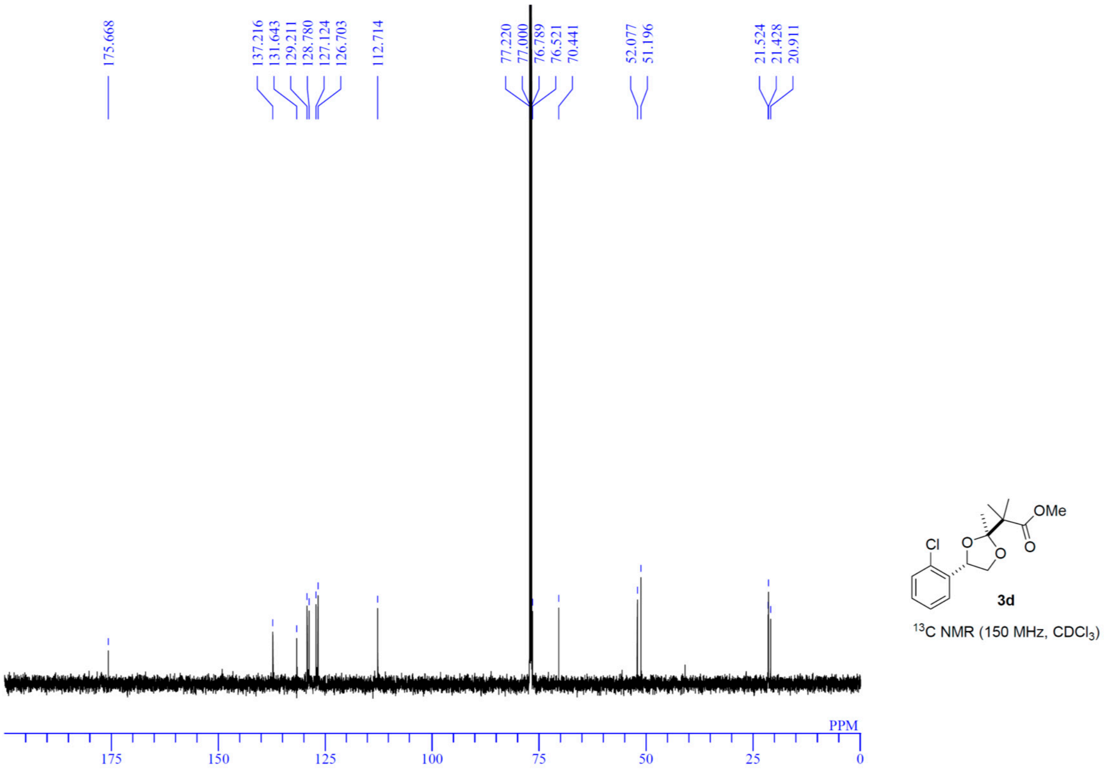

Figure S8. Carbon-13 NMR of **3d**.

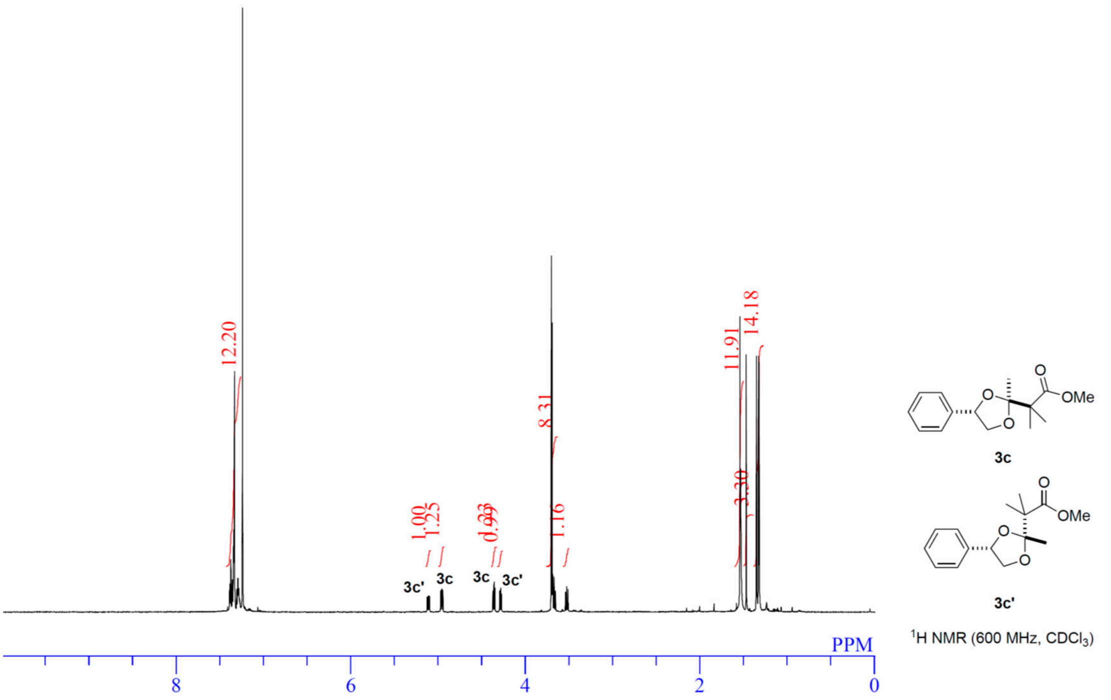

Figure S9. Proton NMR of **3c** and **3c'**.

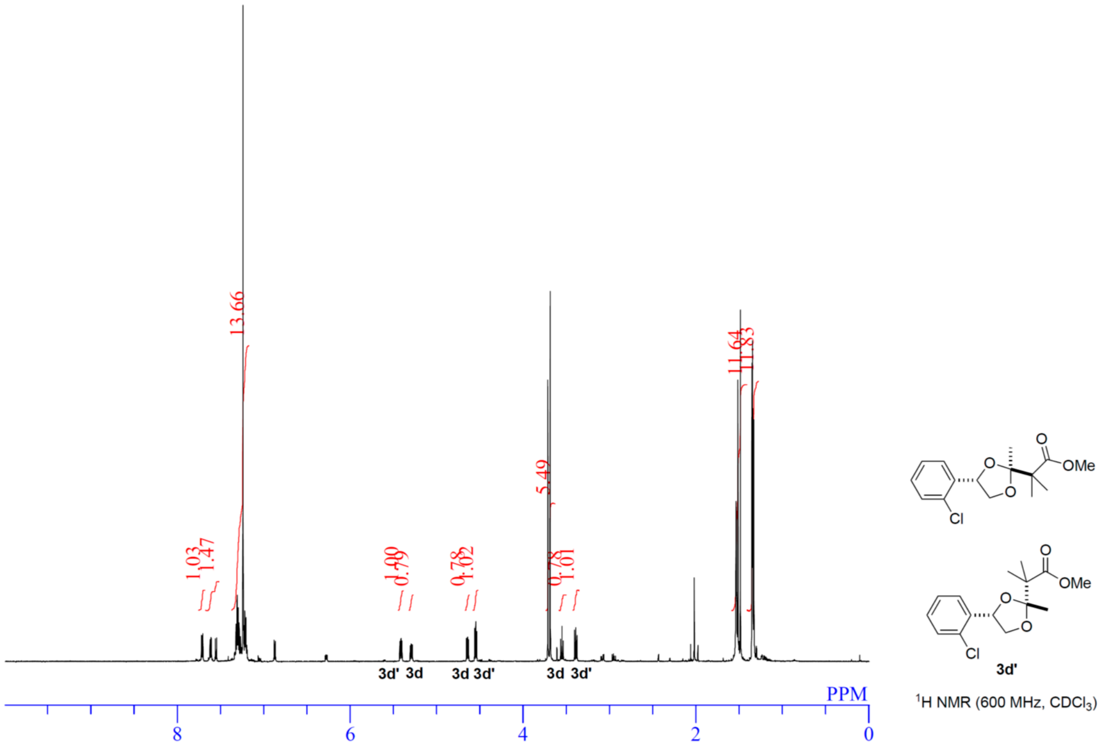

Figure S10. Proton NMR of **3d** and 3**d'**.

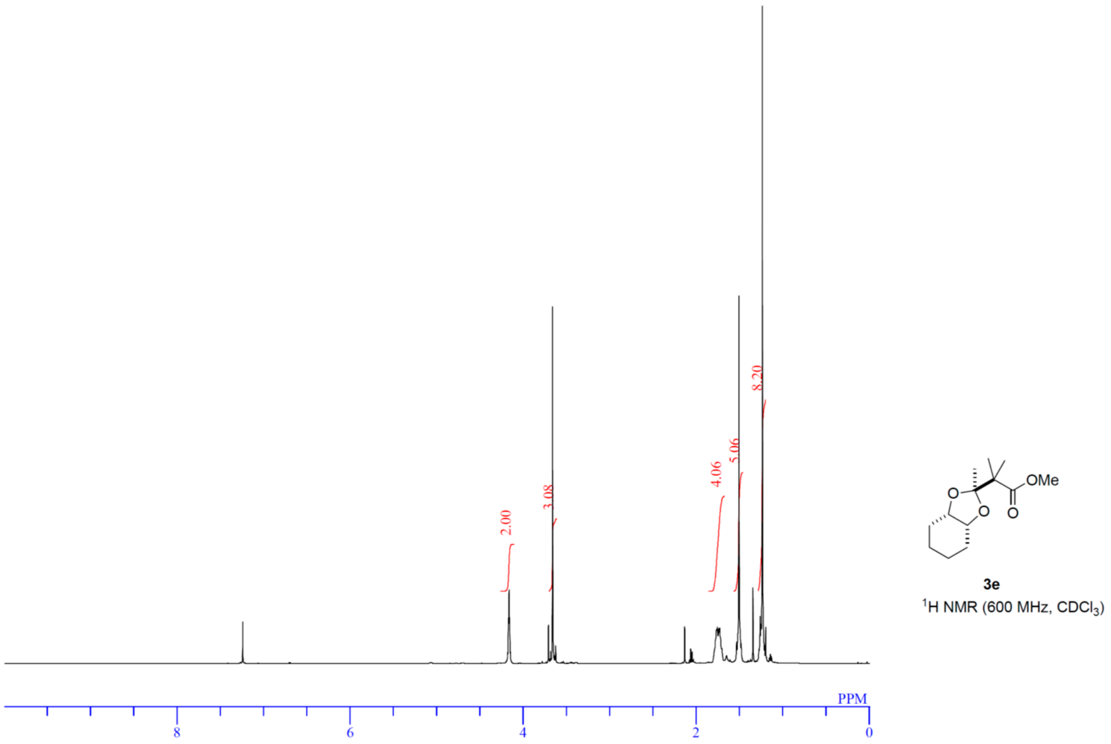

Figure S11. Proton NMR of **3e**.

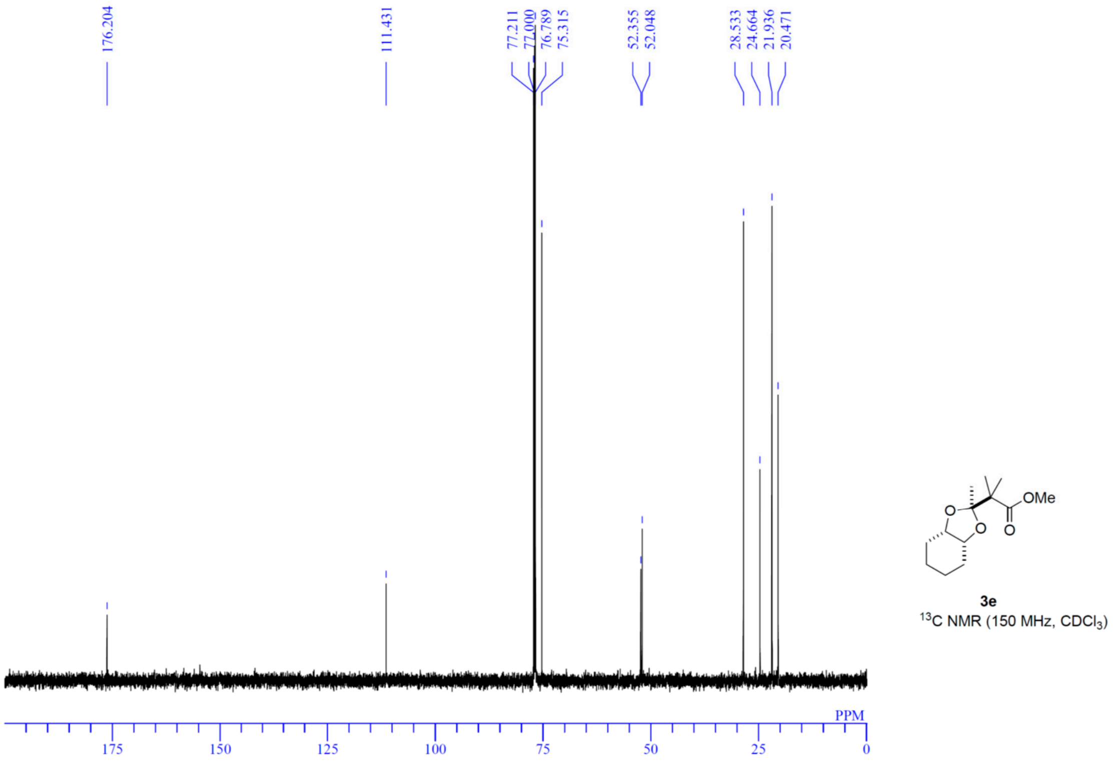

Figure S12. Carbon-13 NMR of **3e**.

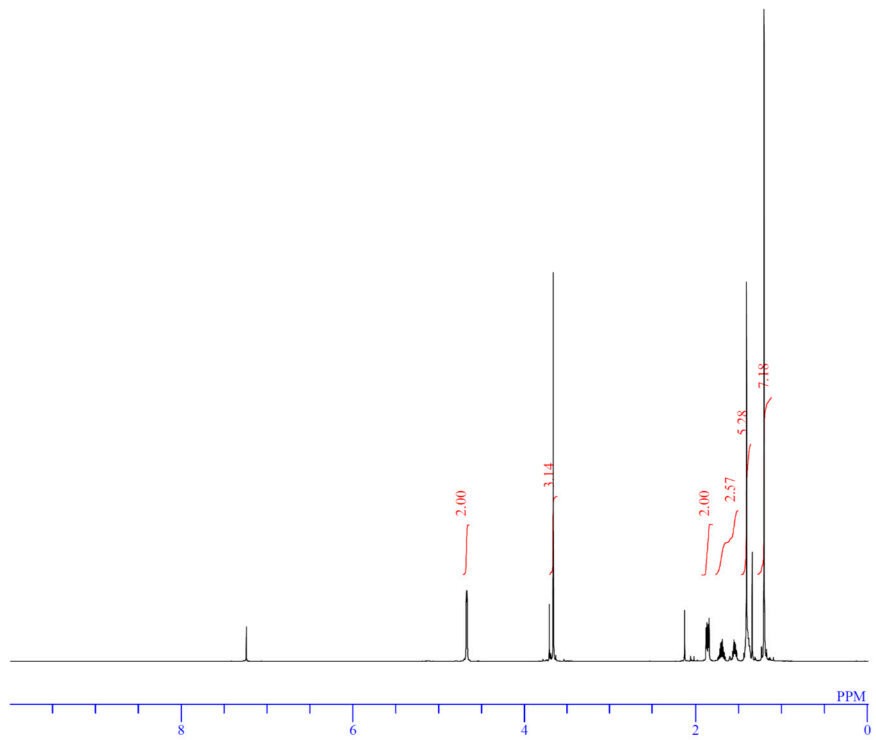

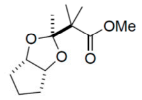

3f
^{1}H NMR (600 MHz, $CDCl_3$)

Figure S13. Proton NMR of **3f**.

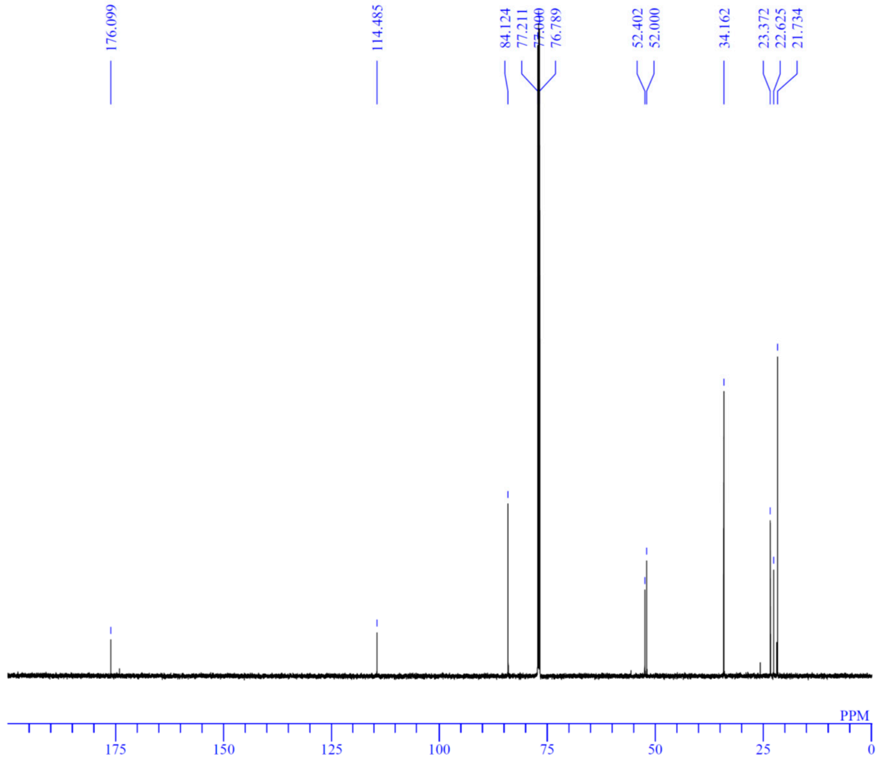

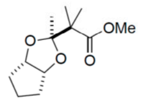

3f
^{13}C NMR (150 MHz, $CDCl_3$)

Figure S14. Carbon-13 NMR of **3f**.

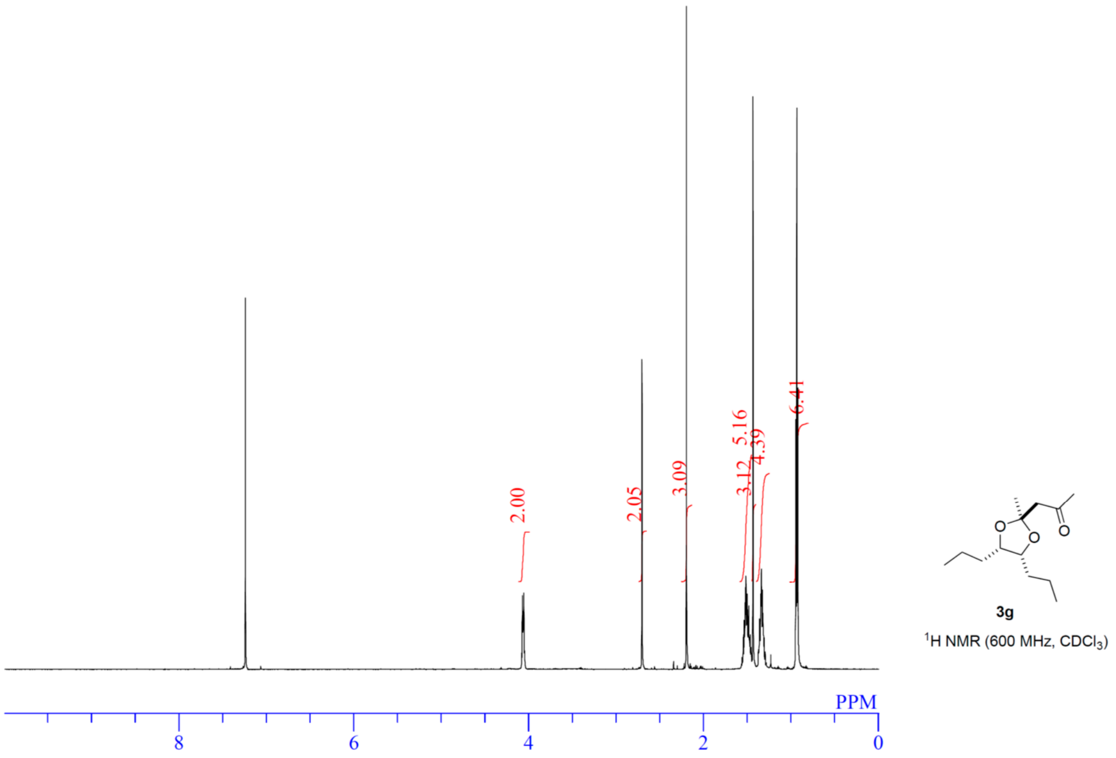

Figure S15. Proton NMR of **3g**.

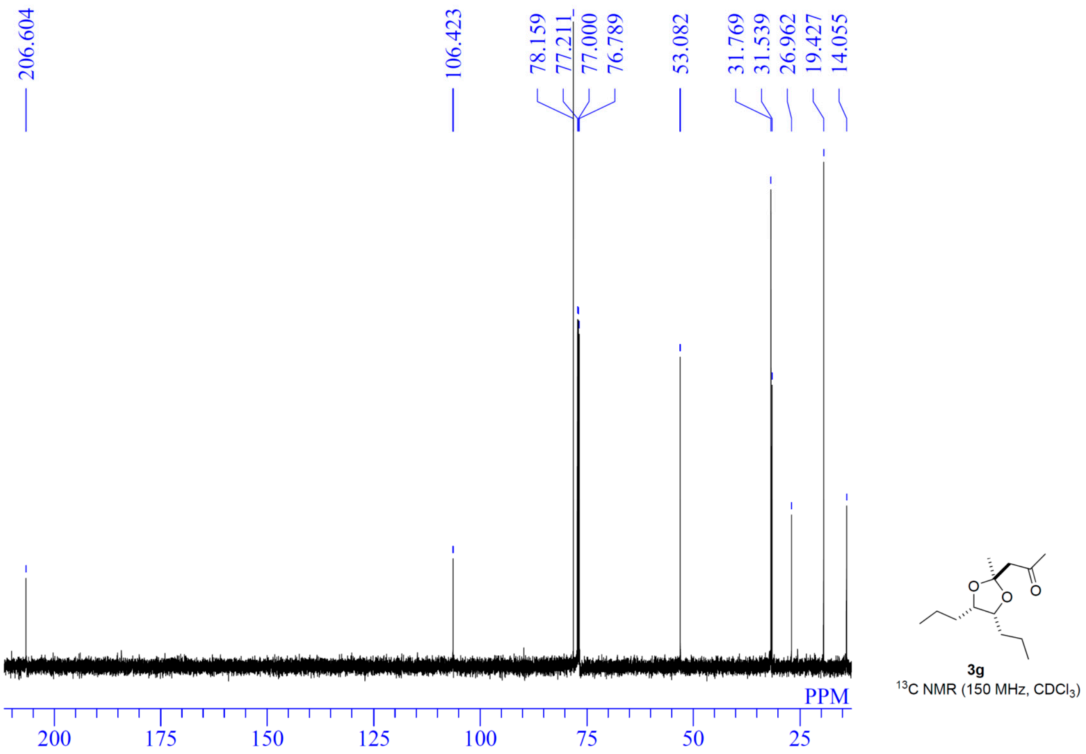

Figure S16. Carbon-13 NMR of **3g**.

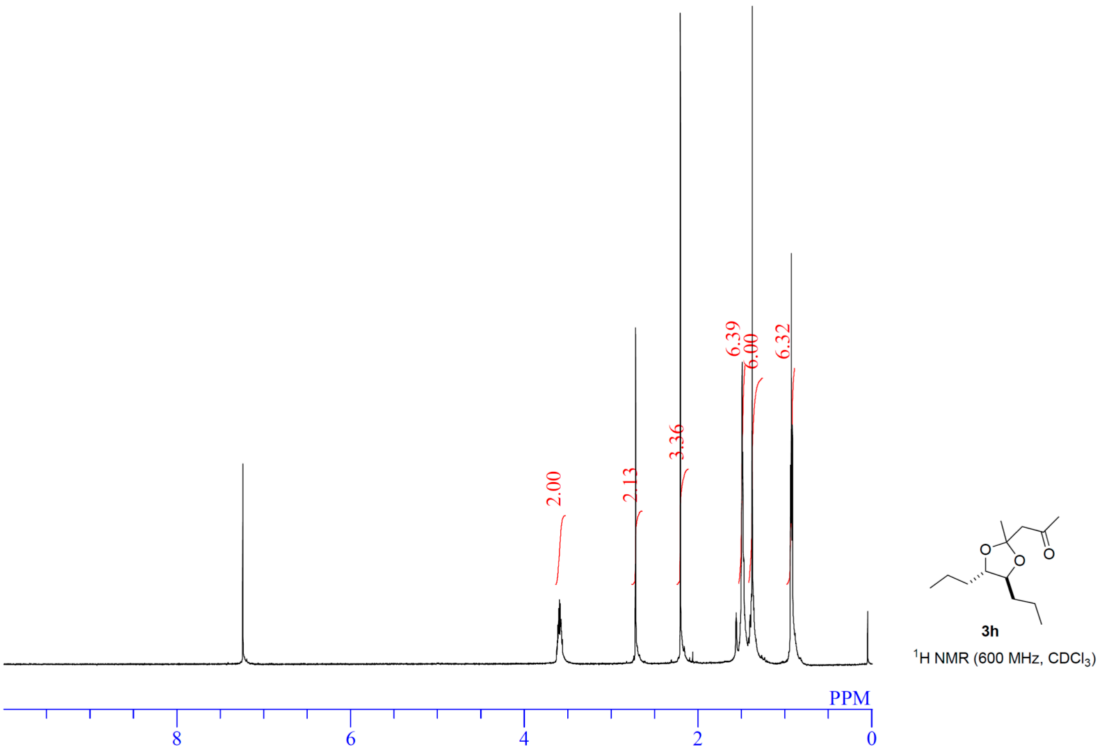

Figure S17. Proton NMR of **3h**.

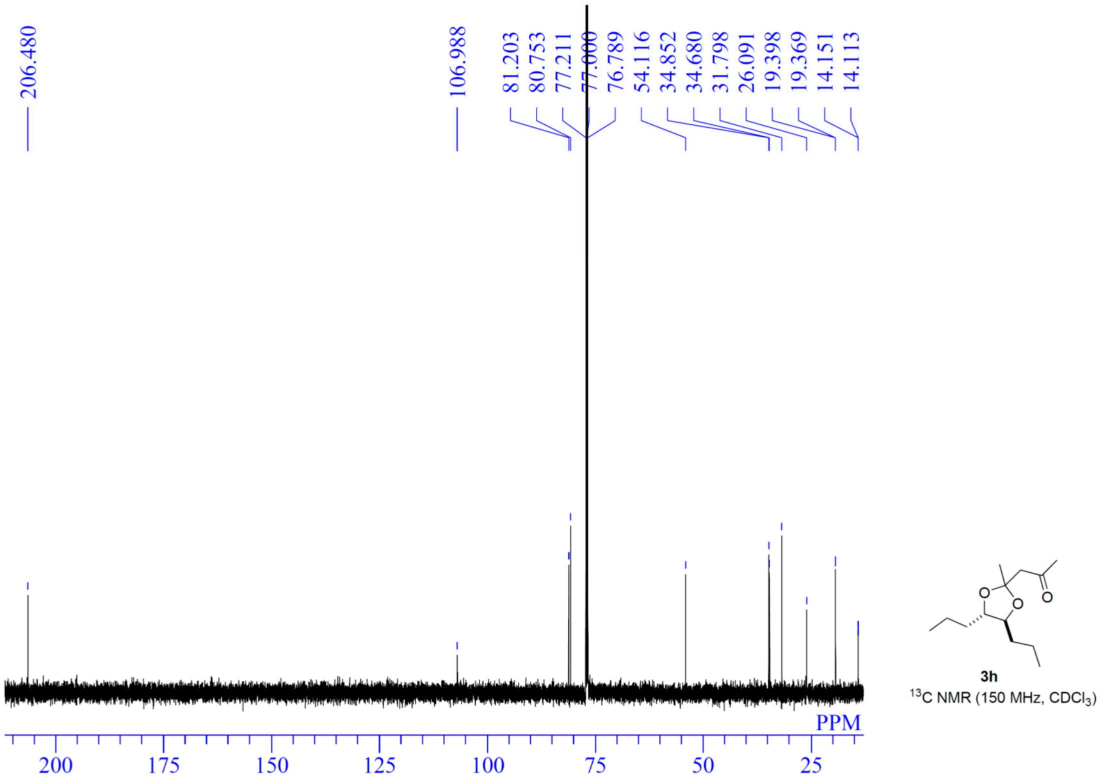

Figure S18. Carbon-13 NMR of **3h**.

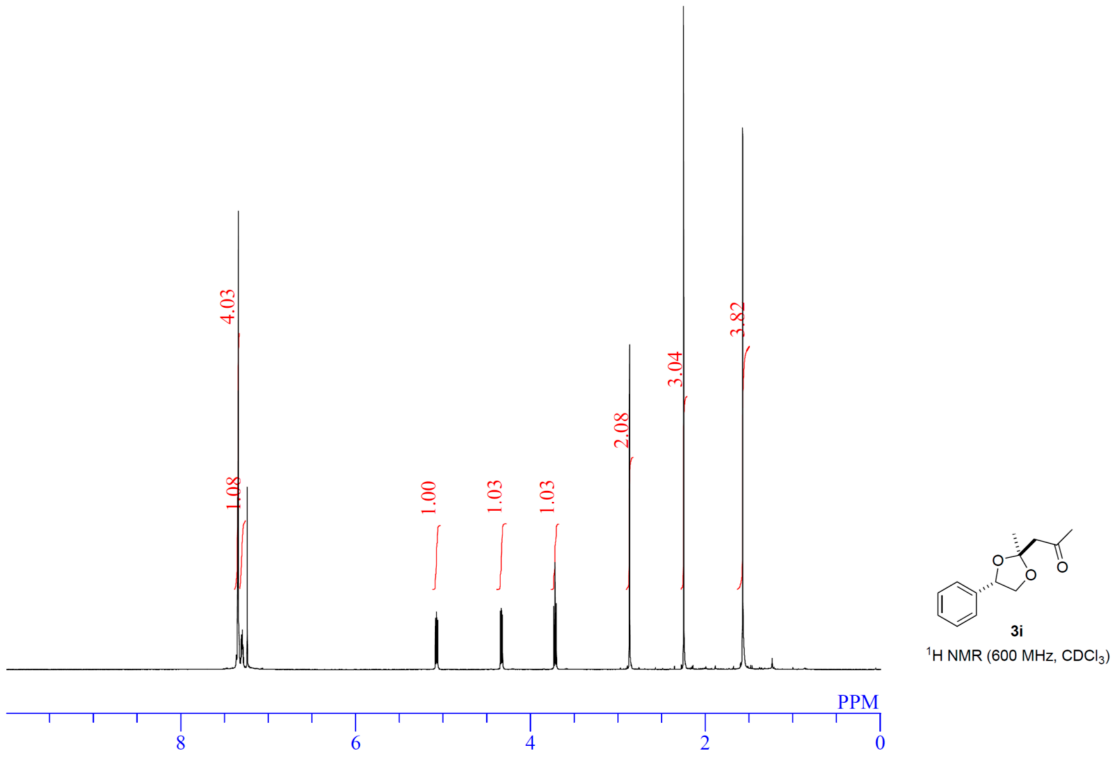

Figure S19. Proton NMR of **3i**.

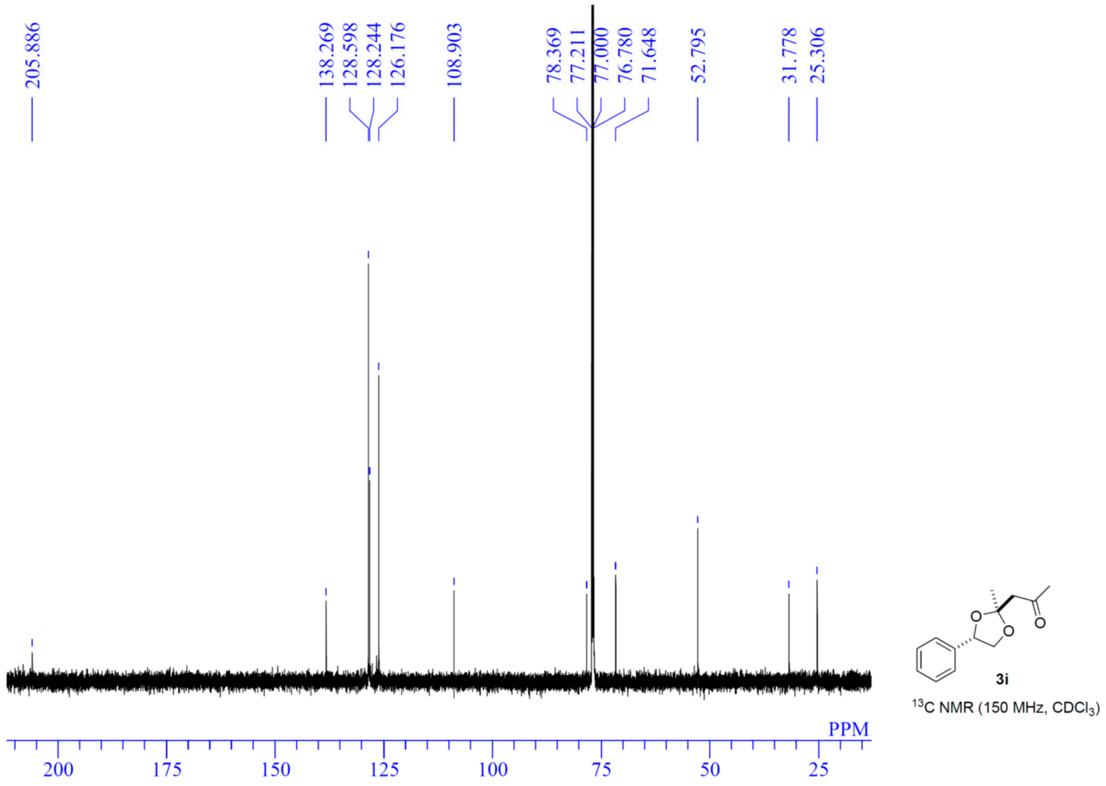

Figure S20. Carbon-13 NMR of **3i**.

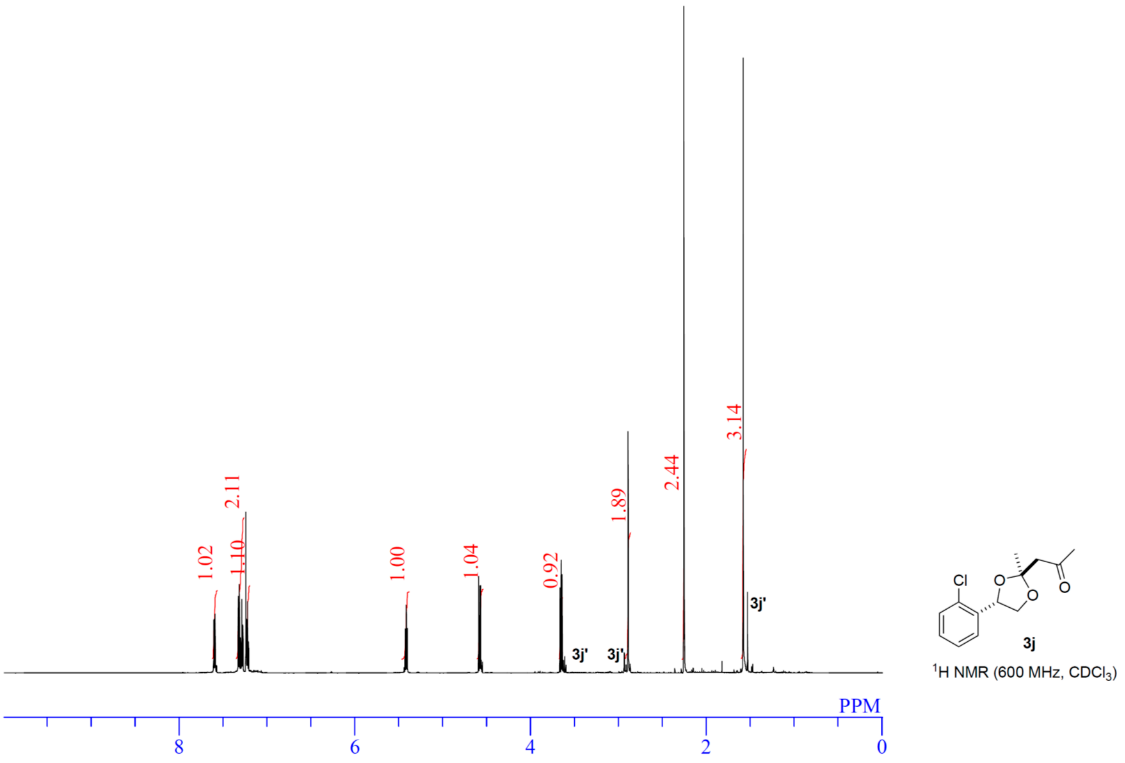

Figure S21. Proton NMR of **3j**.

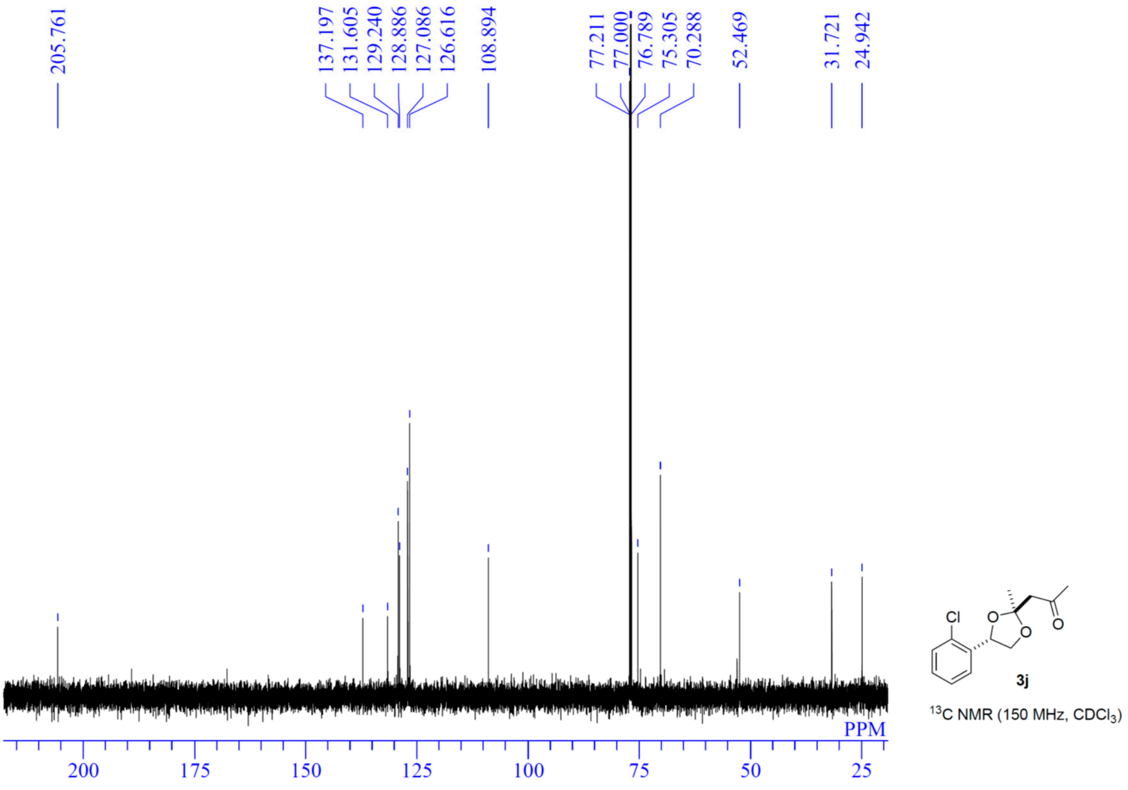

Figure S22. Carbon-13 NMR of **3j**.

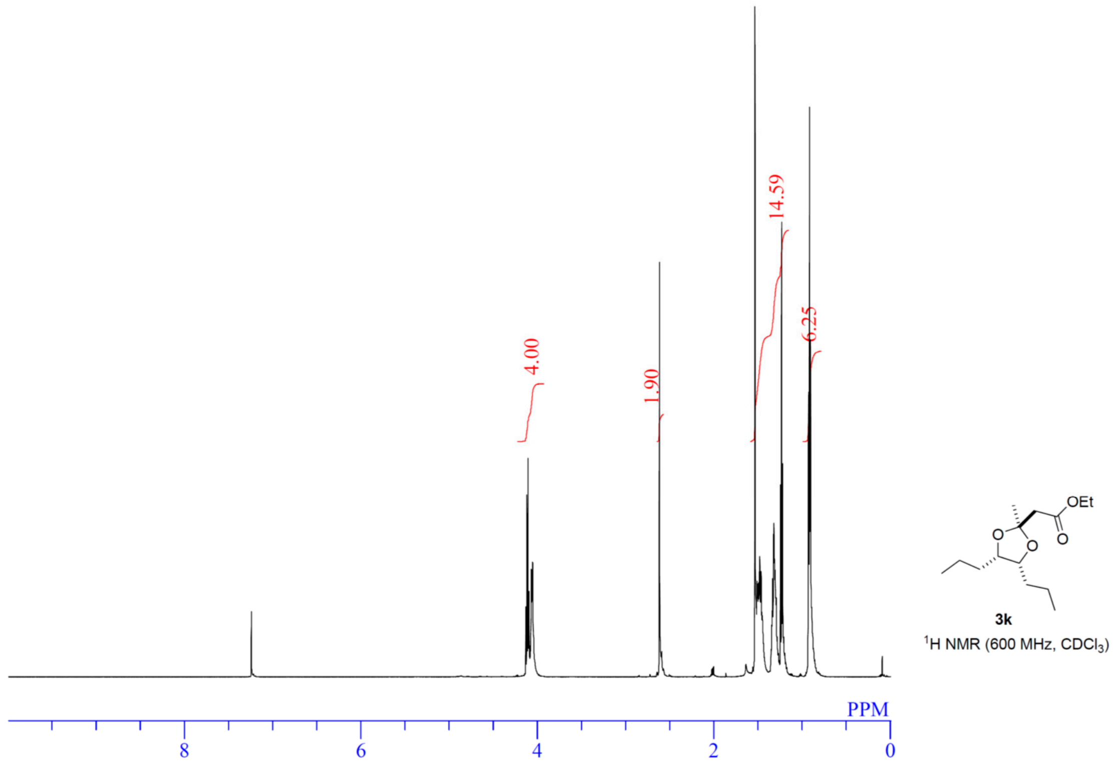

Figure S23. Proton NMR of **3k**.

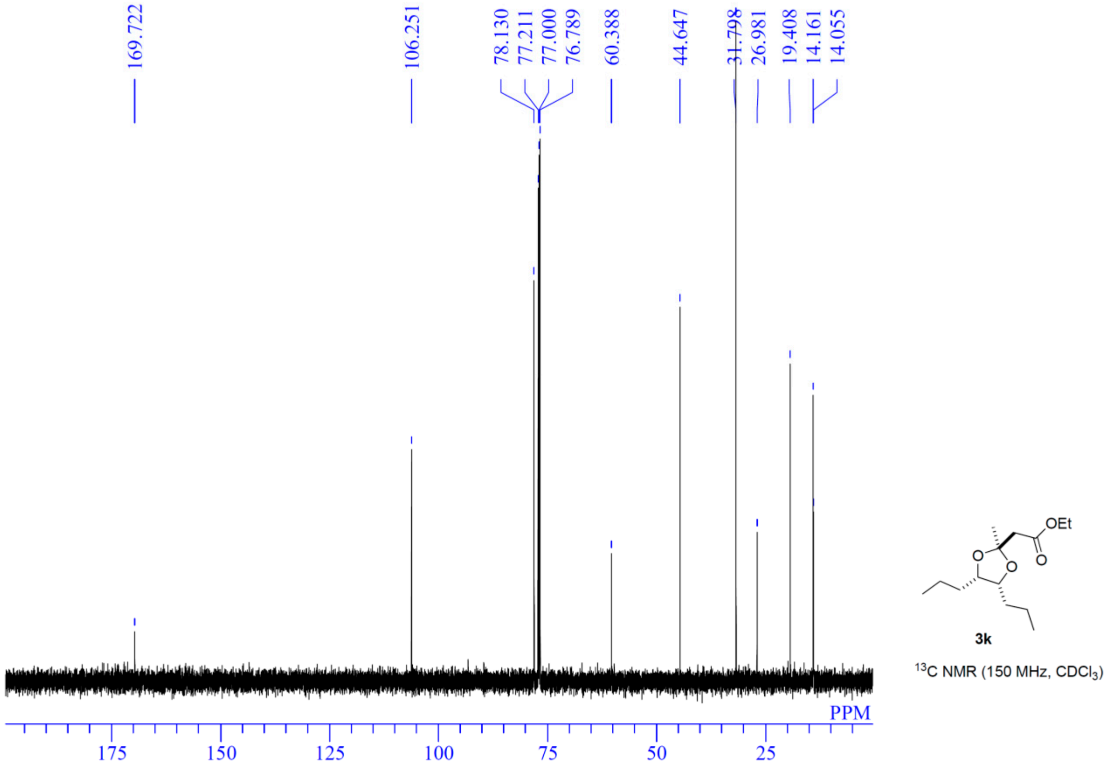

Figure S24. Carbon-13 NMR of **3k**.

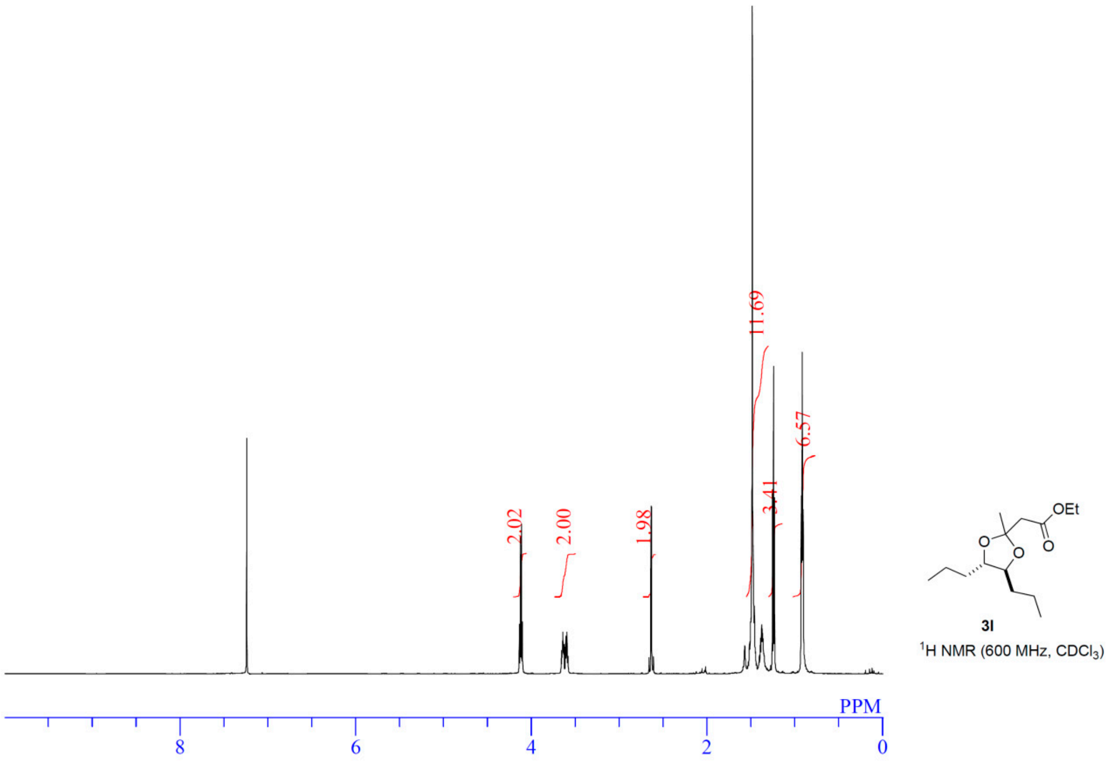

Figure S25. Proton NMR of **3l**.

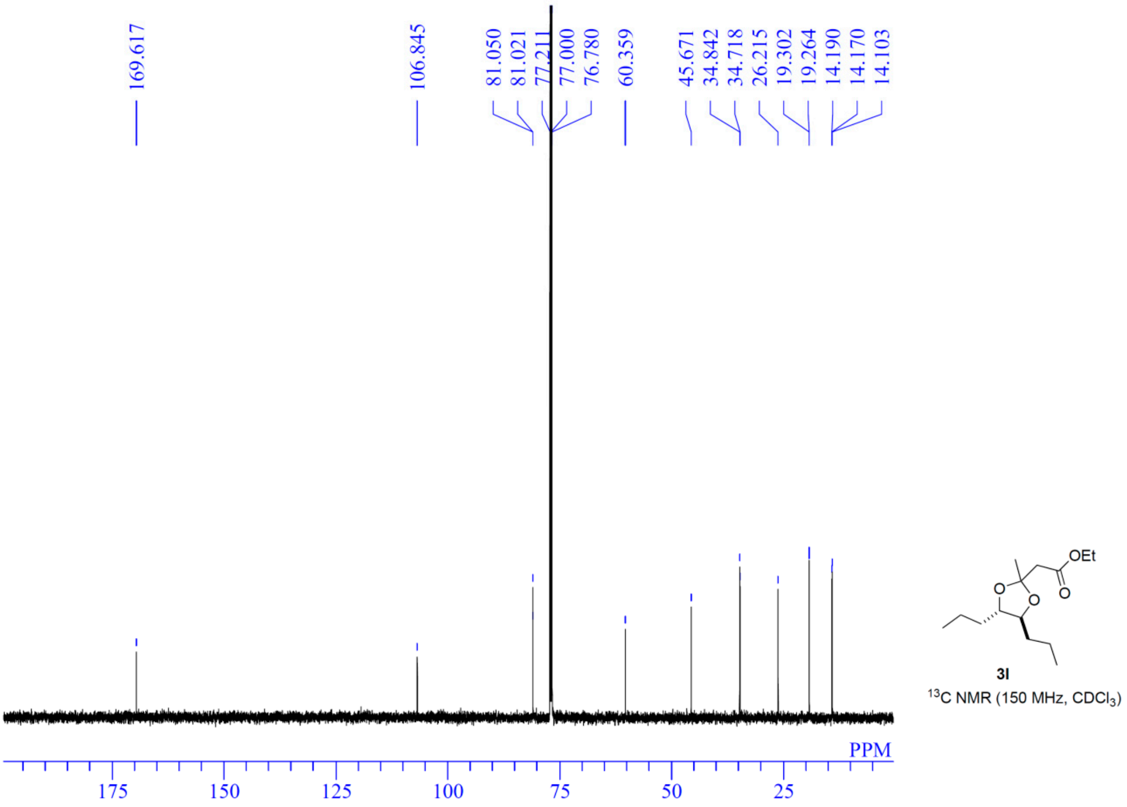

Figure S26. Carbon-13 NMR of **3l**.

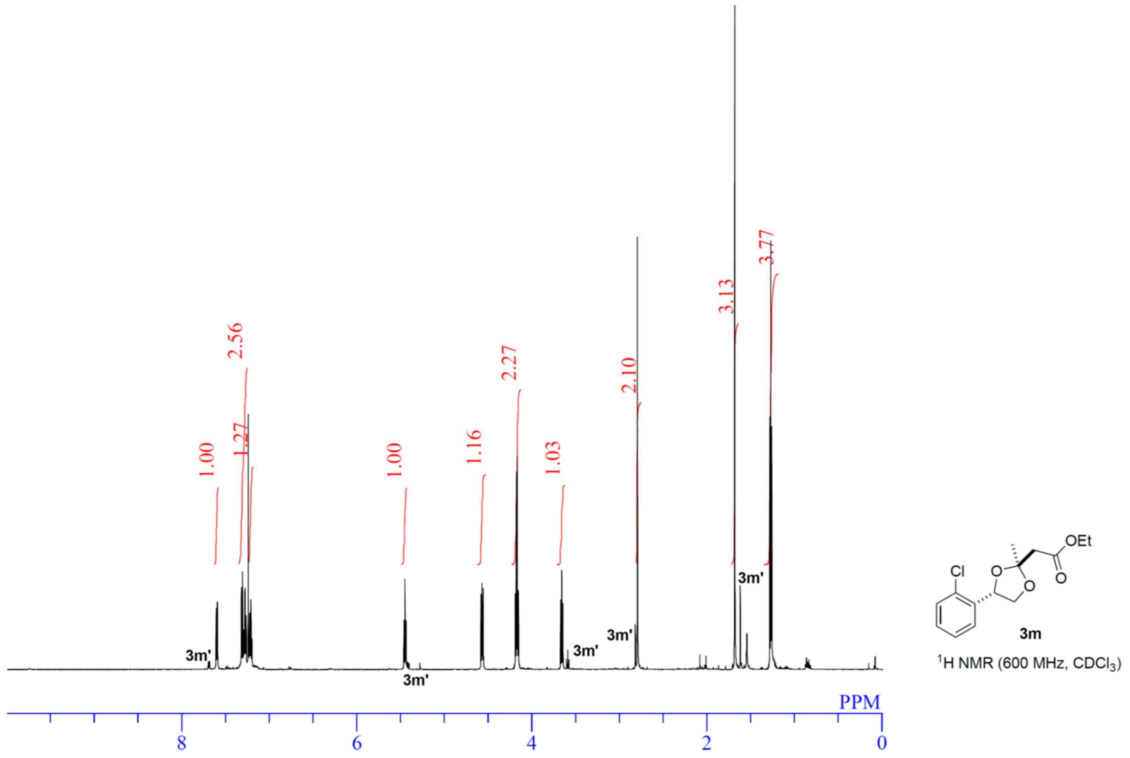

Figure S27. Proton NMR of **3m**.

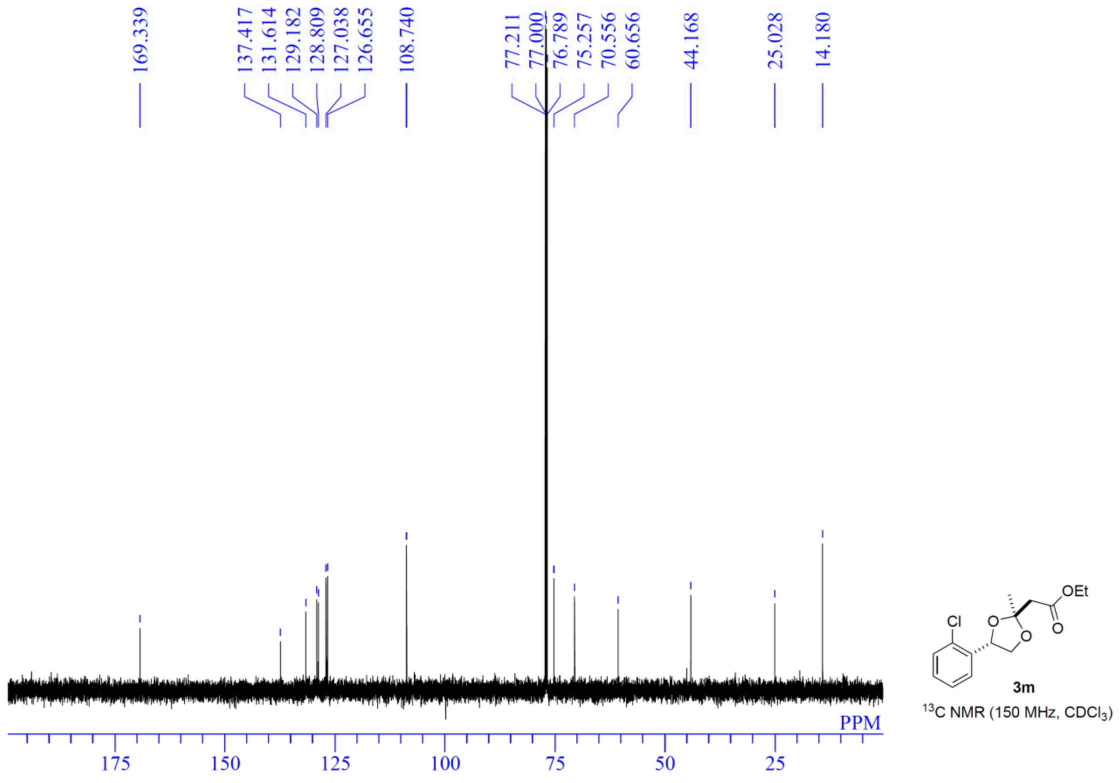

Figure S28. Carbon-13 NMR of **3m**.

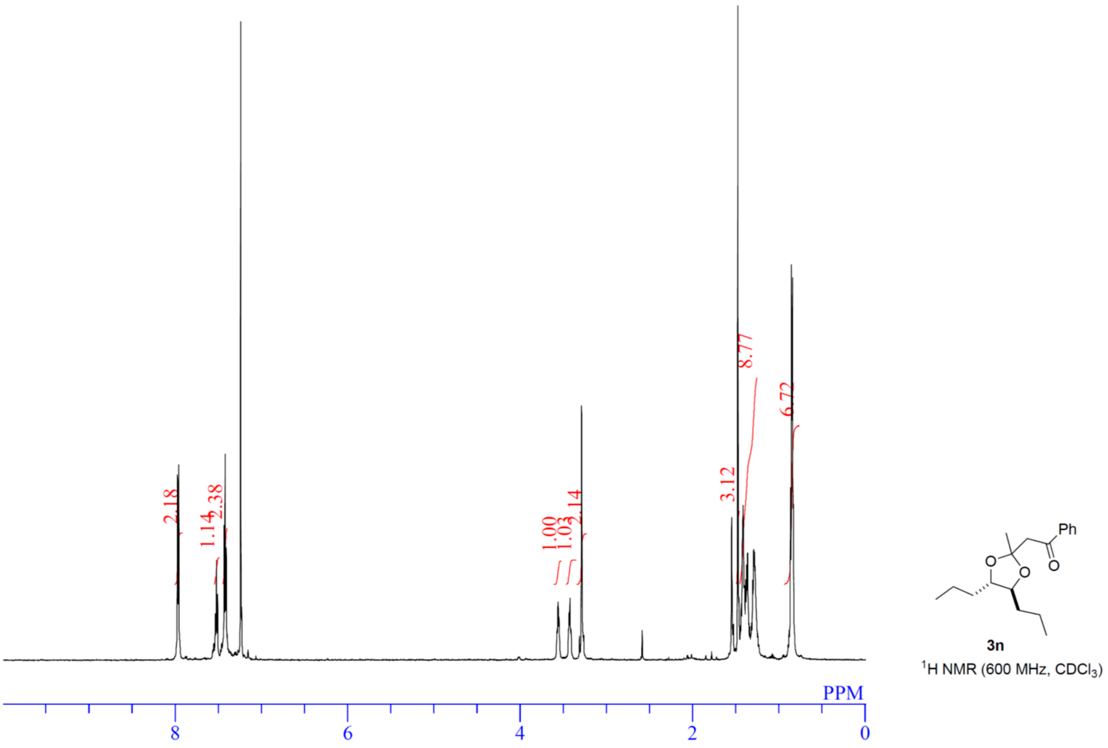

Figure S29. Proton NMR of **3n**.

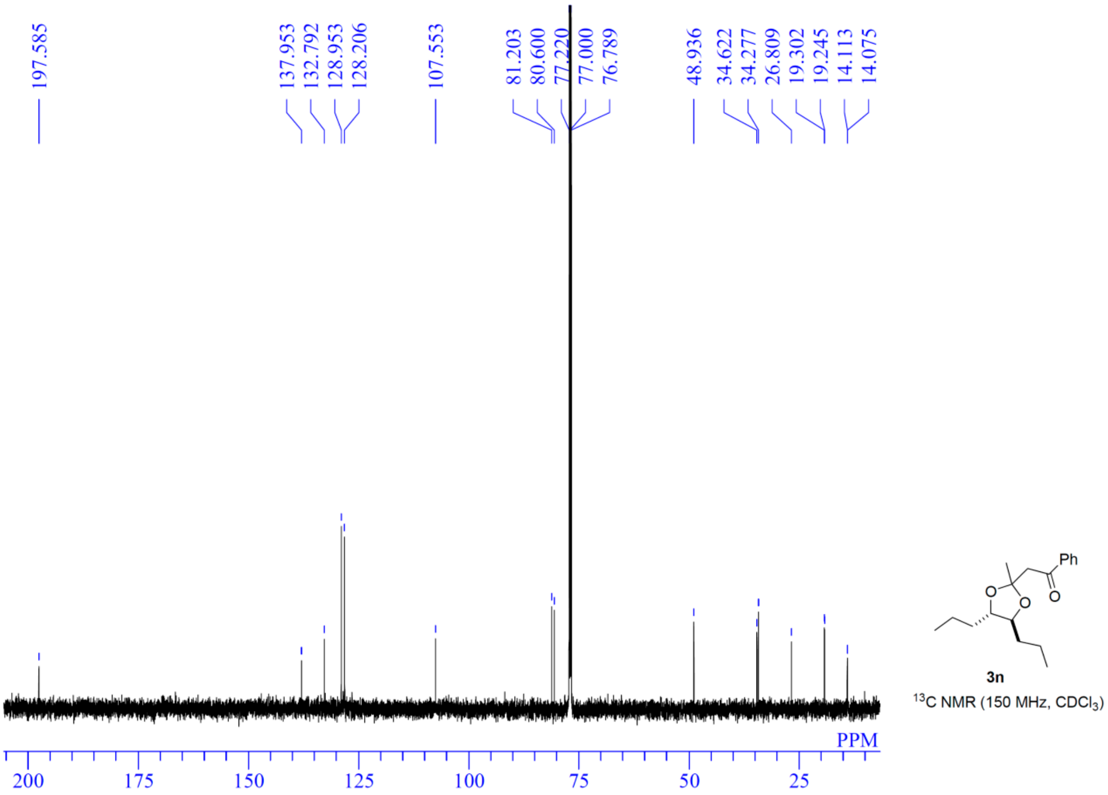

Figure S30. Carbon-13 NMR of **3n**.

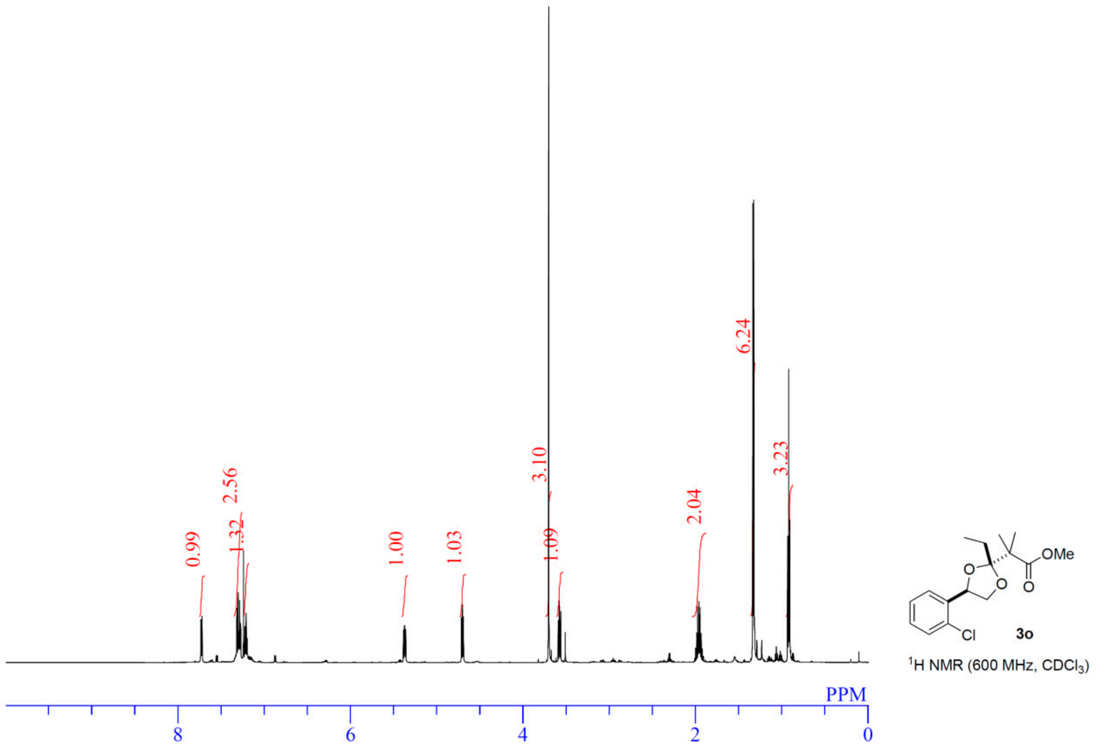

Figure S31. Proton NMR of **3o**.

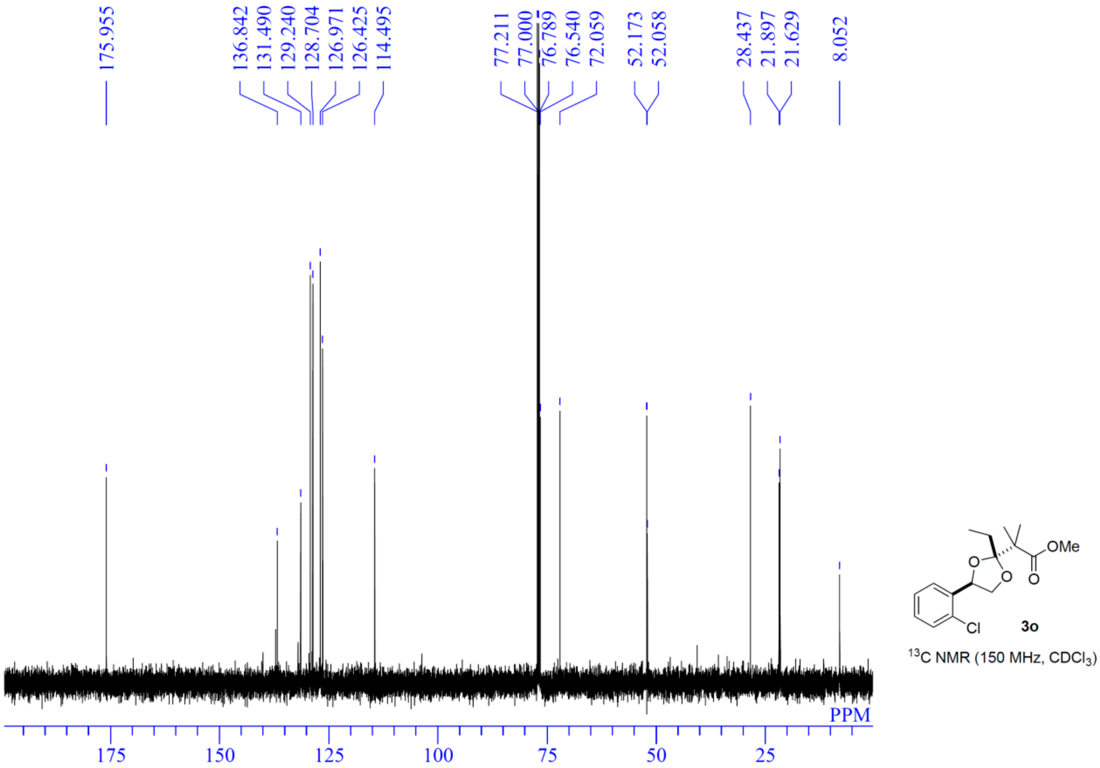

Figure S32. Carbon-13 NMR of **3o**.

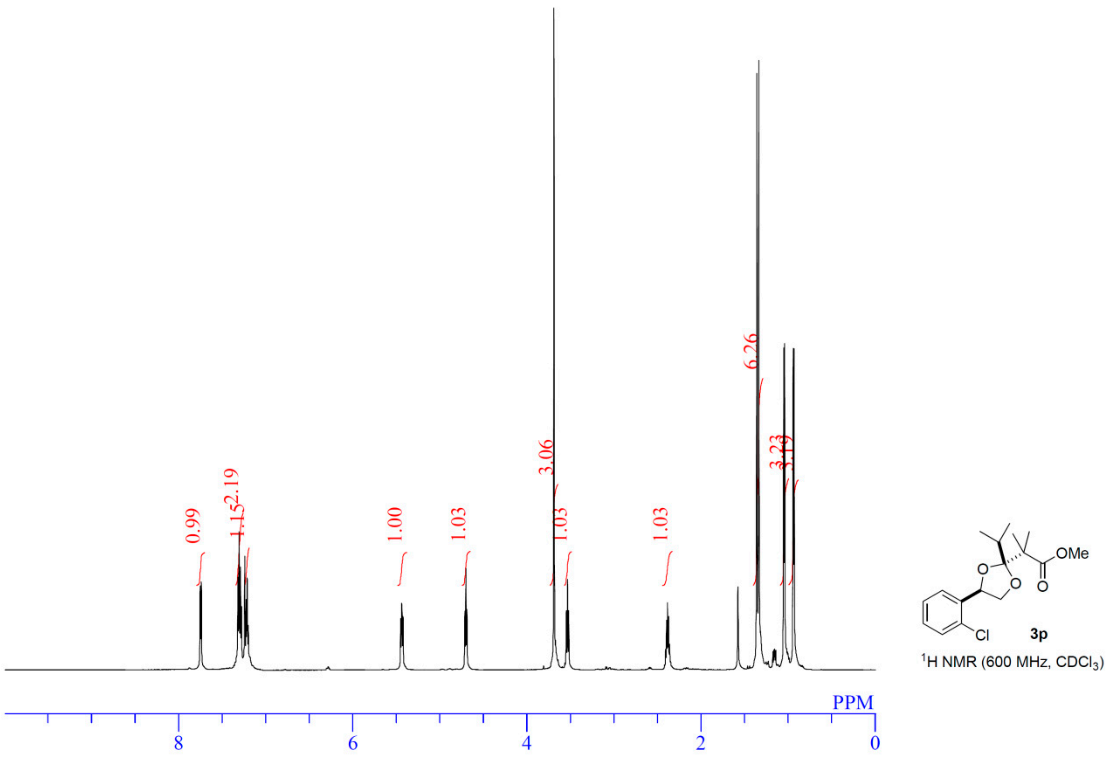

Figure S33. Proton NMR of **3p**.

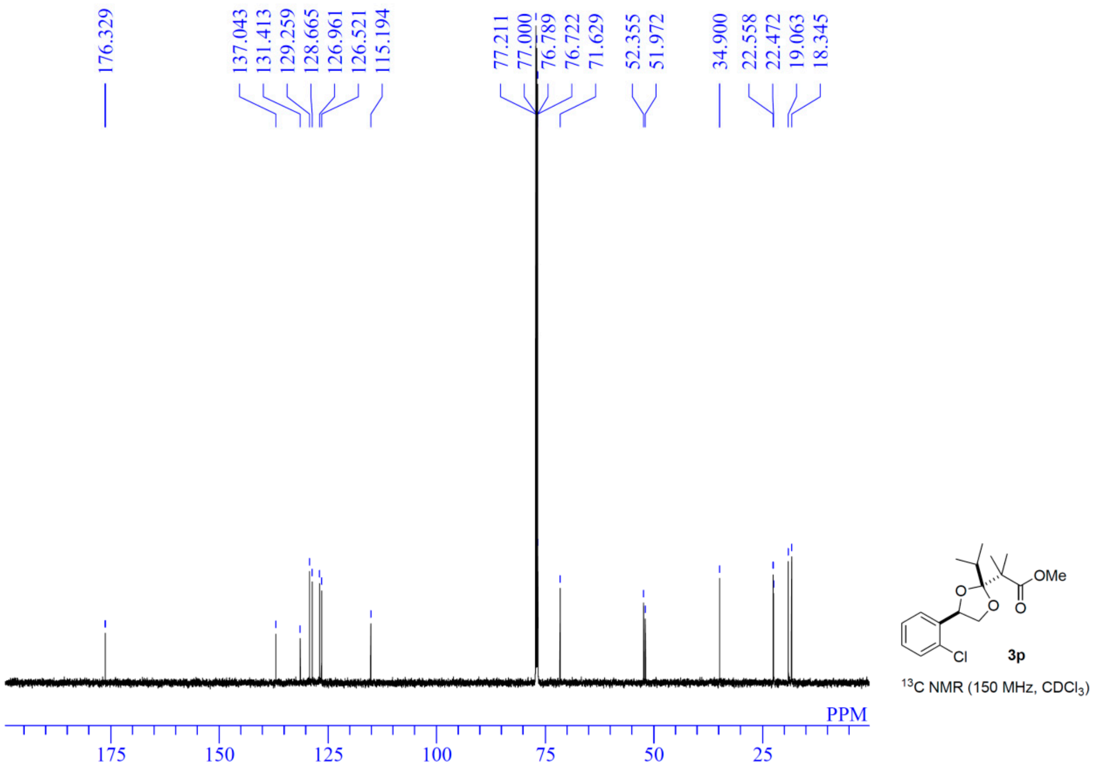

Figure S34. Carbon-13 NMR of **3p**.

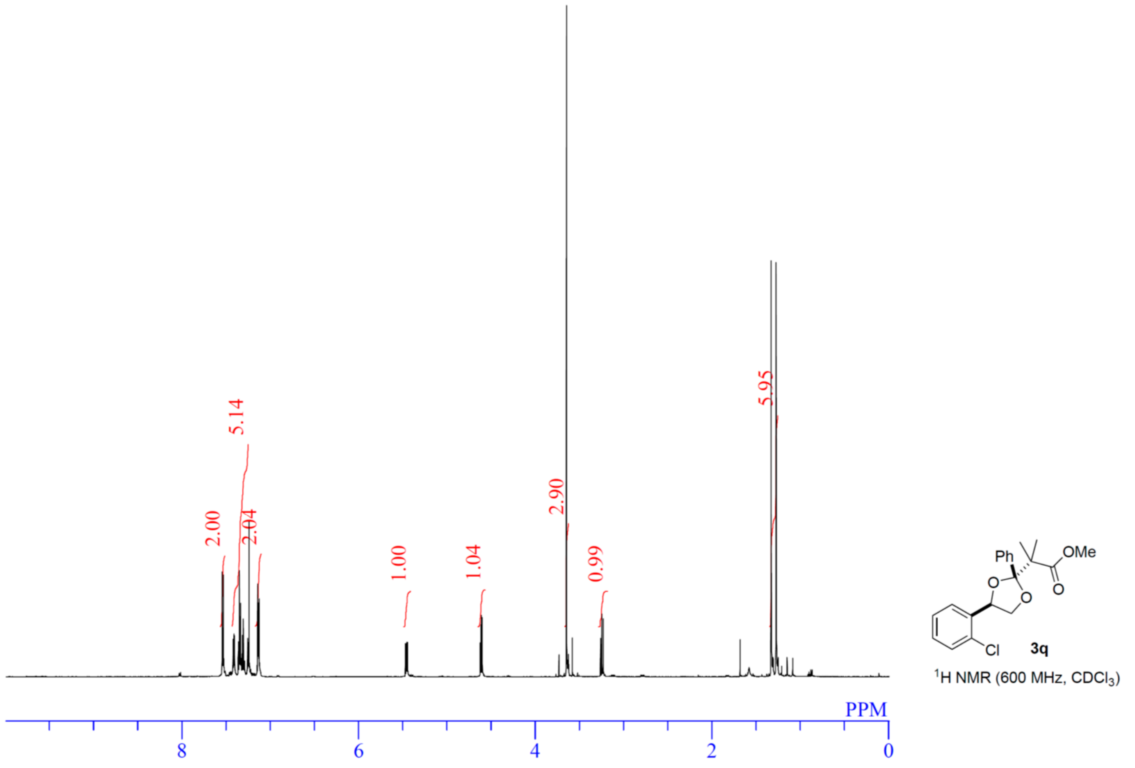

Figure S35. Proton NMR of **3q**.

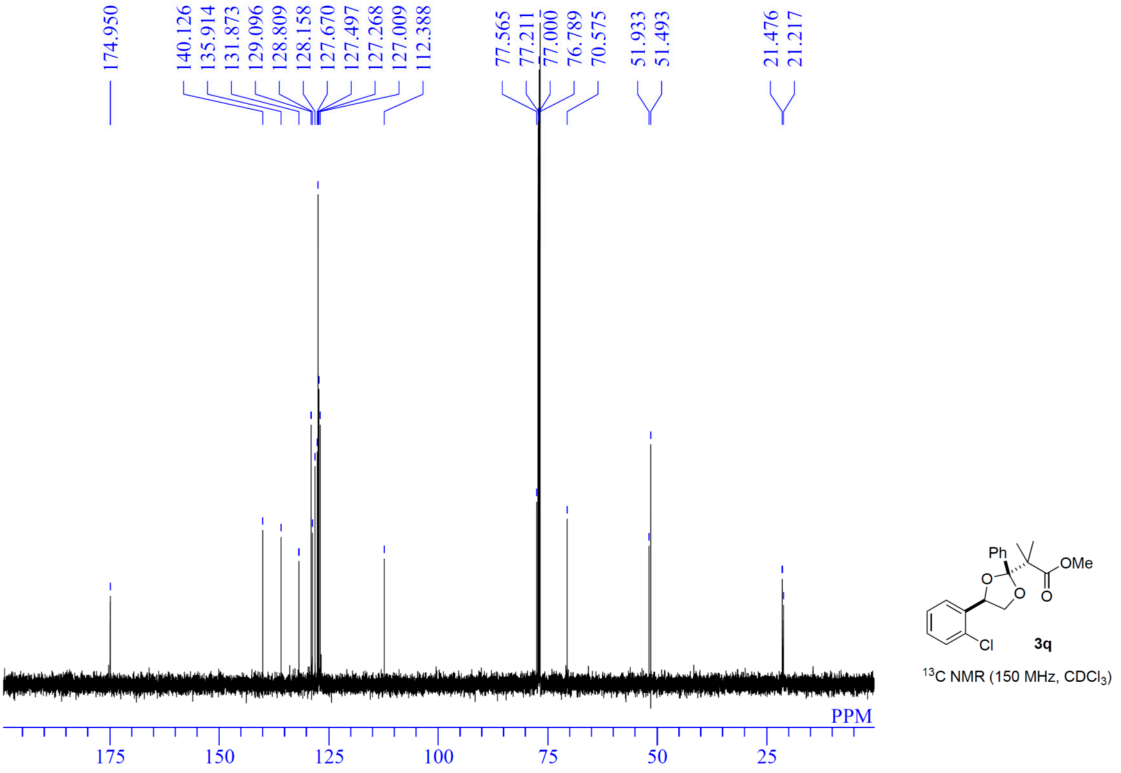

Figure S36. Carbon-13 NMR of **3q**.

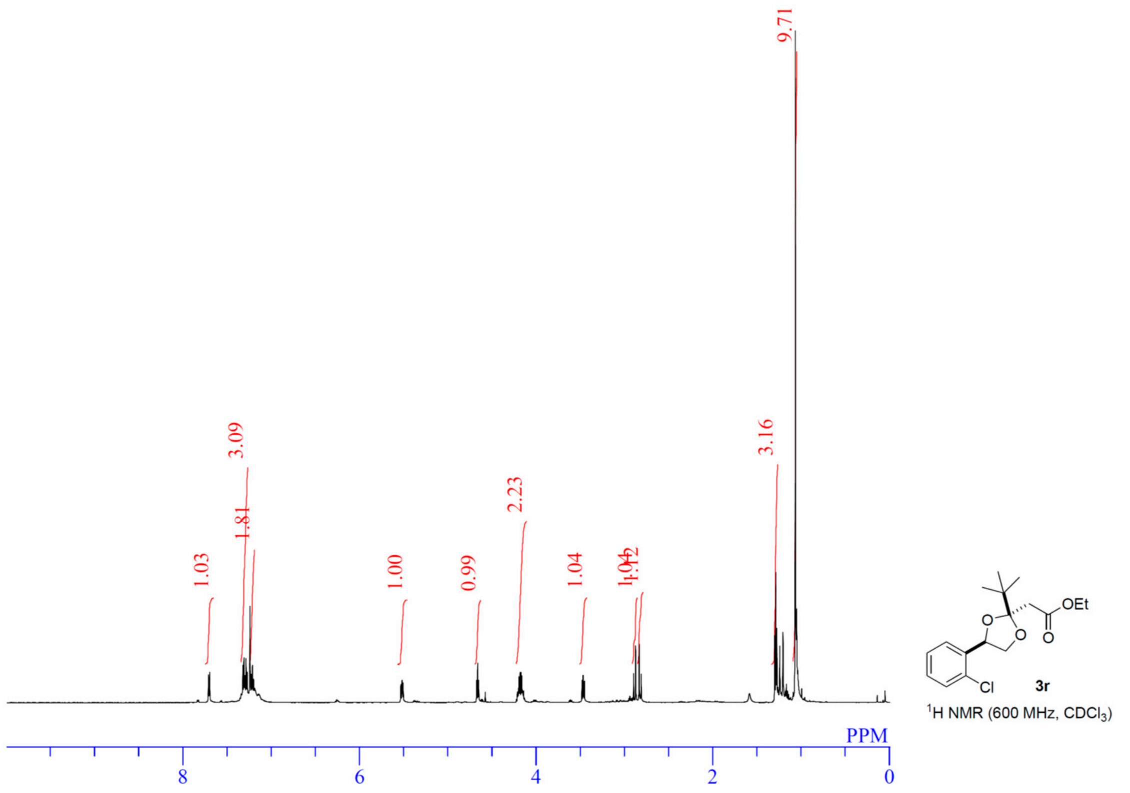

Figure S37. Proton NMR of **3r**.

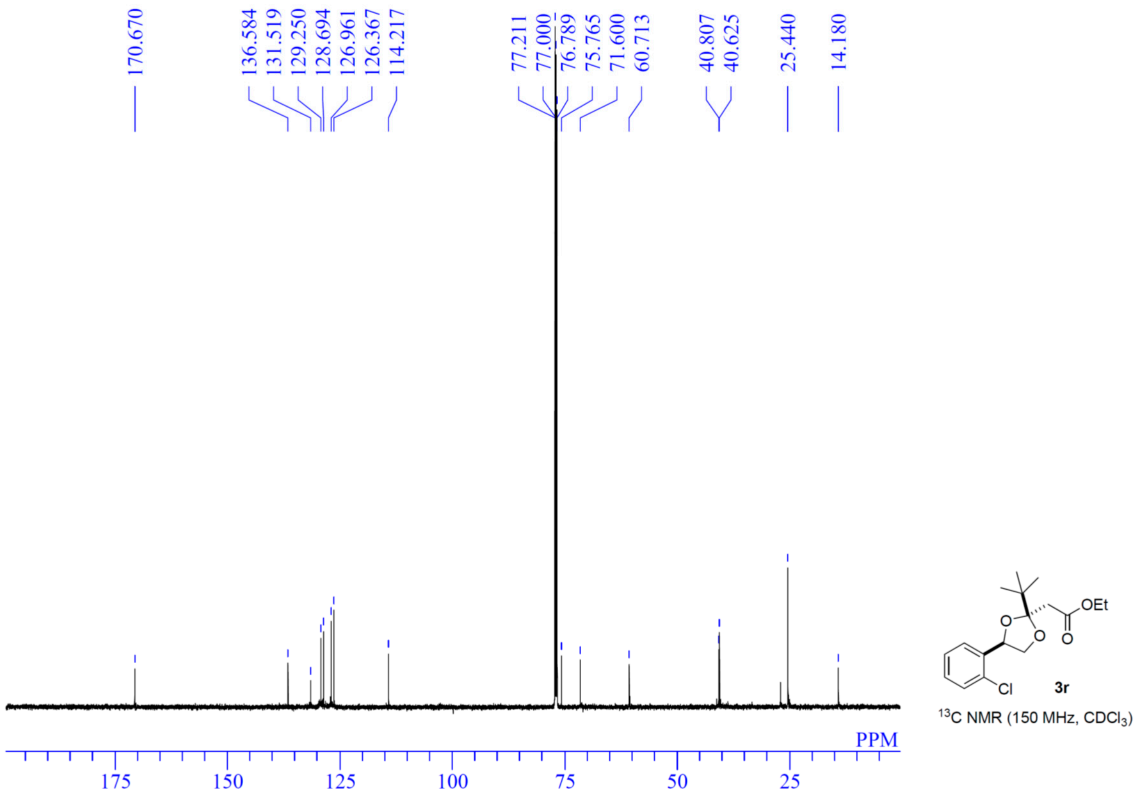

Figure S38. Carbon-13 NMR of **3r**.

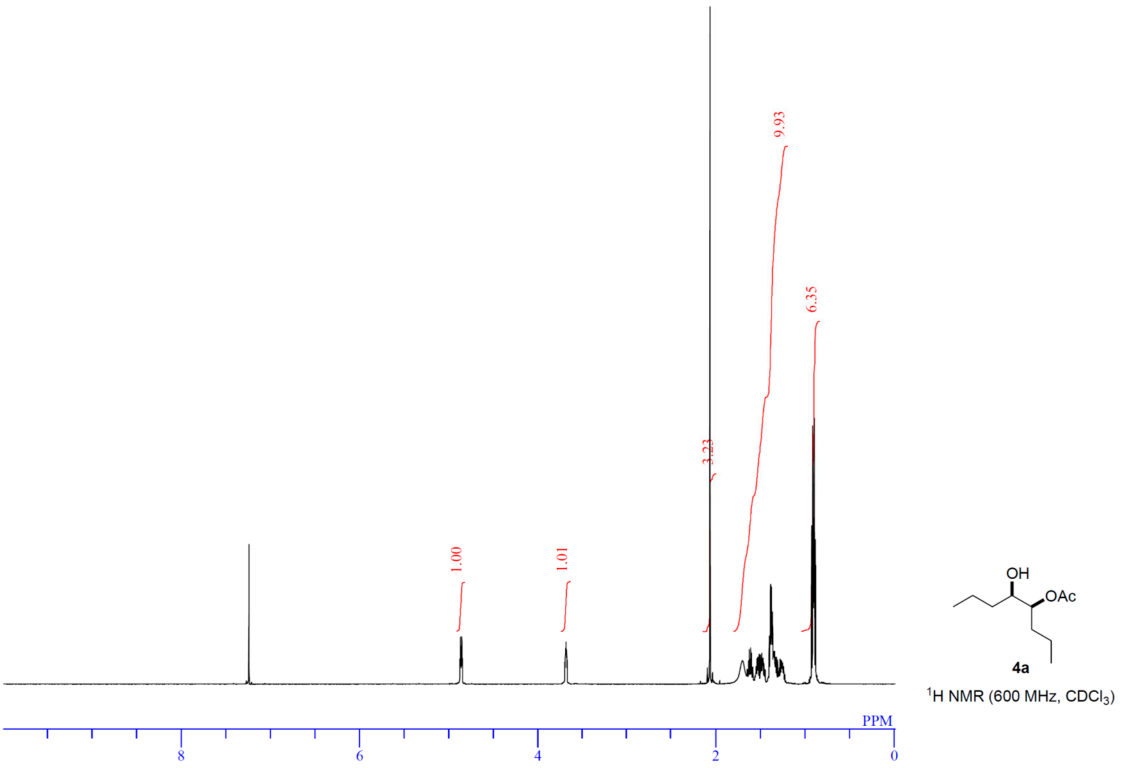

Figure S39. Proton NMR of **4a**.

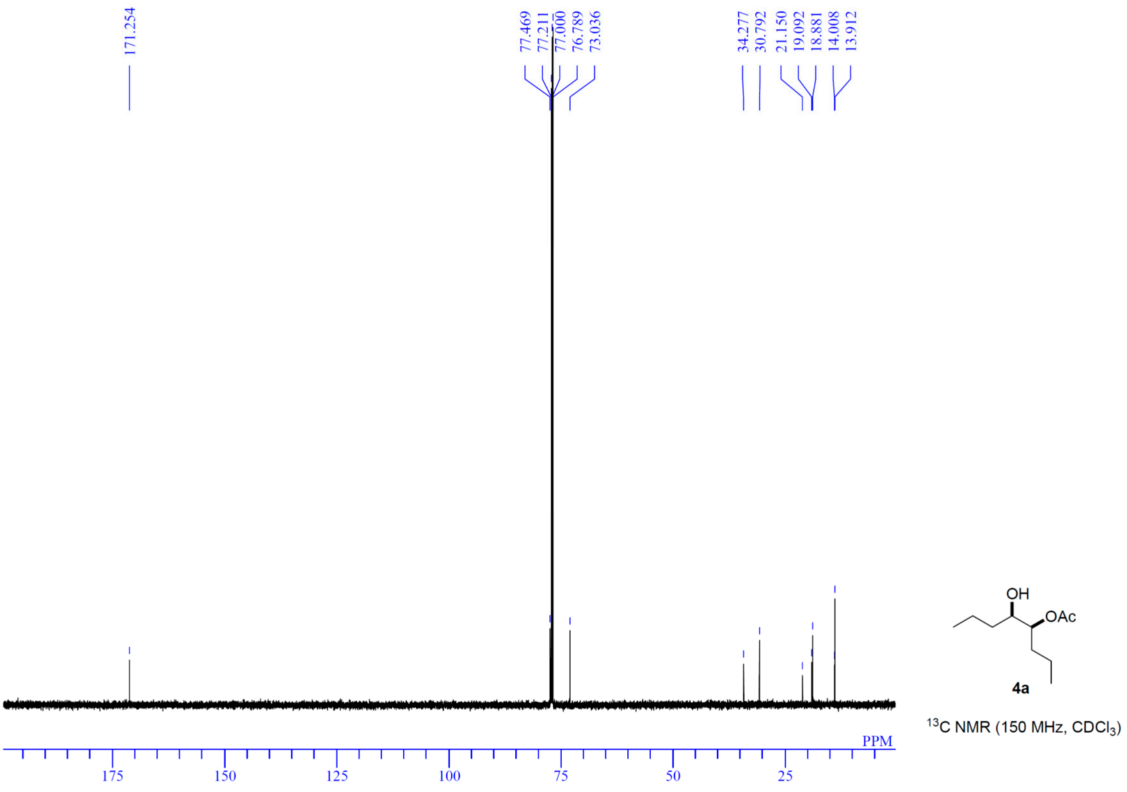

Figure S40. Carbon-13 NMR of **4a**.

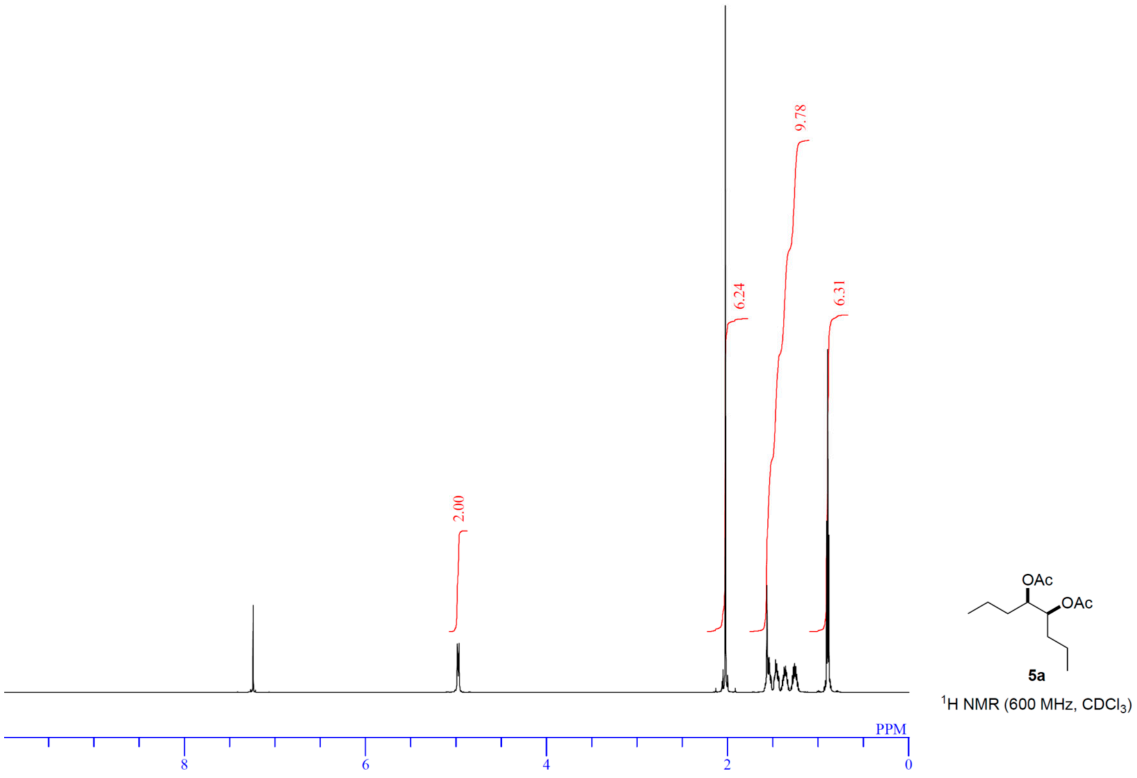

Figure S41. Proton NMR of **5a**.

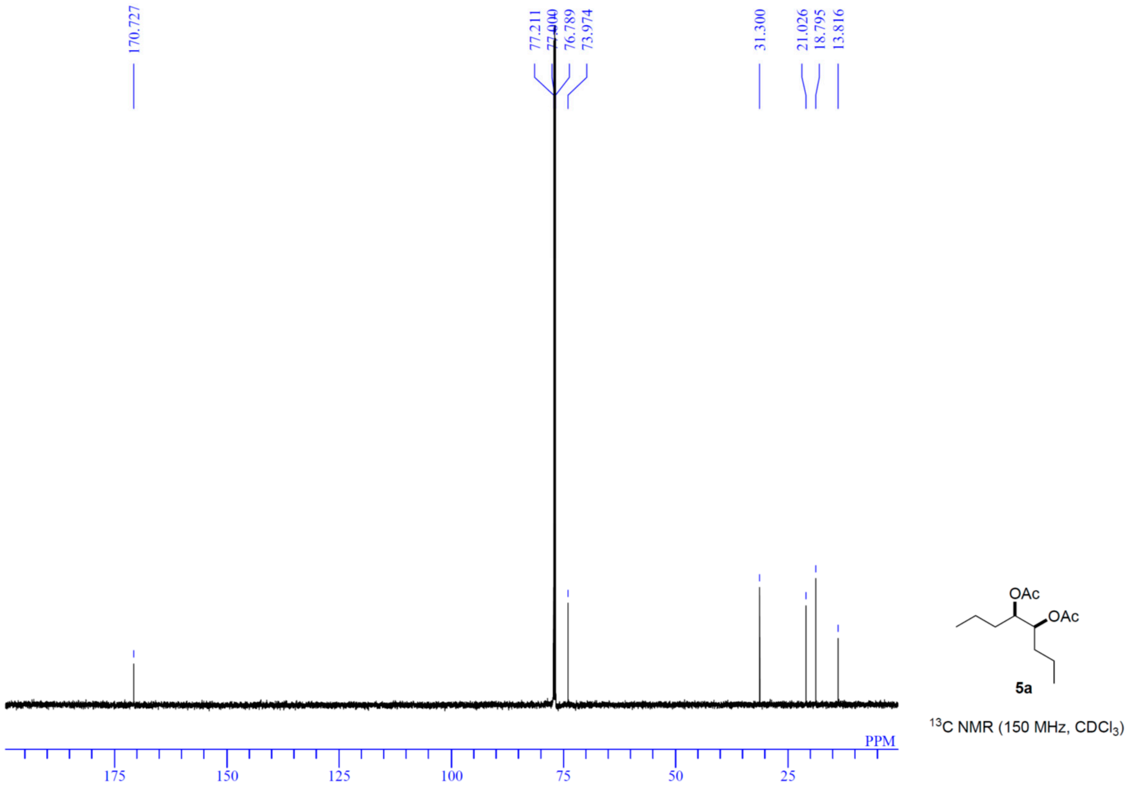

Figure S42. Carbon-13 NMR of **5a**.

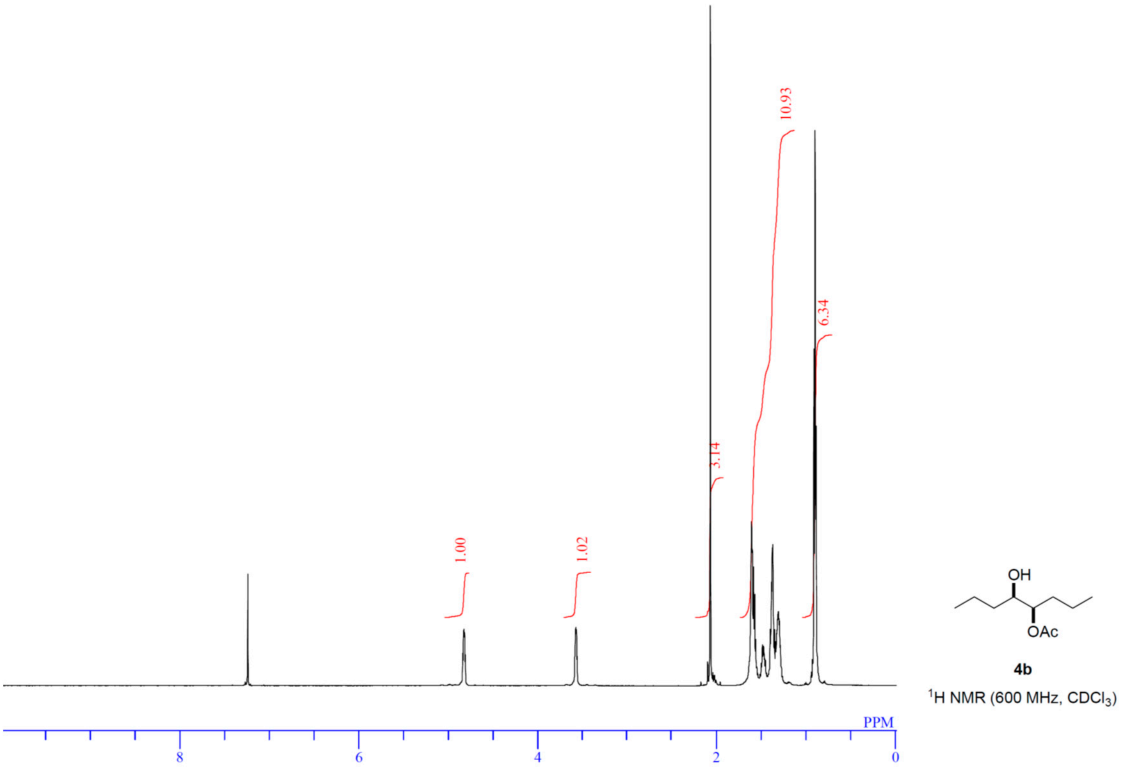

Figure S43. Proton NMR of **4b**.

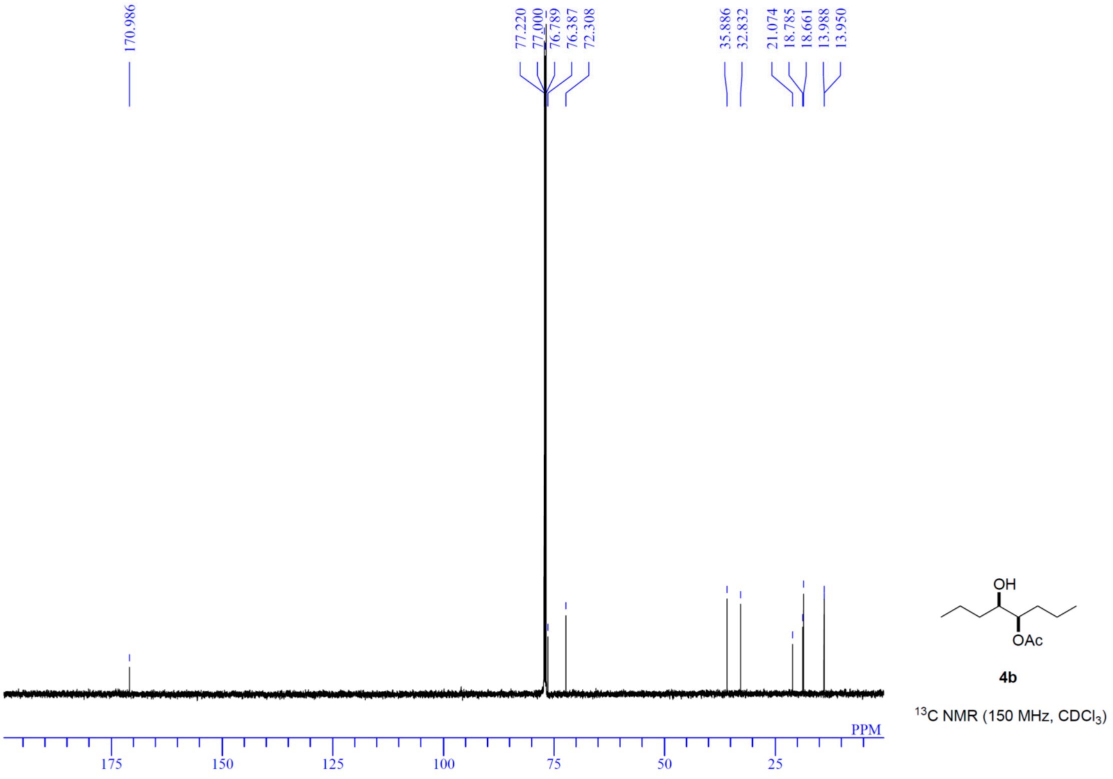

Figure S44. Carbon-13 NMR of **4b**.

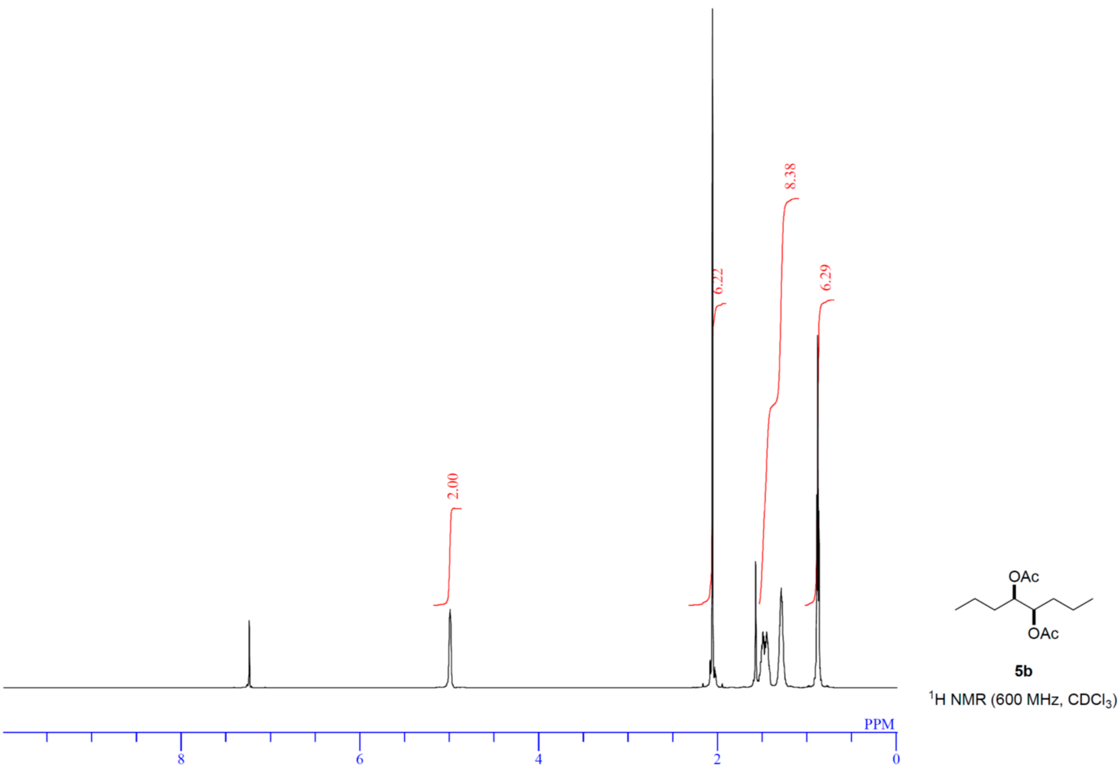

Figure S45. Proton NMR of **5b**.

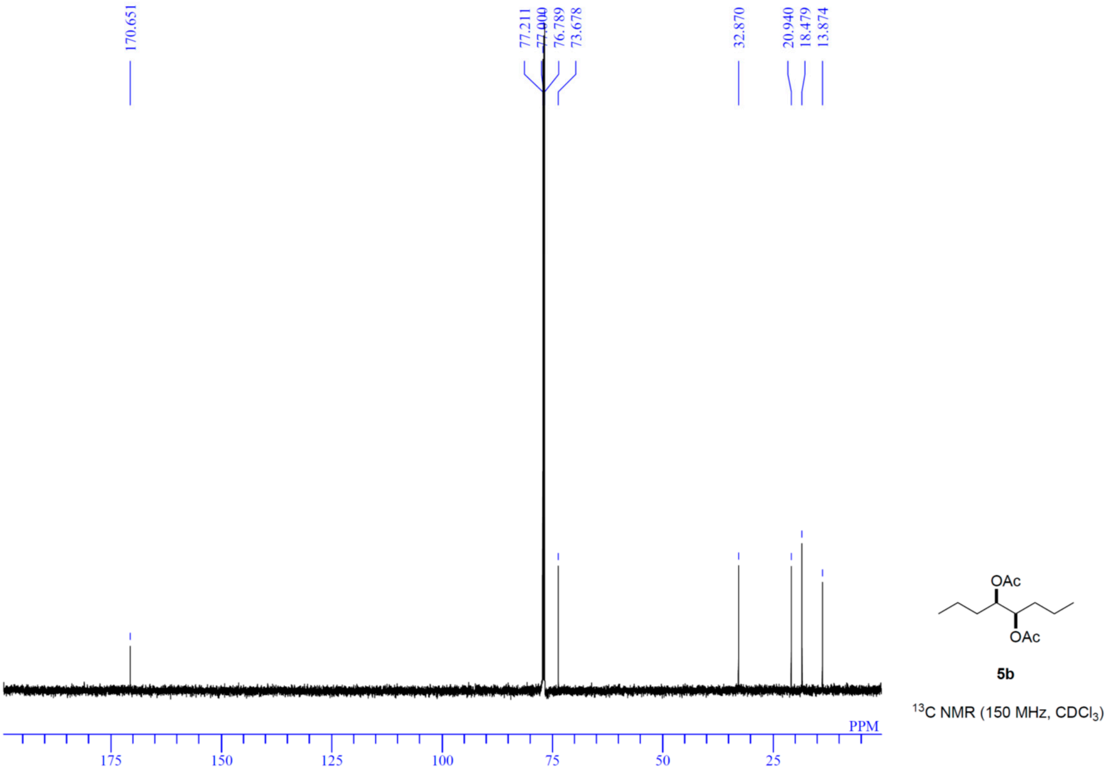

Figure S46. Carbon-13 NMR of **5b**.

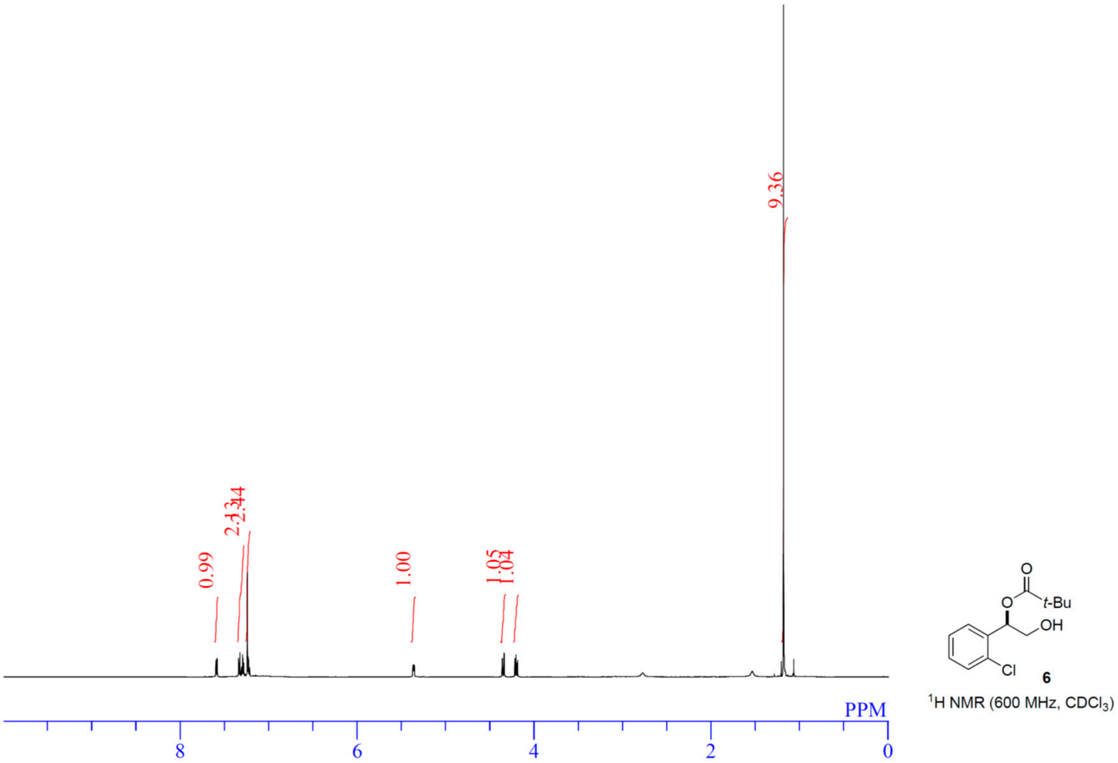

Figure S47. Proton NMR of **6**.

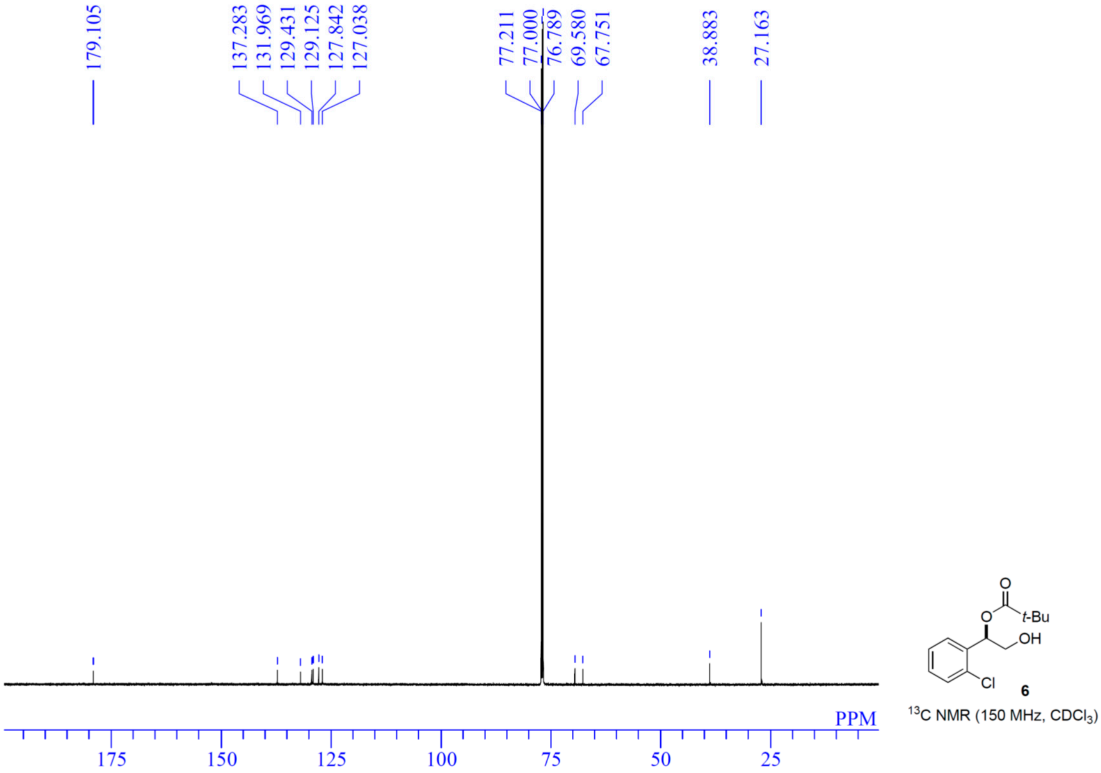

Figure S48. Carbon-13 NMR of **6**.

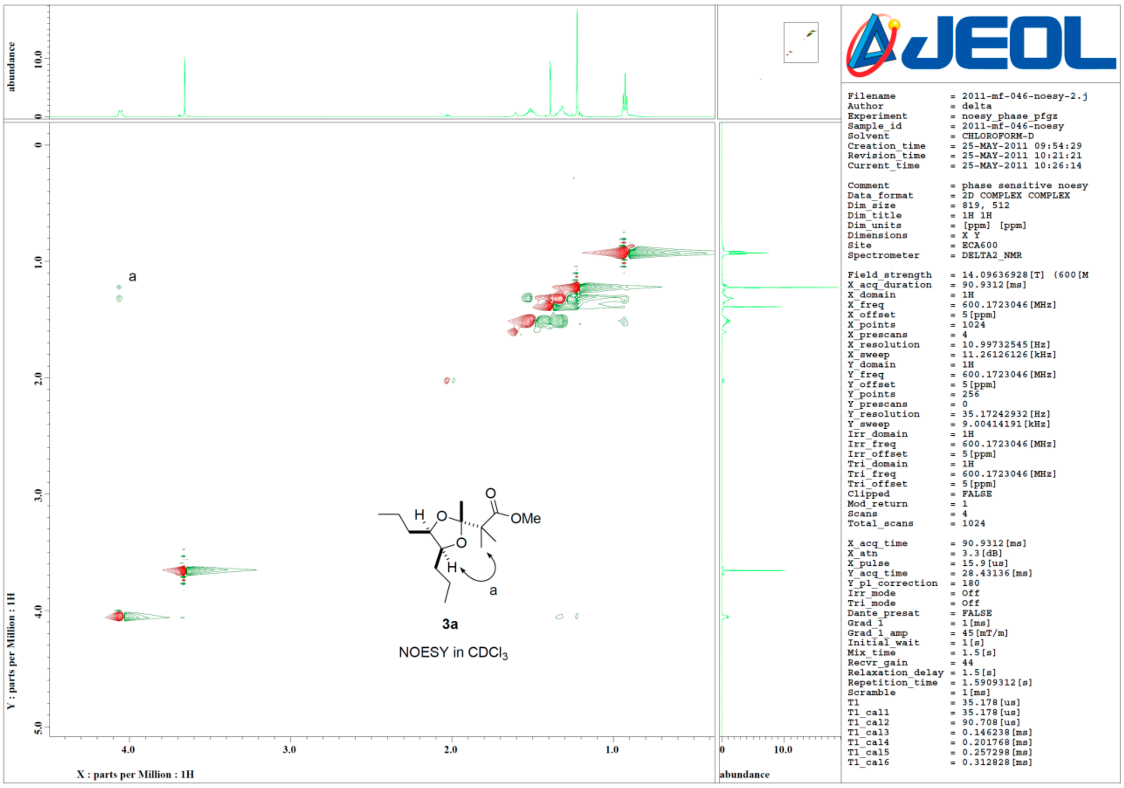

Figure S49. NOESY spectrum of **3a**.

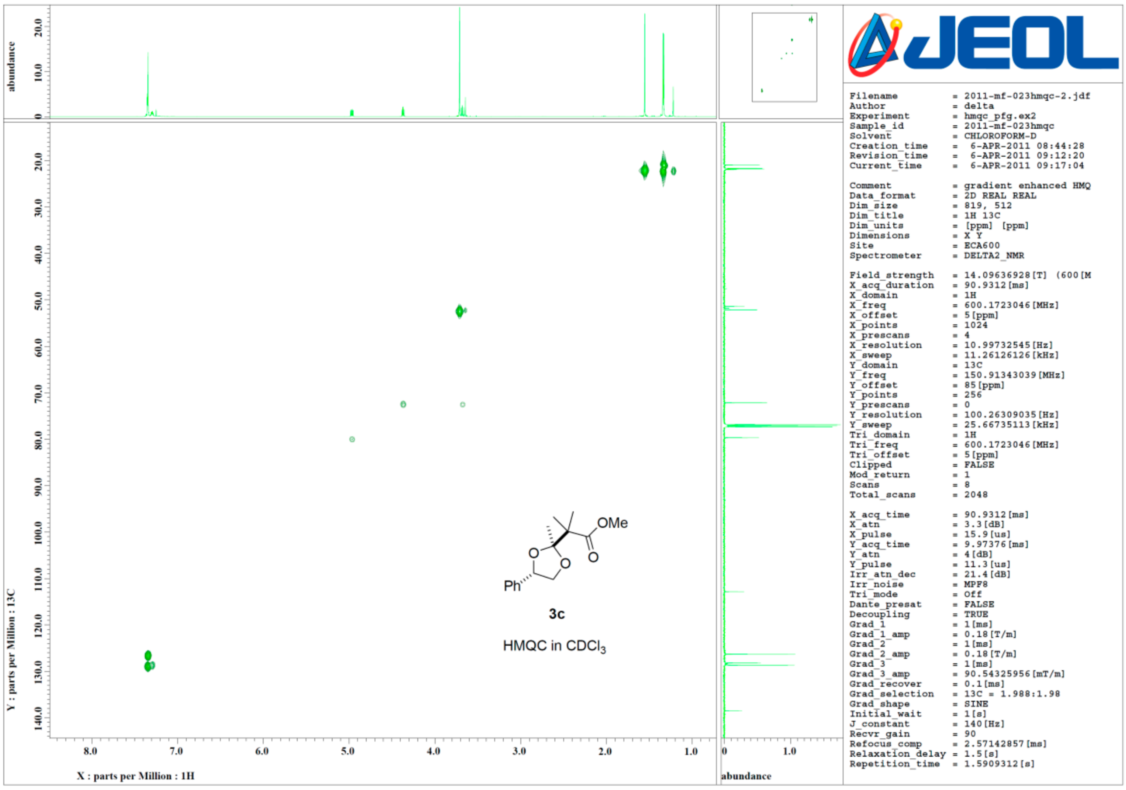

Figure S50. HMQC spectrum of **3c**.

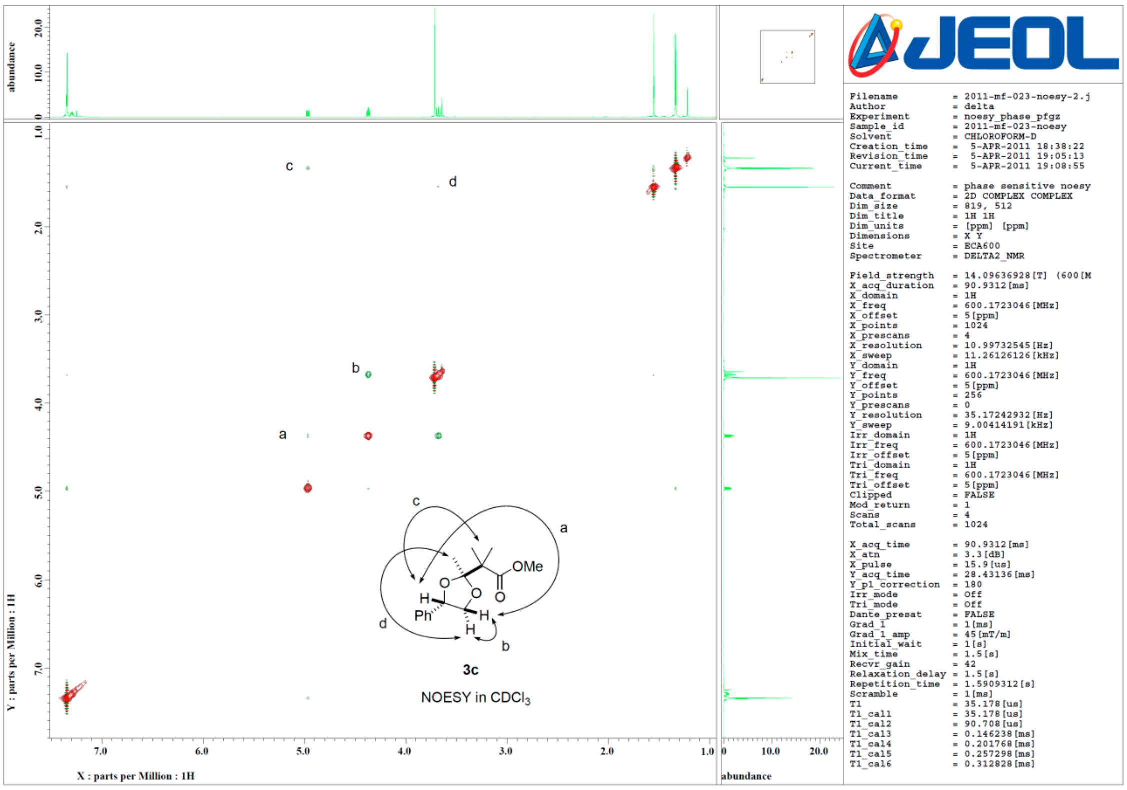

Figure S51. NOESY spectrum of **3c**.

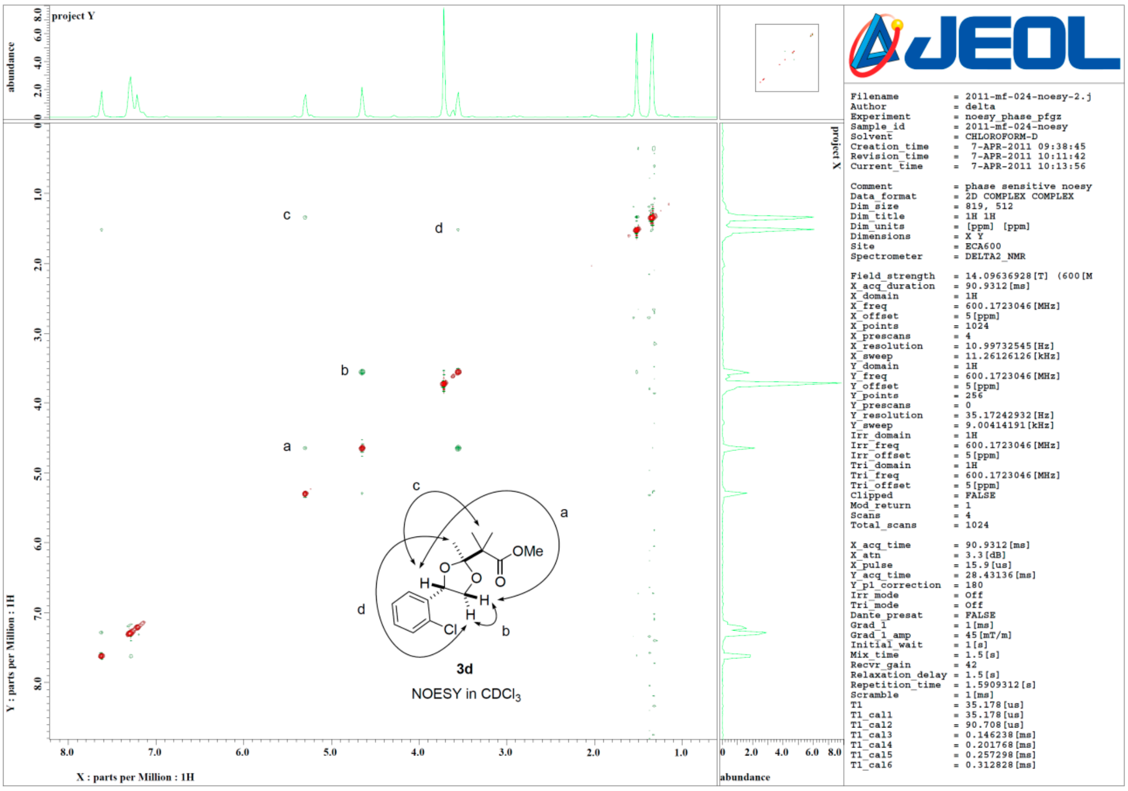

Figure S52. NOESY spectrum of **3d**.

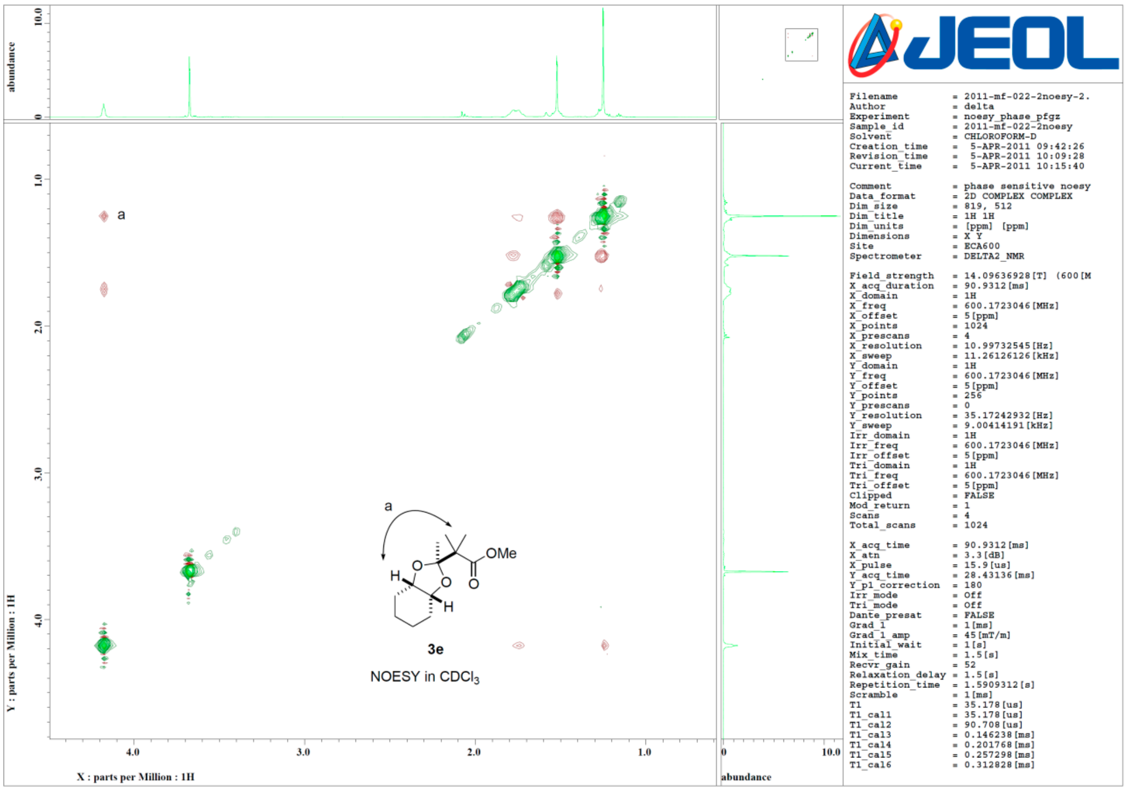

Figure S53. NOESY spectrum of **3e**.

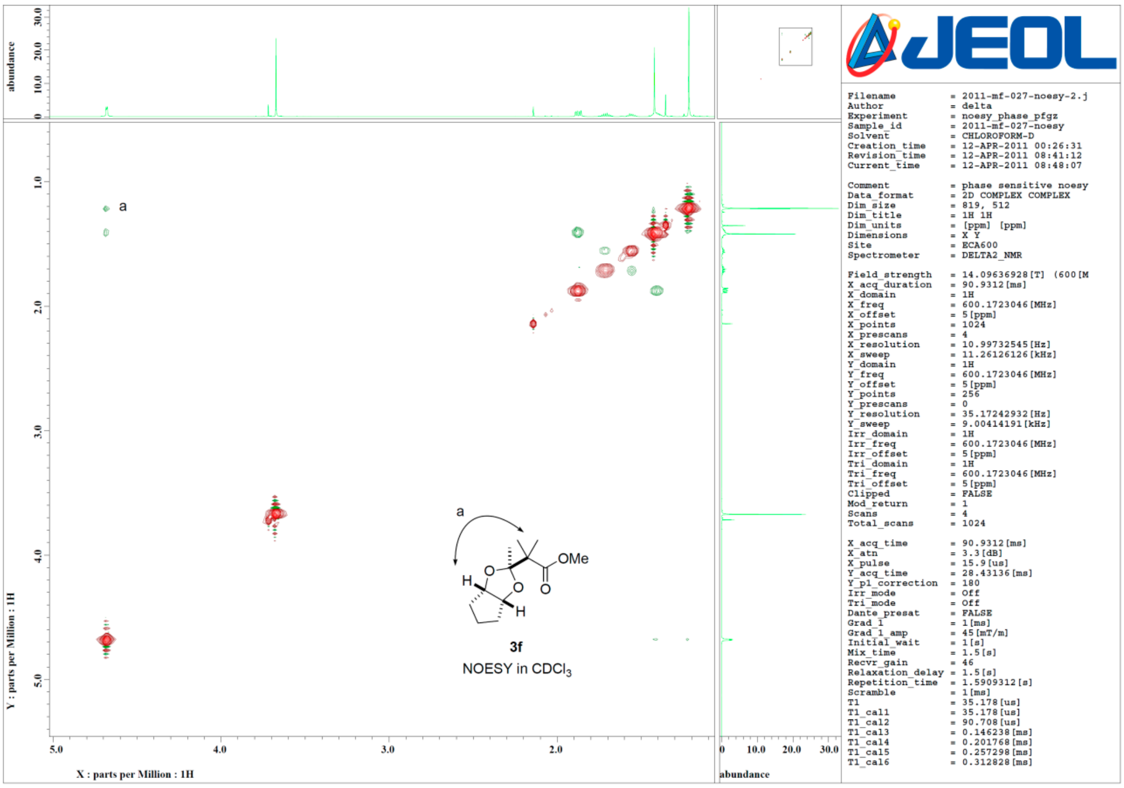

Figure S54. NOESY spectrum of **3f**.

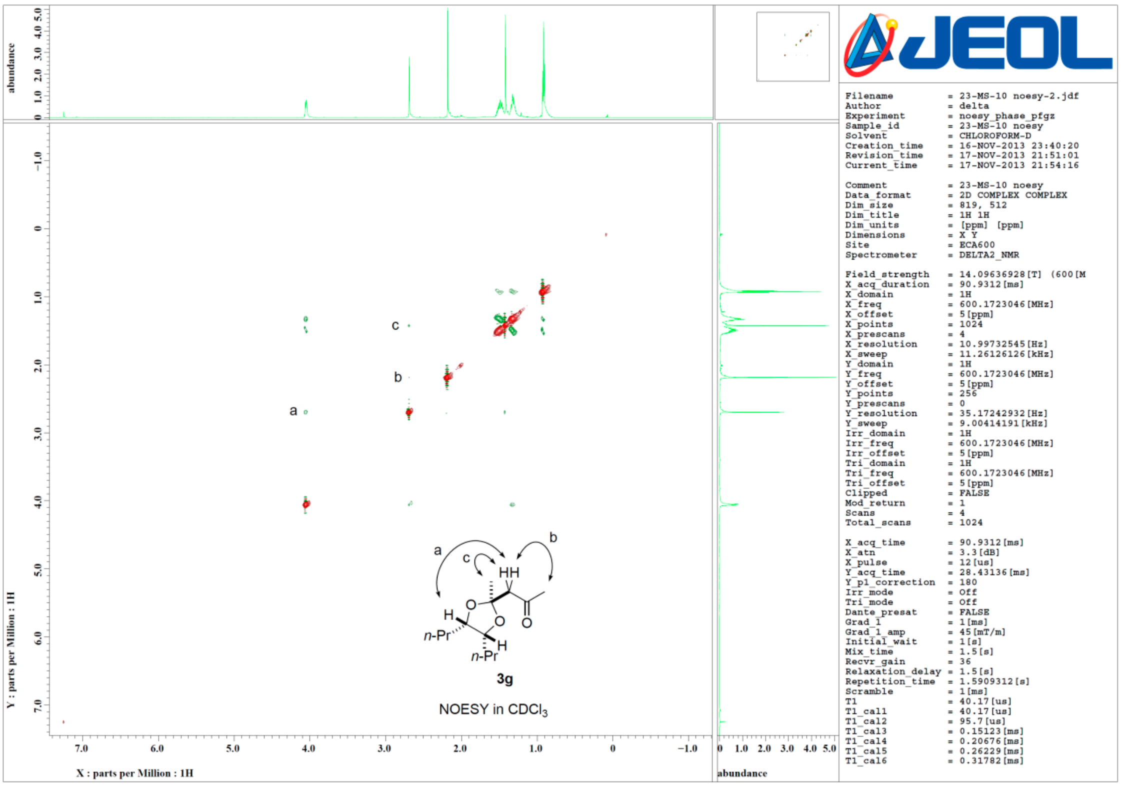

Figure S55. NOESY spectrum of **3g**.

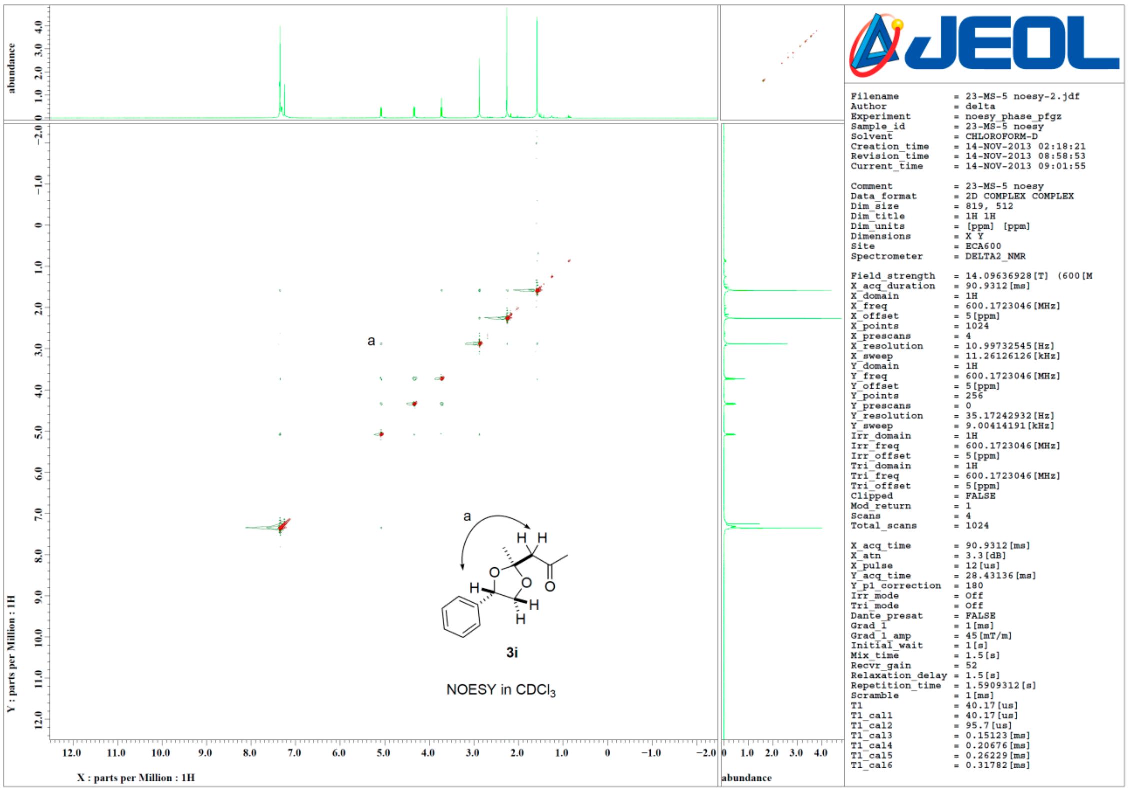

Figure S56. NOESY spectrum of **3i**.

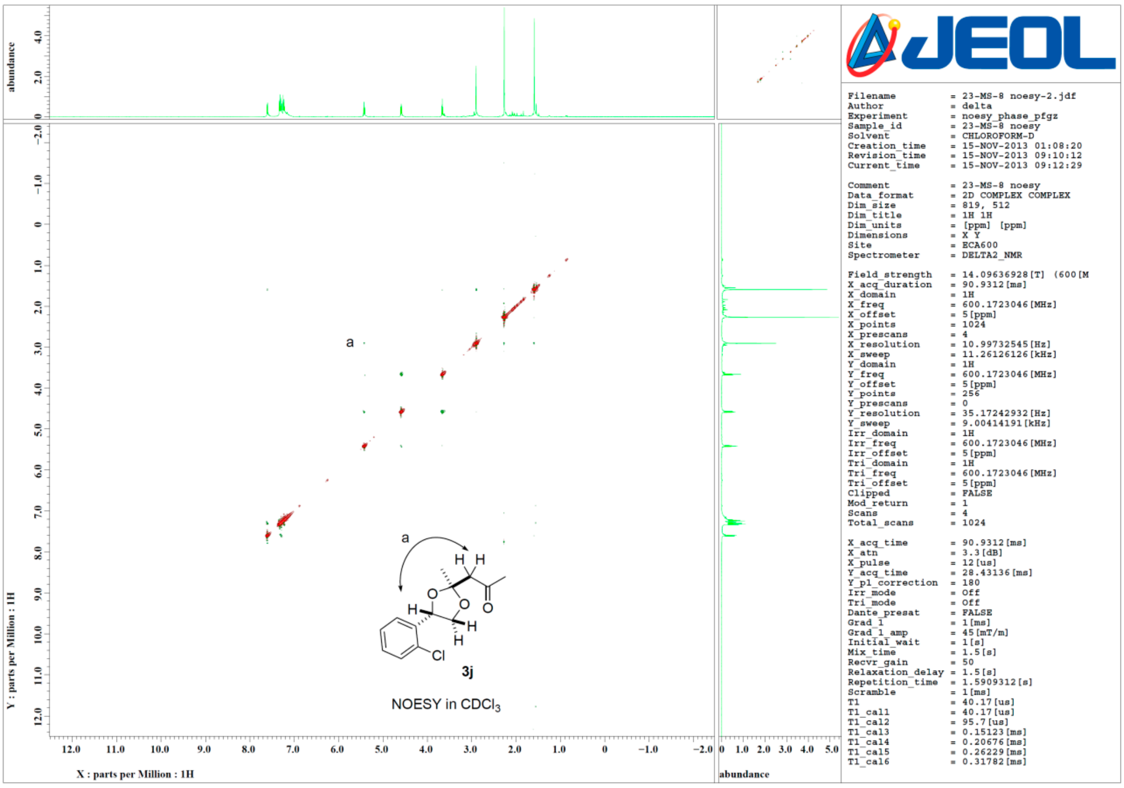

Figure S57. NOESY spectrum of **3j**.

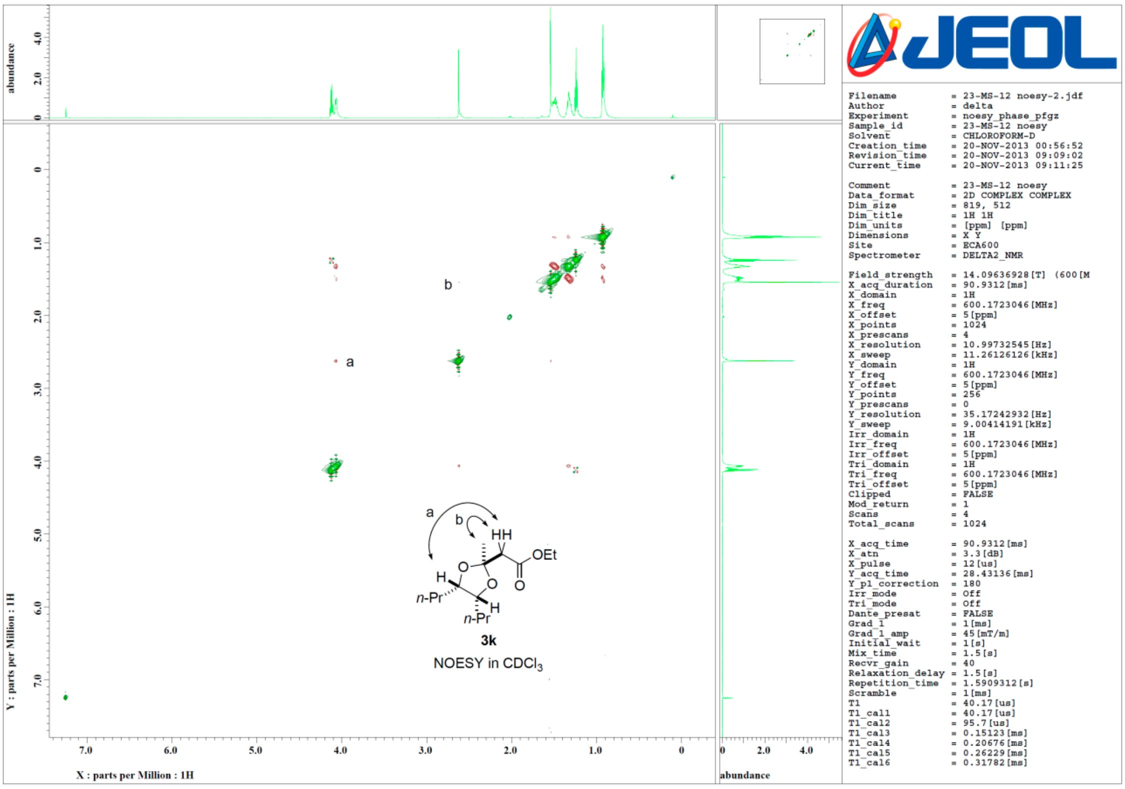

Figure S58. NOESY spectrum of **3k**.

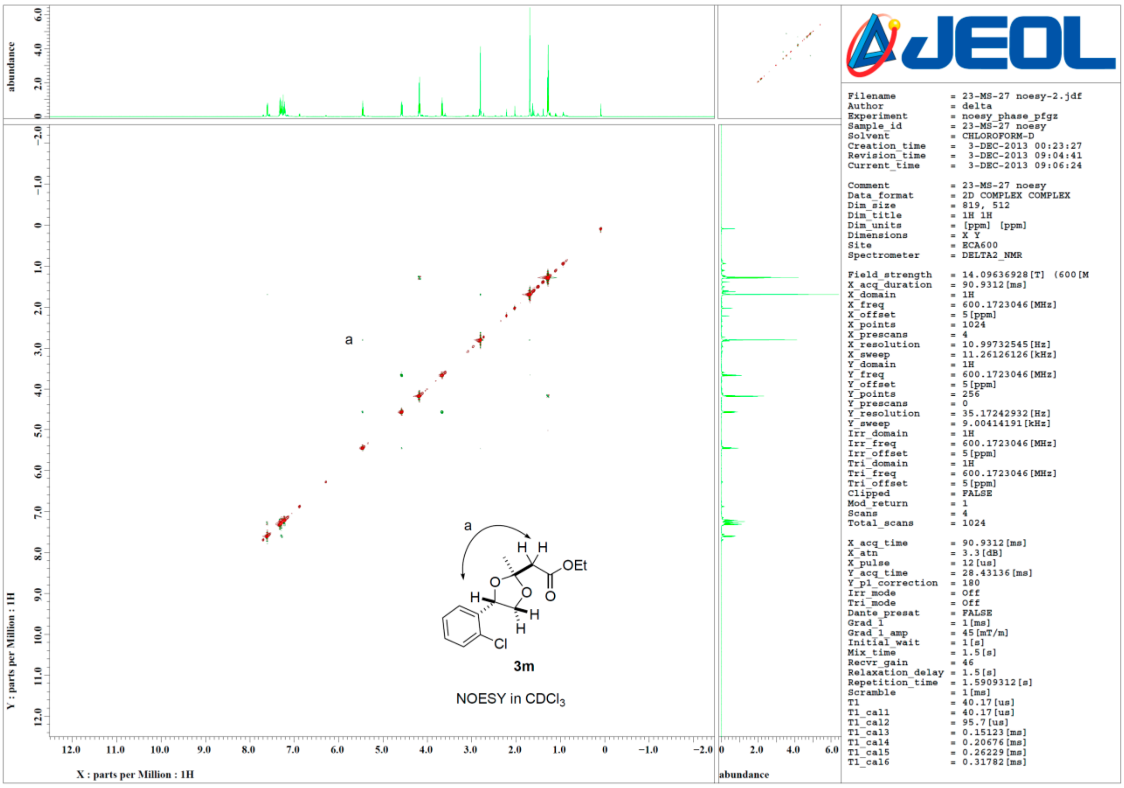

Figure S59. NOESY spectrum of **3m**.

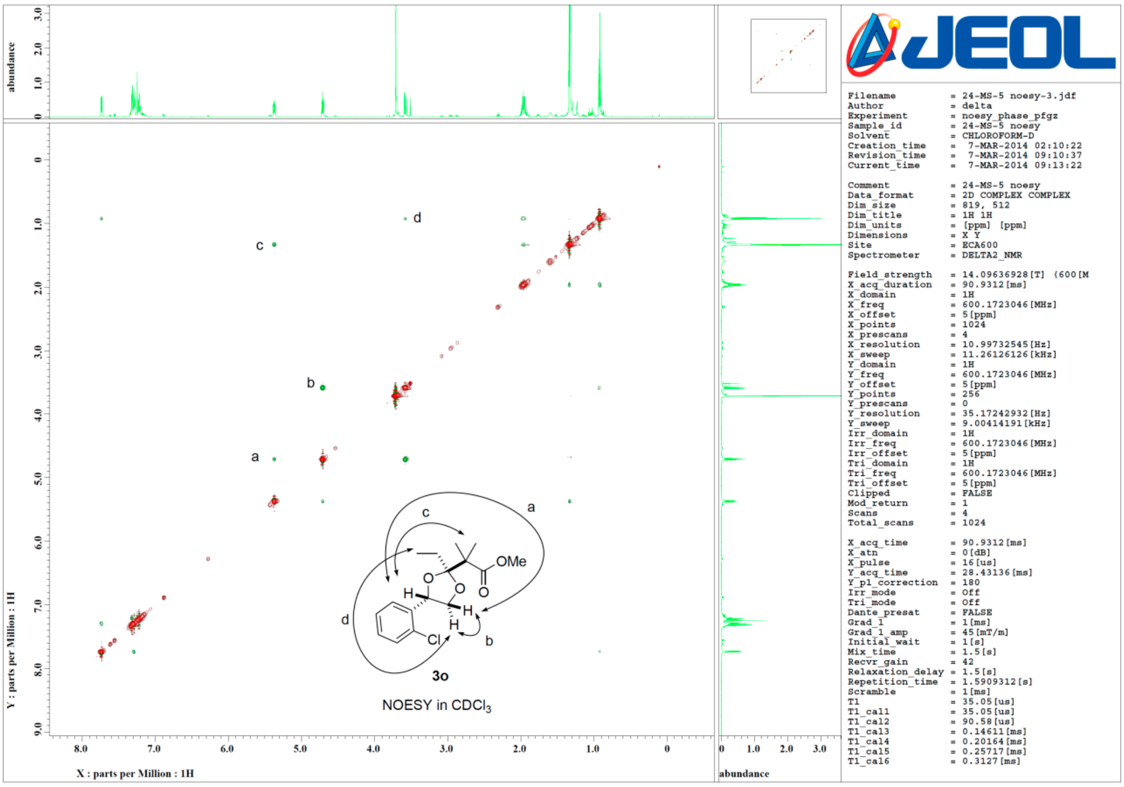

Figure S60. NOESY spectrum of **3o**.

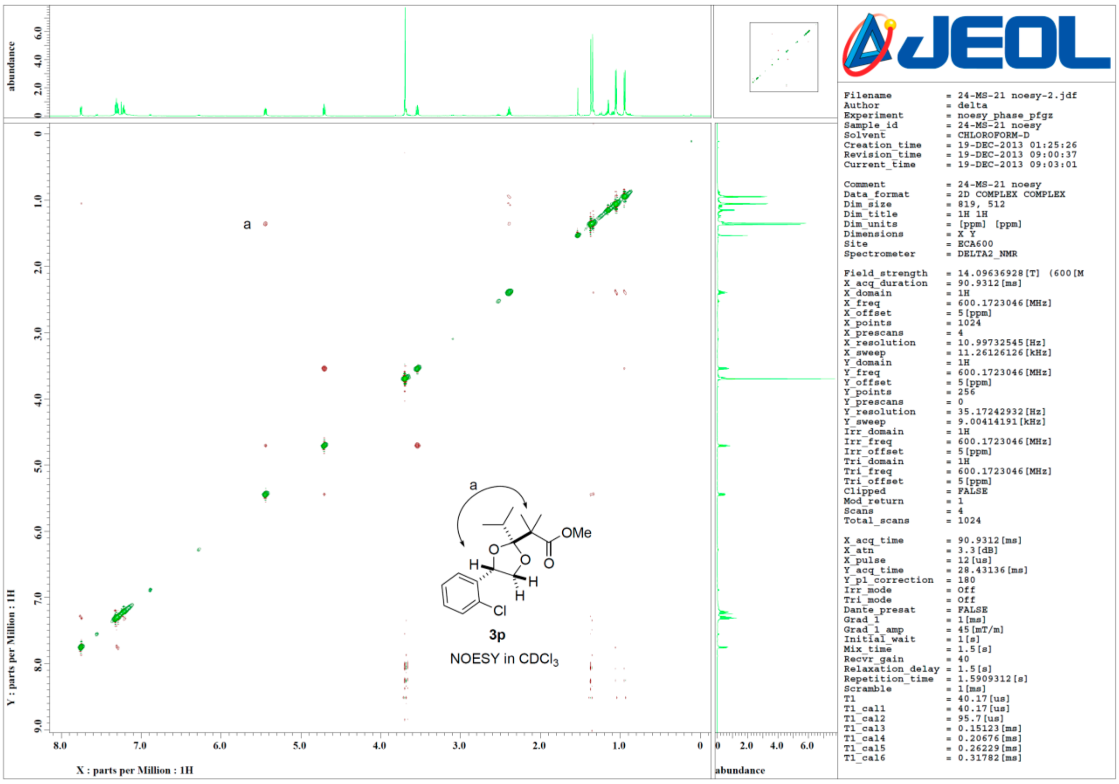

Figure S61. NOESY spectrum of **3p**.

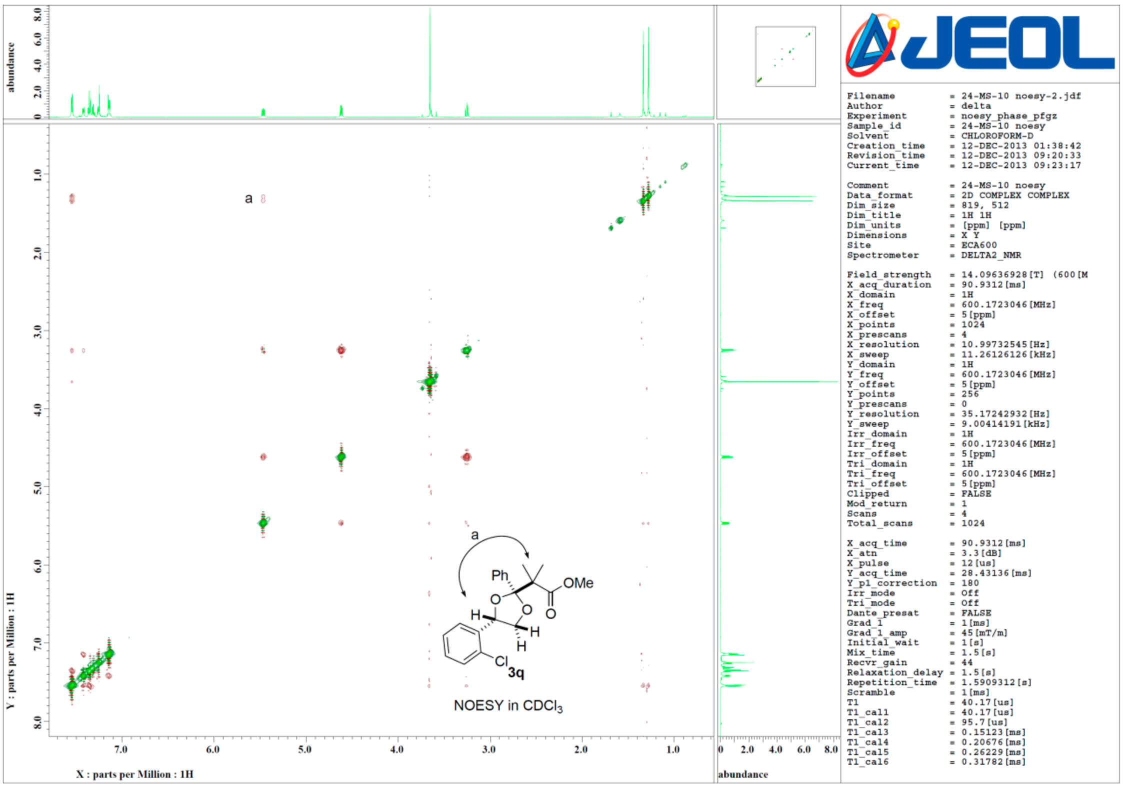

Figure S62. NOESY spectrum of **3q**.

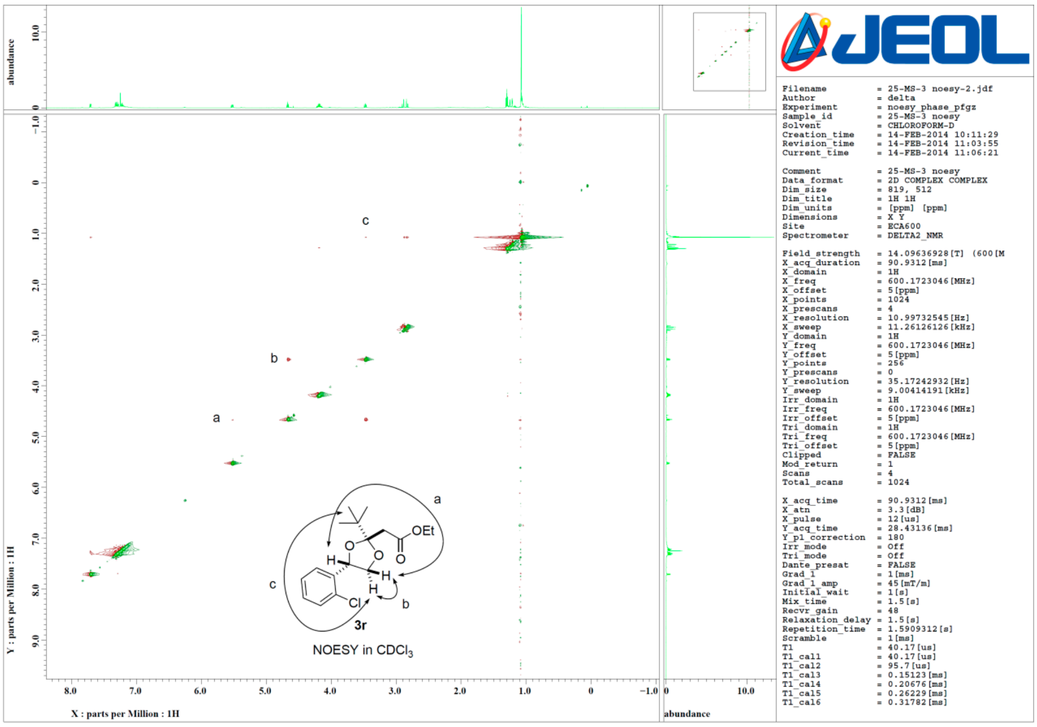

Figure S63. NOESY spectrum of **3r**.

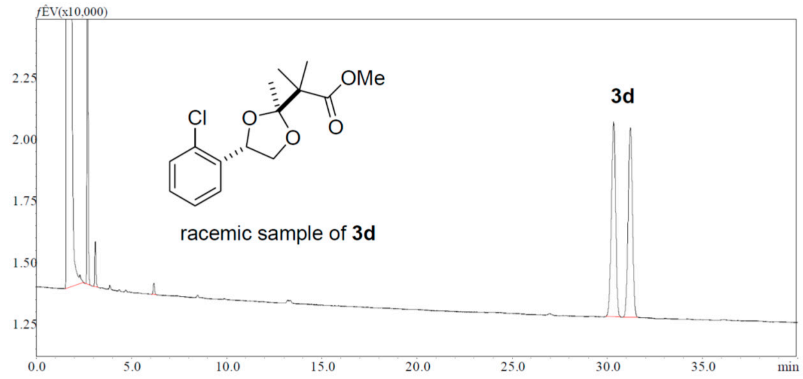

GC trace of racemic sample of **3d** (DEX–CB column at 160 °C).

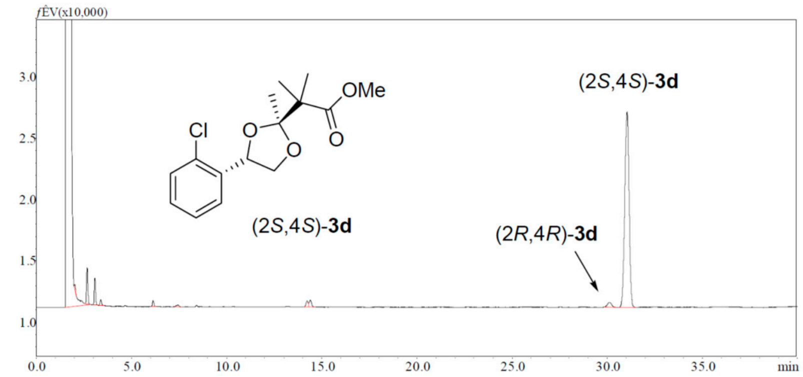

GC trace of optically active **3d** (DEX–CB column at 160 °C).

Figure S64. GC traces of **3d**.

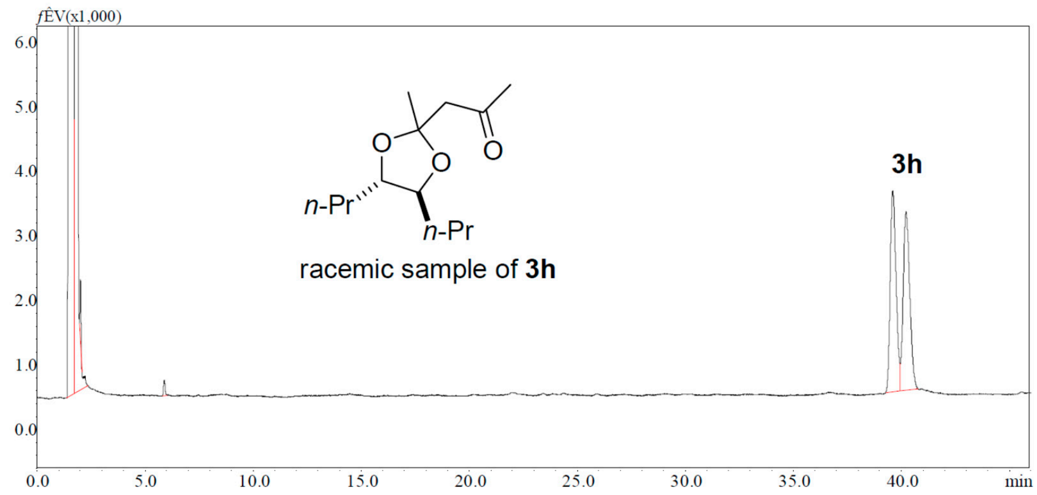

GC trace of racemic sample of **3h** (DEX–CB column 100 °C).

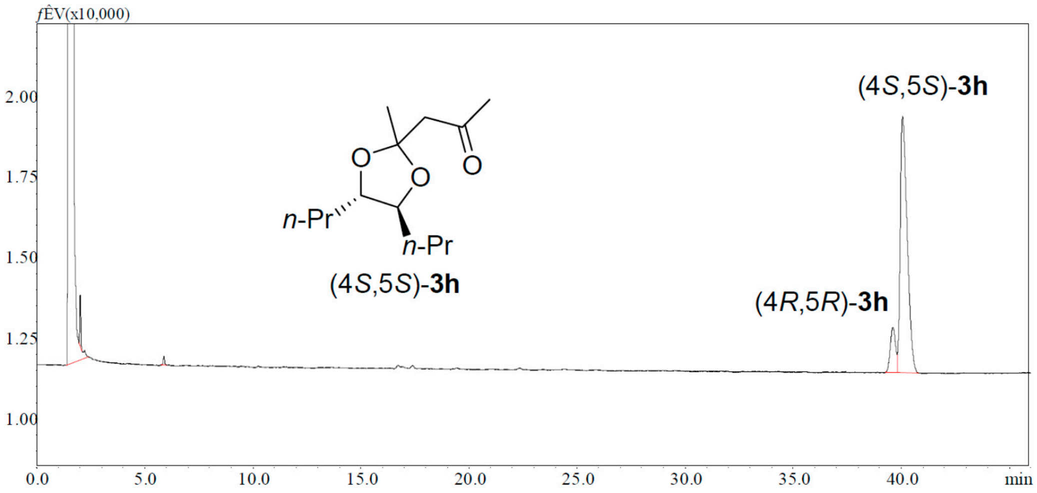

GC trace of optically active **3h** (DEX–CB column at 100 °C).

Figure S65. GC traces of **3h**.

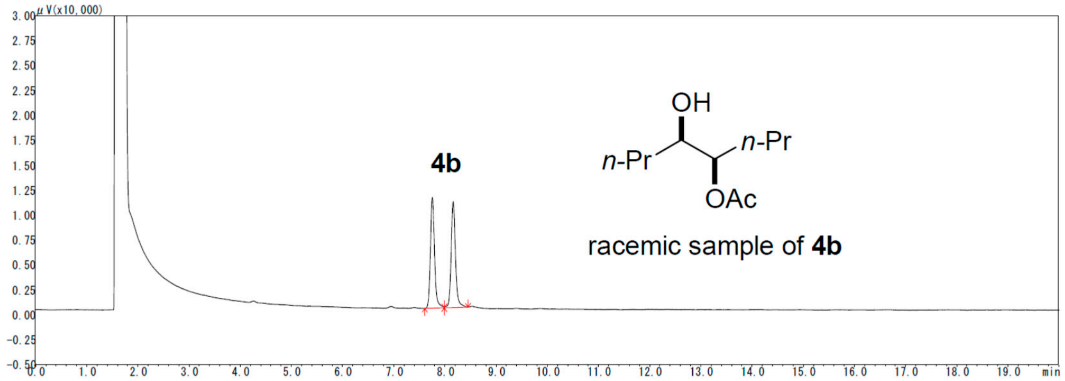

GC trace of racemic sample of **4b** (DEX–CB column at 130 °C).

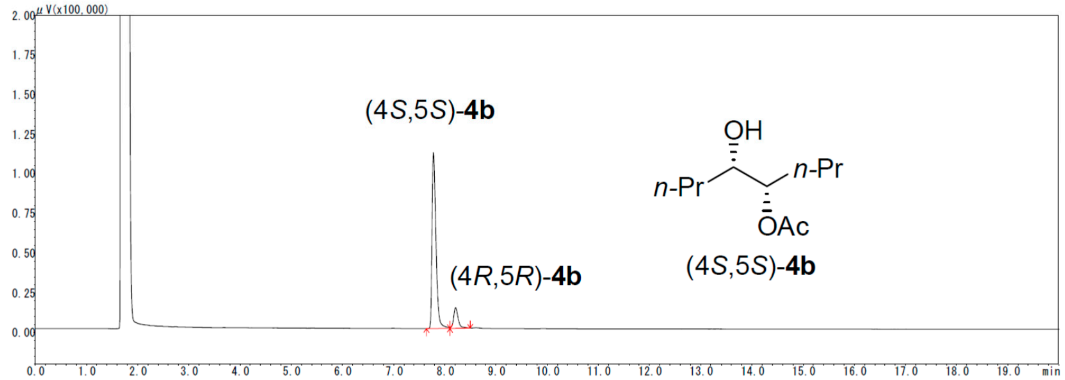

GC trace of optically active **4b** (DEX–CB column at 130 °C).

Figure S66. GC traces of **4b**.

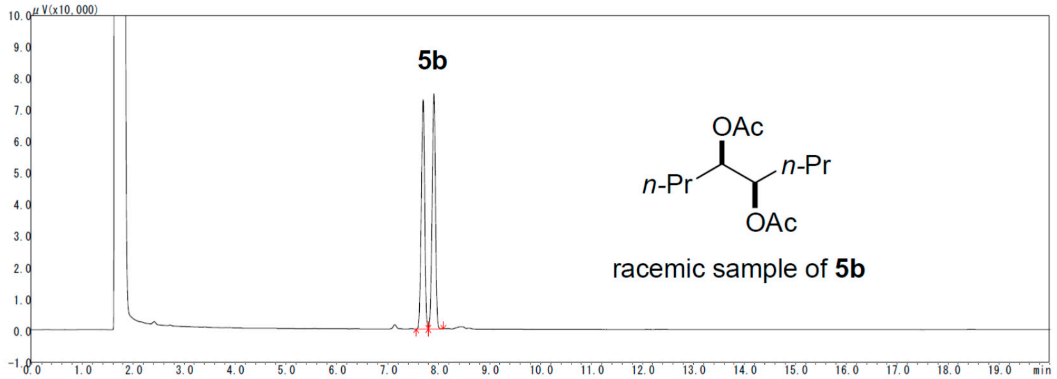

GC trace of racemic sample of **5b** (DEX–CB column at 130 °C).

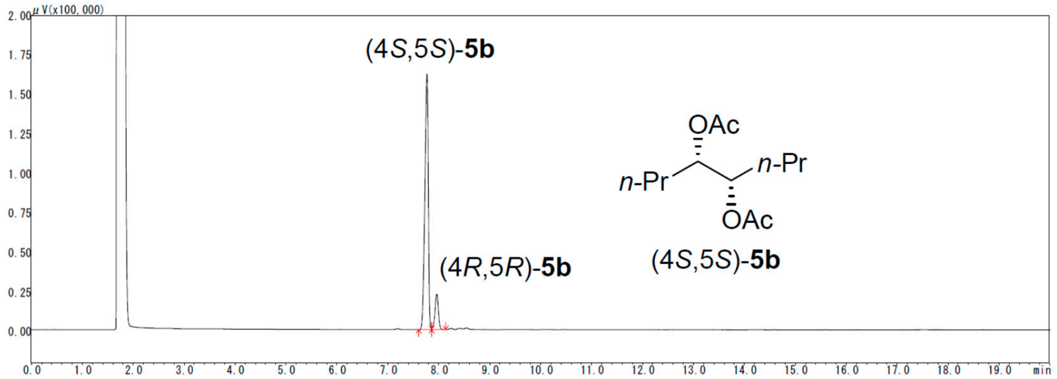

GC trace of optically active **5b** (DEX–CB column at 130 °C).

Figure S67. GC traces of **5b**.
